# Supplementary material for: Undernutrition in children aged 0–59 months by region and over time: secondary analysis of the Burkina Faso 2012–2018 National Nutrition Surveys
Source: BMJ Open. 2023 Sep 6;13(9):e066509. doi: 10.1136/bmjopen-2022-066509 (PMC10496659; doi:10.1136/bmjopen-2022-066509)
Supplement: Supplementary data [file bmjopen-2022-066509supp003.pdf]

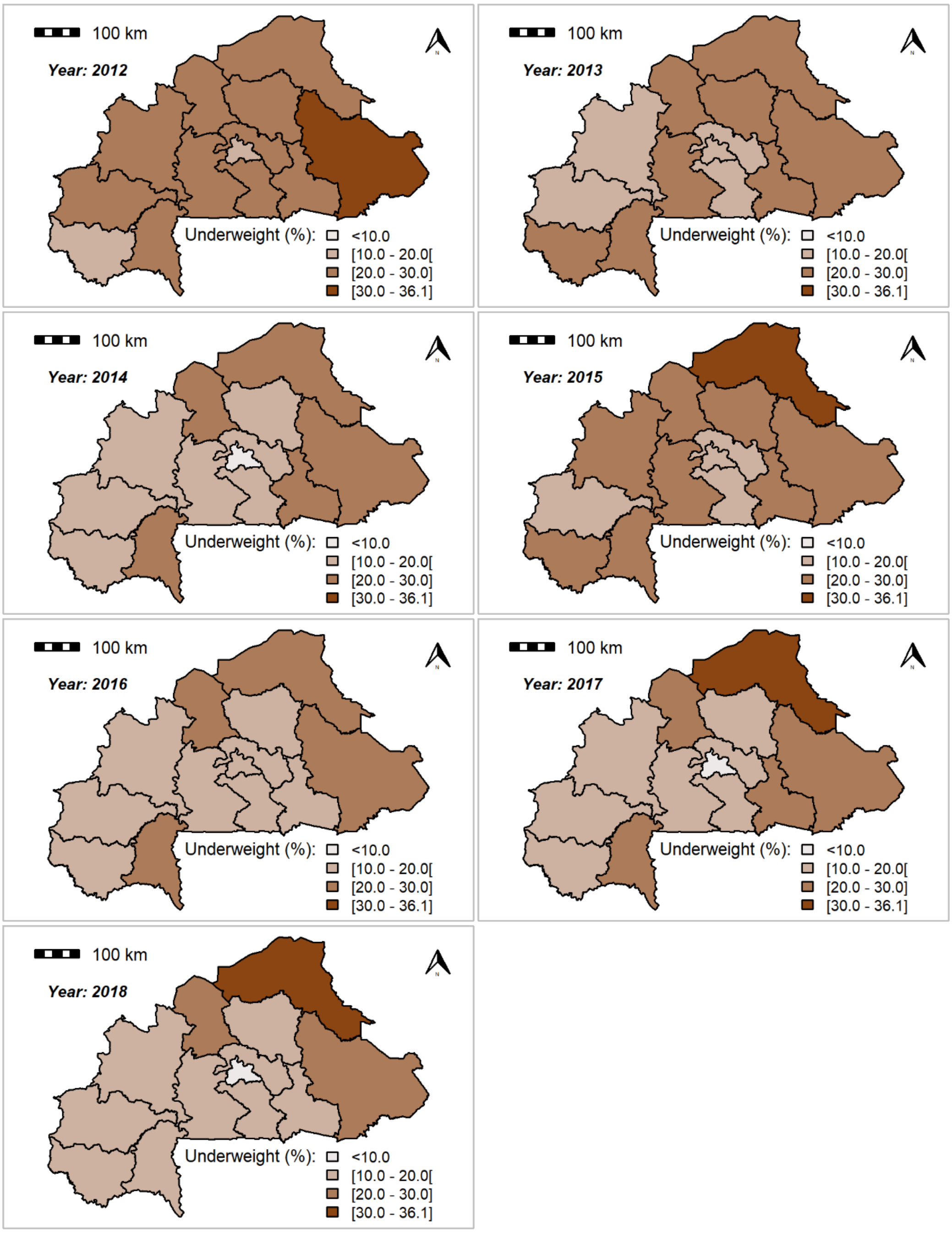

Supplemental Figure 3. Maps of geographical distribution at regional level of underweight among children aged 0–59 months surveyed in Burkina Faso, 2012 to 2018.
Maps created by Rouamba T. et al., 2021.
