# Supplementary material for: Undernutrition in children aged 0–59 months by region and over time: secondary analysis of the Burkina Faso 2012–2018 National Nutrition Surveys
Source: BMJ Open. 2023 Sep 6;13(9):e066509. doi: 10.1136/bmjopen-2022-066509 (PMC10496659; doi:10.1136/bmjopen-2022-066509)
Supplement: Supplementary data [file bmjopen-2022-066509supp009.pdf]

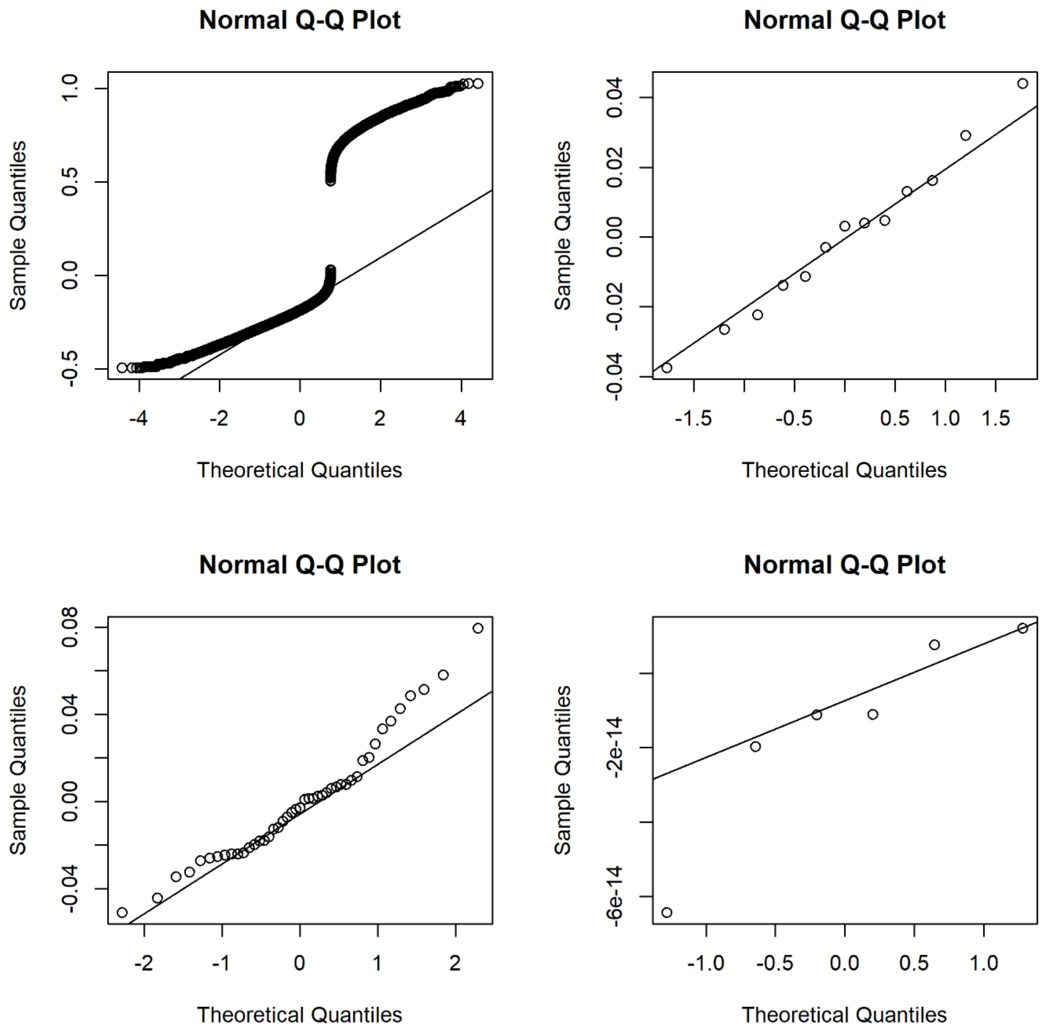

Supplemental Figure 9. Diagnostic plots for underweight multilevel model. Upper left: QQ plot of the residuals; upper right: QQ plot of the random effects (region); lower left: QQ plot of the random effects (province); lower right: QQ plot of the random effects (year)
