# Supplementary material for: Undernutrition in children aged 0–59 months by region and over time: secondary analysis of the Burkina Faso 2012–2018 National Nutrition Surveys
Source: BMJ Open. 2023 Sep 6;13(9):e066509. doi: 10.1136/bmjopen-2022-066509 (PMC10496659; doi:10.1136/bmjopen-2022-066509)
Supplement: Supplementary data [file bmjopen-2022-066509supp011.pdf]

General linear mixed models (multilevel models) with binomial distribution were used to quantify the association of independent variables with each study outcome variable (stunting, underweight and wasting). The general linear mixed model contains fixed and random effects and was formulated as follow:

$$\boldsymbol{Y_{ij}} \sim Bernoulli\ (\pi_{ij})$$

$$logit\ (\pi_{ij}) = log\left[\frac{\pi_{ij}}{1-\pi_{ij}}\right] = \beta_0 + \sum_{p=1}^{P}\beta_p x_{ij} + \sum_{q=1}^{Q}\beta_q z_j + \omega_j + \varepsilon_i$$

In this general linear model, $\boldsymbol{Y_{ij}}$ represent stunting, underweight or wasting for a child i in a cluster j. The logit (π_{ij}) was used to model the probability of success (i.e., occurrence of undernutrition) as a linear combination of observed individual characteristics (x_{ij}) and contextual characteristics (z_j) associated with an unobserved specific effect (random effect) of the province and region (ω_j). The model contains a random error for child *i* (ε_i) that does not depend to the province/region nor year. The fixed effect is composed of $\boldsymbol{\beta_0}$ (intercept), $\boldsymbol{\beta_p}$ (vector of regression coefficients associated with the individual-level variables) and $\boldsymbol{\beta_q}$ (vector of regression coefficients associated with the cluster-level variables). The unobserved specific effect or random effect ($\boldsymbol{\omega_j}$) has two components, province specific effect and region specific effect. These cluster specific effects were considered as province and region specific risk.

All statistical analyses were performed with R statistical software (R Development Core Team, R Foundation for Statistical Computing, Vienna, Austria). The descriptive statistics that accounted for the complex survey design and sampling weights were performed using "survey" package, regression models were fitted using the "lme" package.

Model Diagnostics

We assume the response was drawn from a binomial distribution the residual are independent and normally distributed. The random effects are also, independent and normally distributed. The model diagnostics was check by generating a diagnostic that plots the fitted or predicted values against the residuals.
