# Supplementary material for: Undernutrition in children aged 0–59 months by region and over time: secondary analysis of the Burkina Faso 2012–2018 National Nutrition Surveys
Source: BMJ Open. 2023 Sep 6;13(9):e066509. doi: 10.1136/bmjopen-2022-066509 (PMC10496659; doi:10.1136/bmjopen-2022-066509)
Supplement: Author's manuscript [file bmjopen-2022-066509.draft_revisions.pdf]

BMJ Open

BMJ Open is committed to open peer review. As part of this commitment we make the peer review history of every article we publish publicly available.

When an article is published we post the peer reviewers' comments and the authors' responses online. We also post the versions of the paper that were used during peer review. These are the versions that the peer review comments apply to.

The versions of the paper that follow are the versions that were submitted during the peer review process. They are not the versions of record or the final published versions. They should not be cited or distributed as the published version of this manuscript.

BMJ Open is an open access journal and the full, final, typeset and author-corrected version of record of the manuscript is available on our site with no access controls, subscription charges or pay-per-view fees (http://bmjopen.bmj.com).

If you have any questions on BMJ Open's open peer review process please email info.bmjopen@bmj.com

BMJ Open

Undernutrition in children aged 0-59 months by region and over time : Secondary analysis of the Burkina Faso 2012-2018 National Nutrition Surveys.

Journal:	*BMJ Open*
Manuscript ID	bmjopen-2022-066509
Article Type:	Original research
Date Submitted by the Author:	22-Jul-2022

Complete List of Authors:	Boua, Palwende; Institut de Recherche en Sciences de la Sante, Clinical Research Unit of Nanoro; University of the Witwatersrand Johannesburg, Sydney Brenner Institute for Molecular Biosciences<br>Rouamba , Toussaint; Institut de Recherche en Sciences de la Santé, Clinical Research Unit of Nanoro<br>Bambara, Estelle; Ministère de la Santé, Direction de la Nutrition<br>Kaboré, Saidou; Ministère de la Santé, Direction de la Nutrition<br>Compaore, Ella W. R.; Université Joseph Ki-Zerbo, Laboratoire de Biochimie, Biotechnologie, Technologie Alimentaire et Nutrition (LABIOTAN)<br>Ouedraogo, Boureima; Institut National de Statistique et de la Démographie<br>Tinto, Halidou; Institut de Recherche en Sciences de la Santé, Clinical Research Unit of Nanoro<br>Newell, Marie-Louise; University of Southampton, MRC Lifecourse Epidemiology Centre<br>Ward, Kate; University of Southampton, MRC Lifecourse Epidemiology Centre; University of Southampton, Global Health Research Institute<br>Sorgho, Hermann; Institut de Recherche en Sciences de la Santé, Clinical Research Unit of Nanoro

Keywords:	EPIDEMIOLOGY, NUTRITION & DIETETICS, PUBLIC HEALTH

SCHOLARONE™
Manuscripts

I, the Submitting Author has the right to grant and does grant on behalf of all authors of the Work (as defined in the below author licence), an exclusive licence and/or a non-exclusive licence for contributions from authors who are: i) UK Crown employees; ii) where BMJ has agreed a CC-BY licence shall apply, and/or iii) in accordance with the terms applicable for US Federal Government officers or employees acting as part of their official duties; on a worldwide, perpetual, irrevocable, royalty-free basis to BMJ Publishing Group Ltd ("BMJ") its licensees and where the relevant Journal is co-owned by BMJ to the co-owners of the Journal, to publish the Work in this journal and any other BMJ products and to exploit all rights, as set out in our licence.

The Submitting Author accepts and understands that any supply made under these terms is made by BMJ to the Submitting Author unless you are acting as an employee on behalf of your employer or a postgraduate student of an affiliated institution which is paying any applicable article publishing charge ("APC") for Open Access articles. Where the Submitting Author wishes to make the Work available on an Open Access basis (and intends to pay the relevant APC), the terms of reuse of such Open Access shall be governed by a Creative Commons licence – details of these licences and which Creative Commons licence will apply to this Work are set out in our licence referred to above.

Other than as permitted in any relevant BMJ Author's Self Archiving Policies, I confirm this Work has not been accepted for publication elsewhere, is not being considered for publication elsewhere and does not duplicate material already published. I confirm all authors consent to publication of this Work and authorise the granting of this licence.

For peer review only

Title: Undernutrition in children aged 0-59 months by region and over time : Secondary analysis of the Burkina Faso 2012-2018 National Nutrition Surveys.

Palwende R. Boua[1,2Φ*], Toussaint Rouamba[1*], Estelle Bambara[3], Saidou Kaboré[3], Ella Compaoré[4], Boureima Ouedraogo[5], Halidou Tinto[1], Marie-Louise Newell[6], Kate Ward[6,7,8], Hermann Sorgho[1] on behalf of the INPreP Study Group

[1] Clinical Research Unit of Nanoro, Institut de Recherche en Sciences de la Santé, Burkina Faso

[2] Sydney Brenner Institute for Molecular Biosciences, University of the Witwatersrand, South Africa

[3] Direction de la Nutrition, Ministère de la Santé, Burkina Faso

[4] LABIOTAN, UFR/SVT, Université Joseph Ki-Zerbo, Ouagadougou, Burkina Faso

[5] Institut National de Statistique et de la Démographie, Ouagadougou, Burkina Faso

[6] MRC Lifecourse Epidemiology Centre, University of Southampton, United Kingdom

[7] Global Health Research Institute, School of Human Development and Health, Faculty of Medicine, University of Southampton, United Kingdom

[8] School of Public Health, Faculty of Health Sciences, University of the Witwatersrand, Johannesburg, South Africa

[Φ]Corresponding author: **Palwende Romuald Boua, PhD**
Clinical Research Unit of Nanoro, Institut de Recherche en Sciences de la Santé, Burkina Faso
218 CMS Ouaga 11
romyboua@gmail.com

*Equal contribution

Word count: 3094

For peer review only

Abstract

The global burden of undernutrition remains high, responsible for significant under-five mortality in resource-limited settings. Numerous sustainable development goals (SDGs) are linked to nutrition, and nationally representative nutrition surveillance is a key activity to track progress towards SDGs and guide efficient programs.

Objectives: The aim of this study is to look at spatial and temporal trends in undernutrition in children under five years age in Burkina Faso.

Setting: We used data from annual National Nutrition Surveys using SMART methodology (anthropometry, morbidity) over seven years (2012-2018) in Burkina Faso.

Participants: Children of under-five years from households selected through systemic sampling at countrywide level.

Main outcome measures: Prevalence of stunting (height-for-age, HAZ<-2), underweight (weight-for-age, WAZ<-2) and wasting (WHZ<-2) at regional and national. We used General Linear Mixed models, adjusted by age, survey year, sex, presence of fever and/or diarrhea, and poverty index to quantify risk of undernutrition over time and by region of residence.

Results: Between 2012 and 2018, decreases were observed overall in the prevalence of stunting (stunting) decreased from 33.0% (95%CI: 32.3 – 33.8) in 2012 to 26.7% (95%CI: 26.2 – 27.3) in 2018. Underweight reduced from 24.4% (95%CI: 23.7 – 25.1) to 18.7% (95%CI: 18.2 – 19.2) for the same period and wasting decreased from 10.8% (95%CI: 10.3 – 11.3) in 2012 to 8.4% (95%CI: 8.1 – 8.8) in 2018. However, there was substantial variation across the country, with increased risk of undernutrition in the regions of Sahel, Est and Cascades mainly. High risk regions were characterized by a lower poverty index and limited access to healthcare services.

Conclusions: Our findings could inform national policymakers in refining and optimizing resource allocation based on identification of high-risk areas.

Key words: undernutrition, spatial, temporal, National Nutrional Suveys, Burkina Faso

For peer review only

Background

Globally, undernutrition (eg wasting as a sign of acute undernutrtion, and stunting as a sign of chronic undernutrition) remains an important public health issue, affecting the growth and mortality of children, especially in low- and middle-income countries [1–3]. Globally, in 2020, wasting and stunting affected an estimated 45.4 and 149.2 million children under 5 years of age, respectively [4]. [5,6]. Undernoursihed children have a higher risk of death from common childhood illness such as diarrhoea, pneumonia, and malaria [5,7,8], and longer-term are likely to experience poorer physical and cognitive development, increased risk of chronic disease in older age, with undernourished women at increased risk of delivering low birth weight babies [9–11].

In Burkina Faso, like most low-income countries, improvements in nutrition though better diet quality and access to food, continues to be a major health, socioeconomic and political challenge, and is one of the main public health priorities towards achieving Sustainable Development Goals (SDGs) 1, 2, and 3 [12]. In Burkina Faso, more than one-third of deaths among children under five years of age are directly or indirectly attributable to malnutrition. Despite the development and implementation by successive governments of numerous policies and programs to deal with malnutrition, the prevalence of undernutrition (stunting, underweight and wasting) remains high. Factors such as recurrent drought, sociocultural practices (poor dietary practices), poverty, diarrheal and febrile diseases and insufficient access to basic health services negatively affect the nutritional status of children [3,8,13–15]. In addition, there is the problem of insecurity that the country has been experiencing since 2015, leading to massive internal displacement of the population, aggravating the already very limited access to basic livelihoods, health care and nutrition services in a context of poverty in these localities [16–18 19,20].

The government, supported by its partners, has put in place a system to monitor the nutritional situation through the implementation of yearly national nutritional surveys according to the SMART methodology. This paper follows a secondary analysis of the survey databases from 2012 to 2018 in order to have an overview of temporal trends and overall spatial risk of undernutrition and identify the areas to prioritize for resource allocation according to the risk of areas.

Methods

Study setting

Burkina Faso is a landlocked country in West Africa divided into 13 regions, 45 provinces, 351 communes (49 urban and 302 rural) and 8228 villages. According to the latest census, the country has 20.5 million inhabitants, 45.3% of whom are under 15 years old and children aged 0-4 years represent 16.2% of the total population (Cinquième Récensement Général de la Population et de l'Habitat, RGPH-2019). The country is ranked 182nd out of 189 according to the Human Development Index report with more than 41.4% of its population living below the poverty line [21].

The country's epidemiological profile is marked by the persistence of a high burden of disease due to infectious disease, and by the progressive increase in the burden of non-communicable diseases. Beside the infectious and non-communicable diseases, malnutrition is one of the major diseases of public health importance [22]. The food security situation is also of concern. It is estimated that approximately 3,280,800 people are food insecure and in need of immediate humanitarian assistance, representing 15% of the country's total population [23].

For peer review only

Patient and public involvement

Because we used data from National Nutrition Surveys, it was not appropriate or possible to involve patients or the public in the design, or conduct, or reporting, or dissemination plans of our research.

Data sources

The Directorate of the Nutrition of Burkina Faso provided data (accessible through upon request). The data are from annual cross-sectional National Nutrition Surveys using the SMART (Standardized Monitoring and Assessment of Relief and Transitions) methodology. This consists of a rapid assessment survey of the food and nutrition situation among children under 5 years of age, women of childbearing age and households. For this study, we focus on anthropometric measurements collected between 2012 and 2018 (seven surveys) among population aged 0-59 months.

We examined trends in three crude indices of nutritional problems in Burkina Faso over a seven-year period, namely stunting (height/length-for-age, L/HFA), underweight (weight-for-age, WFA) and wasting (weight-for-height/length, WFH/L). Anthropometric measurements were converted into weight-for-age z-score (WAZ), height-for-age z-score (HAZ), and weight-for-height z-score (WHZ), using the 2006 World Health Organization (WHO) child growth standards [24]. Children with oedema, which can be a sign of acute malnutrition) were excluded as recommended for this analysis (**Table 1**).

Table 1. Definition of anthropometric measurements and indices in children under five

Index	Nutritional problem measured	Indicator
Height/Length-for-age (H/LFA)	Severe stunting	HAZ < −3 SD
	Moderate stunting	HAZ < −2 SD and HAZ ≥ −3 SD
	Global stunting	HAZ < −2 SD
Weight-for-age (WFA)	Severe underweight	WAZ < −3 SD
	Moderate underweight	WAZ < −2 SD and WAZ ≥ −3 SD
	Global underweight	WAZ < −2 SD
Weight-for-Height/Length (WFH/L)	Severe wasting	WHZ < −3 SD
	Moderate wasting	WHZ < −2 SD and WHZ ≥ −3 SD
	Global wasting	WHZ < −2 SD

HAZ: Length/Height-for-age z-score; WAZ: Weight-for-age z-score; WHZ: Weight-for-length/height z-score

For 2012 and 2014, the SMART survey was provincially representative for six regions (Cascades, Centre West, East, North, Sahel and South West) and regionally representative for the other seven (Boucle du Mouhoun, Centre-South, Centre, Centre-North, Centre-East, Central Plateau and Hauts-Bassins). For 2013 and 2015, the SMART survey had provincial representativeness for seven regions (Boucle du Mouhoun, Centre-South, Centre, Centre-North, Centre-East, Central Plateau and Hauts-Bassins) and regional representativeness for the other six (Cascades, Centre-West, East, North, Sahel and South-West). For 2016, the SMART survey had regional representativeness for 13 regions (resulting in smaller sample) and from 2017 onwards, the SMART survey had provincial representativeness for the 45 provinces accross the 13 regions.

Each level of representativeness constituted strata.

Outcome variables

Stunting was determined if HAZ < −2 SD, underweight as WAZ < −2 SD and wasting as WHZ < −2 SD (Table 1). We excluded z-scores with extreme values from the observed mean, the following WHO indicators were used in the final analysis: WHZ - 5 to 5; HAZ - 6 to 6; WAZ - 6 to 5.

The independent variables were individual-level characteristics mainly age in months and gender, diarrhoea and fever during the 2 weeks preceding the survey, year of the survey and cluster-level characteristics constituted by the poverty index for each province [25].

Statistical analysis

For descriptive statistics at the individual and strata levels, estimates account for the complex survey design and the sampling weights provided by *survey*. Descriptive analysis of the study population was presented using means and standard error for continuous variables and frequencies and proportions for categorical variables. These descriptive analyses were performed to estimate the crude temporal trend of prevalence nationwide and provide a yearly map for the regional disparities of undernutrition index.

General linear mixed models (multilevel models) with binomial distribution were used to quantify the association of independent variables with each study outcome variable (stunting, underweight and wasting). The general linear mixed model contains fixed and random effects and was formulated as follow:

$$\boldsymbol{Y_{ij}} \sim Bernoulli\ (\pi_{ij})$$

$$logit\ (\pi_{ij}) = log\left[\frac{\pi_{ij}}{1-\pi_{ij}}\right] = \beta_0 + \sum_{p=1}^{P} \beta_p x_{ij} + \sum_{q=1}^{Q} \beta_q z_j + \omega_j + \varepsilon_i$$

In this general linear model, $\boldsymbol{Y_{ij}}$ represent stunting, underweight or wasting for a child i in a cluster j. The logit (π_{ij}) was used to model the probability of success (i.e., occurrence of undernutrition) as a linear combination of observed individual characteristics (x_{ij}) and contextual characteristics (z_j) associated with an unobserved specific effect (random effect) of the province and region (ω_j). The model contains a random error for child *i* (ε_i) that does not depend to the province/region nor year. The fixed effect is composed of $\boldsymbol{\beta_0}$ (intercept), $\boldsymbol{\beta_p}$ (vector of regression coefficients associated with the individual-level variables) and $\boldsymbol{\beta_q}$ (vector of regression coefficients associated with the cluster-level variables). The unobserved specific effect or random effect ($\boldsymbol{\omega_j}$) has two components, province specific effect and region specific effect. These cluster specific effects were considered as province and region specific risk.

Survey weights were not used to adjust the regressions and a complete case analysis was used. All statistical analyses were performed with R statistical software (R Development Core Team, R Foundation for Statistical Computing, Vienna, Austria). The descriptive statistics that accounted for the complex survey design and sampling weights were performed using "survey" package, regression models were fitted using the "lme" package.

Results

Demographic characteristics and anthropometrical measures of children

The overall number of children aged 0–59 months assessed for their nutritional status using anthropometric measurement and included in the present analysis is showed in the Table 2. Since the

For peer review only

survey in 2016 was set up to have regionally representative estimates, 9434 children were surveyed. Gender was equally distributed through the surveys and varied between 51.6% and 51.1% for the proportion of male children. The average age of the children during the period ranged from 27 to 29 months.

The distribution of anthropometric measures such as weight, height and the z-scores with WHO as reference population (HAZ, WAZ and WHZ) from 2012 to 2018 are presented in the Table 2 below. All z-scores had a mean value below the WHO standard. HAZ values ranged from -1.50 to -1.27; WHZ values ranged from -0.70 to -0.53; WAZ values ranged from -1.33 to -1.11.

Table 2. Characteristic of Children Ages 0–59 months Surveyed at household in Burkina Faso, 2012 to 2018

	Weighted Mean (standard error)						
Index	**2012** N= 19049	**2013** N= 19170	**2014** N= 15835	**2015** N= 20408	**2016** N= 9434	**2017** N= 19409	**2018** N= 23939
Age	27.80 (0.11)	27.15 (0.12)	27.82 (0.13)	27.47 (0.11)	28.59 (0.16)	28.53 (0.11)	29.04 (0.10)
Weight	10.46 (0.02)	10.39 (0.02)	10.72 (0.03)	10.41 (0.02)	10.85 (0.03)	10.8 (0.02)	10.92 (0.02)
Height	82.08 (0.09)	81.54 (0.1)	82.68 (0.11)	81.97 (0.09)	83.38 (0.13)	83.31 (0.09)	83.75 (0.08)
HAZ	-1.49 (0.01)	-1.50 (0.01)	-1.30 (0.01)	-1.47 (0.01)	-1.30 (0.01)	-1.28 (0.01)	-1.27 (0.01)
WHZ	-0.67 (0.01)	-0.61 (0.01)	-0.53 (0.01)	-0.70 (0.01)	-0.55 (0.01)	-0.60 (0.01)	-0.56 (0.01)
WAZ	-1.31 (0.01)	-1.28 (0.01)	-1.11 (0.01)	-1.33 (0.01)	-1.12 (0.01)	-1.15 (0.01)	-1.11 (0.01)

Prevalence of undernutrition

The annual prevalence of chronic malnutrition (stunting) using HAZ ranged from 33.0% (95%CI: 32.3 – 33.8) in 2012 to 26.7% (95%CI: 26.2 – 27.3) in 2018, with a maximum of 34.2% (95%CI: 33.4 – 34.9) in 2013. In other words, between 2013 and 2018, stunting prevalence declined among children under five from 33.4% to 26.7%, a decline of 19.1% (Figure 1 (a)).

The annual prevalence of underweight (WAZ) ranged from 24.4% (95%CI: 23.7 – 25.1) in 2012 to 18.7% (95%CI: 18.2 – 19.2) in 2018, with a maximum of 25.3% (95%CI: 24.6 - 25.9) in 2015 (Figure 1 (b)). This meant that nationally, underweight decreased by 23.3% between 2012 and 2018.

The annual prevalence of wasting (WHZ) (Figure 1 (c)) ranged from 10.8% (95%CI: 10.3 – 11.3) in 2012 to 8.4% (95%CI: 8.1 – 8.8) in 2018, with a maximum of 11.7% (95%CI: 10.7 – 11.6) in 2015. In other words, between 2013 and 2018, global wasting prevalence declined among children under five from 33.4% to 26.7%, corresponding to a decline of 22.2%.

Overall, there was a reduction in prevalence of undernutrition over the years, but this was not seen in all areas. In other words, there was a spatial heterogeneity of undernutrition index through the country. During the seven year, the analysis showed a permanent high prevalence (>30%) of stunting in Sahel, and East regions (**Figure 2**), whereas the stable high prevalence of underweight (>30%) was mainly noted in the Sahel region (**Figure 3**). The high prevalence of wasting was mainly noted in the Sahel and North region (**Figure 4**).

The predicted probabilities of stunting, underweight, wasting by child's age at survey, and stratified by gender for the year 2012 to 2017 are shown in Figures S1, S2 and S3. The probability of being stunted increased with age, and was higher for male than female children, but decreased over time, i.e. between 2012 and 2018 (**Figure S1**). Similarly, predicted prevalence of underweight decreased with children age, was greater among male children and declined over the study period i.e. from 2012 to 2018 (**Figure S2**).

For peer review only - http://bmjopen.bmj.com/site/about/guidelines.xhtml

For wasting, the probability decreased with child's age and was more marked among males, but the trend over time was stable (**Figure S3**).

Potential risk factors associated with Stunting, Underweight, and Wasting from 2012 to 2017 in Burkina Faso

In multivariable analysis, allowing for xxxxxx (Table 3) males had a significantly increased risk of stunting (aOR=1.47, 95%CI: 1.43 – 1.52), underweight (aOR=1.39, 95%CI: 1.34 – 1.43) and wasting (aOR=1.38, 95%CI: 1.31 – 1.44) than females. Compared with children aged less than 5 months, older children had higher risks of stunting andunderweight, but However, the odds of wasting decreased significantly over the age of 24 months. Compared with the year 2012, the analysis showed that the risk of stunting, underweight and wasting in the subsequent years, i.e. 2013 to 2018, decreased significantly overall, except for the year 2015 for wasting. Furthermore, diarrhoea was associated with high odds of stunting (aOR=1.08, 95%CI: 1.05 – 1.12), underweight (aOR=1.32, 95%CI: 1.27 – 1.37) and wasting (aOR=1.46, 95%CI: 1.39 – 1.54). This association was similar with the fever. There was no significant association between wasting and poverty index (aOR=1.03, 95%CI: 0.96 – 1.10), whereas stunting and underweight were significantly associated with the poverty index.

Table 3. Potential risk factors associated with Stunting, Underweight, and Wasting from 2012 to 2017 in Burkina Faso

	Stunting			Underweight			Wasting		
Predictors	aOR	95%CI	p	aOR	95%CI	p	aOR	95%CI	p
Gender									
Female	1			1			1		
Male	1.39	1.35 – 1.43	<0.001	1.31	1.27 – 1.35	<0.001	1.37	1.31 – 1.43	<0.001
Age in Month									
00 - 05									
06 - 11	1.51	1.39 – 1.63	<0.001	1.96	1.82 – 2.11	<0.001	1.67	1.53 – 1.82	<0.001
12 - 23	3.74	3.49 – 4.00	<0.001	2.56	2.39 – 2.73	<0.001	1.53	1.41 – 1.65	<0.001
24 - 35	4.94	4.62 – 5.28	<0.001	2.28	2.13 – 2.44	<0.001	0.83	0.76 – 0.90	<0.001
36 - 47	4.57	4.27 – 4.89	<0.001	1.67	1.56 – 1.79	<0.001	0.46	0.41 – 0.50	<0.001
48 - 59	3.44	3.20 – 3.68	<0.001	1.58	1.47 – 1.69	<0.001	0.49	0.44 – 0.54	<0.001
Year of survey									
2012	1			1			1		
2013	0.89	0.85 – 0.94	<0.001	0.79	0.75 – 0.84	<0.001	0.72	0.66 – 0.78	<0.001
2014	0.78	0.75 – 0.82	<0.001	0.72	0.69 – 0.76	<0.001	0.73	0.68 – 0.79	<0.001
2015	0.87	0.82 – 0.91	<0.001	0.95	0.90 – 1.00	0.063	1.00	0.92 – 1.08	0.902
2016	0.73	0.69 – 0.78	<0.001	0.74	0.69 – 0.79	<0.001	0.70	0.63 – 0.77	<0.001
2017	0.67	0.64 – 0.71	<0.001	0.71	0.68 – 0.75	<0.001	0.86	0.80 – 0.93	<0.001
Diarrhea									
No	1			1			1		
Yes	1.19	1.14 – 1.23	<0.001	1.36	1.31 – 1.42	<0.001	1.39	1.32 – 1.47	<0.001
Fever									
No	1			1			1		
Yes	1.08	1.05 – 1.12	<0.001	1.31	1.27 – 1.35	<0.001	1.48	1.41 – 1.55	<0.001
Poverty Index in %	1.17	1.09 – 1.26	<0.001	1.15	1.08 – 1.22	<0.001	1.03	0.96 – 1.10	0.456
**Random Effects**									
σ2		3.29			3.29			3.29	
τ00									
Provinces		0.03			0.03			0.04	
Region		0.03			0.01			0.00	
ICC		0.02			0.01			0.01	
N Region		13			13			13	
N Provinces		45			45			45	

For peer review only

Observations	83161	83161	83161

Estimation of province and region specific risk

The province spatial risk of stunting, underweight and wasting was shown to be heterogeneous throughout the country. Figure 5 displays the specific risks (or odds) of each province compared with the national mean odds ratio (aOR = 1).

For stunting, the provinces with high odds were located in eight provinces namely Gourma, Ioba, Kenedougou, Kossi, Lorum, Seno and Yagha provinces, whereas, the provinces with lower odds were located in four provinces namely, Bales, Passore, Tuy, and Zounweogo provinces (**Figure 5 (a)**).

Regarding underweight, the provinces with high odds were located in nine provinces namely Gourma, Ioba, Kossi, Kourweogo, Lorum, Oudalan, Seno, Tapoa, Yagha provinces, whereas, the provinces with lower odds were located in four provinces namely, Passore, Poni, Sourou and Zounweogo provinces (**Figure 5 (b)**).

Regarding wasting, the provinces with high odds were located in 11 provinces namely, Bam, Ioba, Kossi, Kourweogo, Lorum, Nayala, Oudalan, Sanguie, Seno, Tapoa and Yatenga provinces whereas, the provinces with lower odds were located in eight provinces namely, Boulgou, Comoe, Houet, Koulpelgo, Leraba, Poni, Sourou, and Zounweogo province (**Figure 5 (c)**).

Further, the analysis also indicated some regions where the odds was statistically significant higher (Sahel and Cascades) or lower (Centre-sud, centre-Ouest and Boucle de Bouhoun) for stunting (**Figure 6 (a)**). For underweight, only the region of the Sahel exhibited higher odds (**Figure 6 (b)**). Finally, regarding wasting, no region exhibited a higher or lower risk (**Figure 6 (c)**).

Discussion

This study undertook a secondary analysis using data from annual cross-sectional National Nutrition Survey (NNS) using SMART methodology to generate estimates that could help national policymakers in refining and optimizing resource allocation based on identification of high-risk areas. The analysis found that undernutrition remains very high, although there was a decrease in prevalence and risk over the years; the spatial risk to experience an undernutrition event is not uniform throughout the country. This decrease of all undernutrition prevalence has been observed in other sub-Saharan Africa countries depite predicted stagnation in the region for stunting until 2020 [26–28].

In previous analysis, De Onis and collaborators predicted that in Africa, the prevalence of stunting was going to stagnate in next coming decade from 2010, and specifically in Western Africa [28]. Our results showed a decrease of stunting prevalence of 6.7 between 2012 and 2018.

Sex of child (male), two weeks morbidity (fever, diarrhea) were factors associated with the three forms of undernutrition; whereas poverty measured through wealth index was associated with stunting and underweight but not wasting. In their analysis of Nigeria Demographic and Health Survey (NDHS), Akombi et al. found the same factors associated to stunting[29].

Living in certain provinces and/or regions of the country is conferring higher risk of suffering from undernutrition. Similar findings were reported in Côte d'Ivoire, Uganda and Nigeria [27,29,30]. In Burkina Faso, these areas were the one with the highest proportion of the population living more than 10 Km away from the primary health center (first level of referal) [22]. This means that access to health care is associated with nutritional status. The persistent prevalence of undernutrition translate a need, not just for the community management of wasting programmes which have been shown to be effective, but for effective prevention programmes [31].

For peer review only

Prior to the recent threats from terrorism, Burkina Faso had already endured decade of a fragile food security situation due to droughts and floods, affecting food security and nutrition, especially in the north part of the country. Restrictions on the movement of people and goods, as well as limited access to natural resources and regional or international markets, had placed a heavy burden on local food systems and people's livelihoods. The food insecurity situation in Burkina Faso is currently characterized by a high level of insecurity. The populations of the Sahel zone are the most affected. Malnutrition is chronic and its causes are structural and complex. They are the result of a combination of several factors, including a short agricultural production season lasting four months of the year, climatic vulnerability, the depletion of natural resources, a chronic shortage of agricultural production, and very little access to basic social services due to the weak state presence.

Insecurity marked by attacks from terrorist groups and the increase in inter-community conflicts exacerbate food and nutrition insecurity in the Sahel and North Central regions by reducing the already fragile livelihoods of populations that depend mainly on agriculture and pastoralism. Insecurity limits access to fields and agricultural production. The resulting road closures affect the supply of agricultural products to markets, reduce the availability of agricultural products, limit access due to price fluctuations, and hinder transhumance and access of animals to pasture within Burkina and to Mali and Niger. The looting of food reserves and livestock reduces the resilience of communities affected by this insecurity. In addition, many health facilities are closed or operating at minimal capacity in areas affected by insecurity, reducing access to health services, including those for severely malnourished children. This has forced people to move to other parts of the country (almost one million internally displaced persons (IDPs) according to the United Nations Office for the Coordination of Humanitarian Affairs), resulting in overcrowding in host towns. While some IDPs reside in camps, more than 80% of IDPs are staying with host families whose food supplies are rapidly running out.

In our study, we found that poverty index is not associated with wasting, that mean acute malnutrition is due to an underlying disease causing abnormal nutrient loss, increased energy expenditure, or decreased food intake. Based on its etiology, malnutrition is either illness related (one or more diseases or injuries directly result in nutrient imbalance) or caused by environmental/behavioral factors associated with decreased nutrient intake and/or delivery [11,32,33]. Therefore, primary acute malnutrition is mostly social rather than biomedical in origin, but it is also multifactorial. For example, poor water quality, sanitation and hygiene practices are increasingly believed to be the cause of the condition called "environmental enteropathy" that contributes to acute malnutrition in childhood [33].

Although this study provided insight on the temporal trends and overall spatial risk in undernutrition among children aged 0-59 months between 2012 and 2018 in Burkina Faso, this study is influenced by several limitations. Our study did not cover all the National Nutrition Surveys from 2009 to 2021, due to data availability, therefore our finding are only relevant for the studied period. The analysis relies on survey data but not a cohort, hence causality cannot be inferred.

Conclusion

The study found that undernutrition prevalence remains high and marked spatial disparities. Overall, there is a decreased of prevalence throughout the year. It is important to note that we found low risk area that need to sustain and reinforce the control effort. The high-risk area, need to be directed more resources and reinforce the control effort. Morbidity (fever and diarrhea) constitutes important associated factors and need prompt and effective management with particular focus on children over 6 months. Further research should include birth cohort to allow causality inference for undernutrition in Burkina Faso. Additionally, surveillance system should be implemented towards IDPs.

For peer review only - http://bmjopen.bmj.com/site/about/guidelines.xhtml

Acknowledgements

The authors would like to thank the Ministry of Health and Public Hygiene for allowing access to the data.

Funding

This research was funded by the National Institute for Health Research (NIHR) (17/63/154) using UK aid from the UK Government to support global health research. The views expressed in this publication are those of the author(s) and not necessarily those of the NIHR or the UK Department of Health and Social Care.

Conflict of interest

The authors declare that they have no competing interests.

Authorship

H.S., M.L.N., P.R.B., H.T. and K.W. designed the WP5 study. H.S., P.R.B., H.T. and K.W. supervised the implementation of the study and guided the manuscript conception. P.R.B. and TR analysed the data, carried out the literature review and drafted the paper. All authors reviewed and approved the final manuscript.

Ethics

This study is part of the of the INPreP3 Study and has been approved by the National Health Ethics Committee in Burkina Faso, Approval number 2018-12-156.

Data sharing

Access to data is available upon request to the Ministry of Health and Public Hygiene.

For peer review only

References

1. FAO, IFAD, UNICEF & WFP. *The State of Food Security and Nutrition in the World 2021. Transforming food systems for food security, improved nutrition and affordable healthy diets for all.* (2021). doi:10.4060/cb4474en
2. Development Initiatives. *The burden of malnutrition - Global Nutrition Report*. (2018).
3. Myatt, M. *et al.* Children who are both wasted and stunted are also underweight and have a high risk of death: A descriptive epidemiology of multiple anthropometric deficits using data from 51 countries. *Arch. Public Heal.* **76**, (2018).
4. UNICEF / WHO / World Bank Group. *UNICEF-WHO-The World Bank: Joint child malnutrition estimates – levels and trends – 2021 edition - UNICEF DATA*. (2021).
5. Children: improving survival and well-being.
6. Pelletier, D. L., Frongillo, E. A. & Habicht, J. P. Epidemiologic evidence for a potentiating effect of malnutrition on child mortality. *Am. J. Public Health* **83**, 1130–1133 (1993).
7. Black, R. E. *et al.* Maternal and child undernutrition: global and regional exposures and health consequences. *Lancet* **371**, 243–260 (2008).
8. Black, R. E. *et al.* Maternal and child undernutrition and overweight in low-income and middle-income countries. *Lancet* **382**, 427–451 (2013).
9. McFie, J. & Welbourn, H. F. Effect of Malnutrition in Infancy on the Development of Bone,

Muscle and Fat. *J. Nutr.* **76**, 97–105 (1962).
10. Darsene, H., Geleto, A., Gebeyehu, A. & Meseret, S. Magnitude and predictors of undernutrition among children aged six to fifty nine months in Ethiopia: A cross sectional study. *Arch. Public Heal.* **75**, (2017).
11. Shahrin, L., Chisti, M. J. & Ahmed, T. 3.1 Primary and secondary malnutrition. *World Rev. Nutr. Diet.* **113**, 139–146 (2015).
12. United Nations Sustainable Development Goals (SDGs).
13. McDonald, C. M. *et al.* The effect of multiple anthropometric deficits on child mortality: Meta-analysis of individual data in 10 prospective studies from developing countries. *Am. J. Clin. Nutr.* **97**, 896–901 (2013).
14. Khara, T., Mwangome, M., Ngari, M. & Dolan, C. Children concurrently wasted and stunted: A meta-analysis of prevalence data of children 6–59 months from 84 countries. *Matern. Child Nutr.* **14**, (2018).
15. Garenne, M. *et al.* Incidence and duration of severe wasting in two African populations. *Public Health Nutr.* **12**, 1974–1982 (2009).
16. Davycas, UNICEF, WFP & Ministry of Health. *Nutrition survey in communities and sites with internally displaced persons (IDPs) in Burkina Faso*. (2020).
17. Ridde, V., Lechat, L. & Meda, I. B. Terrorist attack of 15 January 2016 in Ouagadougou: how resilient was Burkina Faso's health system? *BMJ Glob. Heal.* **1**, e000056 (2016).
18. International Crisis Group. The Social Roots of Jihadist Violence in Burkina Faso's North | Crisis Group. *Africa Report N°254* (2017).
19. Save the Children, DS/DRS de Kaya & Lionella Fieschi. *Prise en charge Communautaire de la Malnutrition Aiguë (PCMA): Évaluation Semi-Quantitative de l'Accessibilité et de la Couverture (SQUEAC)*. (2010).
20. Ministry of Health. *Guide pour le suivi et la gestion de la prise en charge de la malnutrition aiguë.* (2009).
21. The World Factbook. The World Factbook — Central Intelligence Agency. Burkina Faso population projection.
22. Ministère de la Santé Burkina Faso (DGESS). *Annuaire statistique 2020. Annuaire statistiaue 2018* (2020).
23. Ministère de la Santé. *Enquête Nutritionnelle Nationale (SMART-2020)*. (2020).
24. World Health Organization. *Length/height-for-age, weight-for-age, weight-for-length, weight-for-height and body mass index-for-age Methods and development*. (2006).
25. Zida, Y. & Kambou, S. H. *Cartographie de la pauvreté et des inégalités au Burkina Faso*. (2014).
26. Masibo, P. K. & Makoka, D. Trends and determinants of undernutrition among young Kenyan children: Kenya Demographic and Health Survey; 1993, 1998, 2003 and 2008-2009. *Public Health Nutr.* **15**, 1715–1727 (2012).
27. Barankanira, E., Molinari, N., Msellati, P., Laurent, C. & Bork, K. A. Stunting among children under 3 years of age in Côte d'Ivoire: Spatial and temporal variations between 1994 and 2011. *Public Health Nutr.* **20**, 1627–1639 (2017).
28. De Onis, M., Blössner, M. & Borghi, E. Prevalence and trends of stunting among pre-school children, 1990-2020. *Public Health Nutr.* **15**, 142–148 (2012).
29. Akombi, B. J. *et al.* Stunting and severe stunting among children under-5 years in Nigeria: A multilevel analysis. *BMC Pediatr.* **17**, 1–16 (2017).
30. Yang, Y. Y. *et al.* Trends and determinants of stunting among under-5s: Evidence from the 1995, 2001, 2006 and 2011 Uganda Demographic and Health Surveys. *Public Health Nutr.* **21**, 2915–2928 (2018).
31. Ruel, M. T. *et al.* Age-based preventive targeting of food assistance and behaviour change and communication for reduction of childhood undernutrition in Haiti: a cluster randomised trial. *Lancet (London, England)* **371**, 588–595 (2008).
32. Dipasquale, V., Cucinotta, U. & Romano, C. Acute Malnutrition in Children: Pathophysiology,

For peer review only

Clinical Effects and Treatment. *Nutrients* **12**, 1–9 (2020).
33. Gelli, A. *et al*. Improving diets and nutrition through an integrated poultry value chain and nutrition intervention (SELEVER) in Burkina Faso: Study protocol for a randomized trial. *Trials* **18**, (2017).

For peer review only

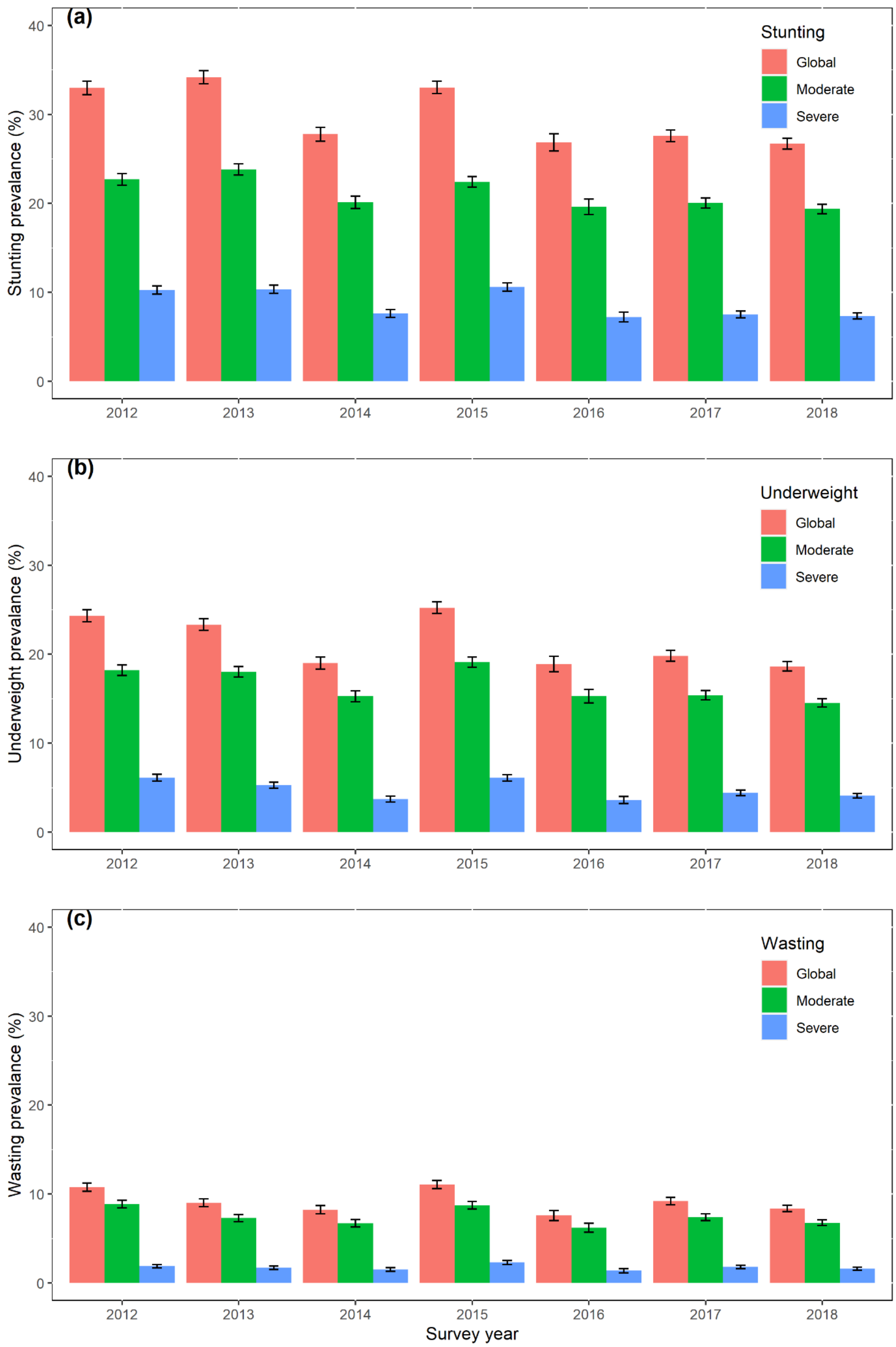

Figure 1. Prevalence of stunting (a), underweight (b) and wasting (c). The plots demonstrate changes from 2012 to 2018. The error bars mark the 95% confidence intervals in panels a, b and c.

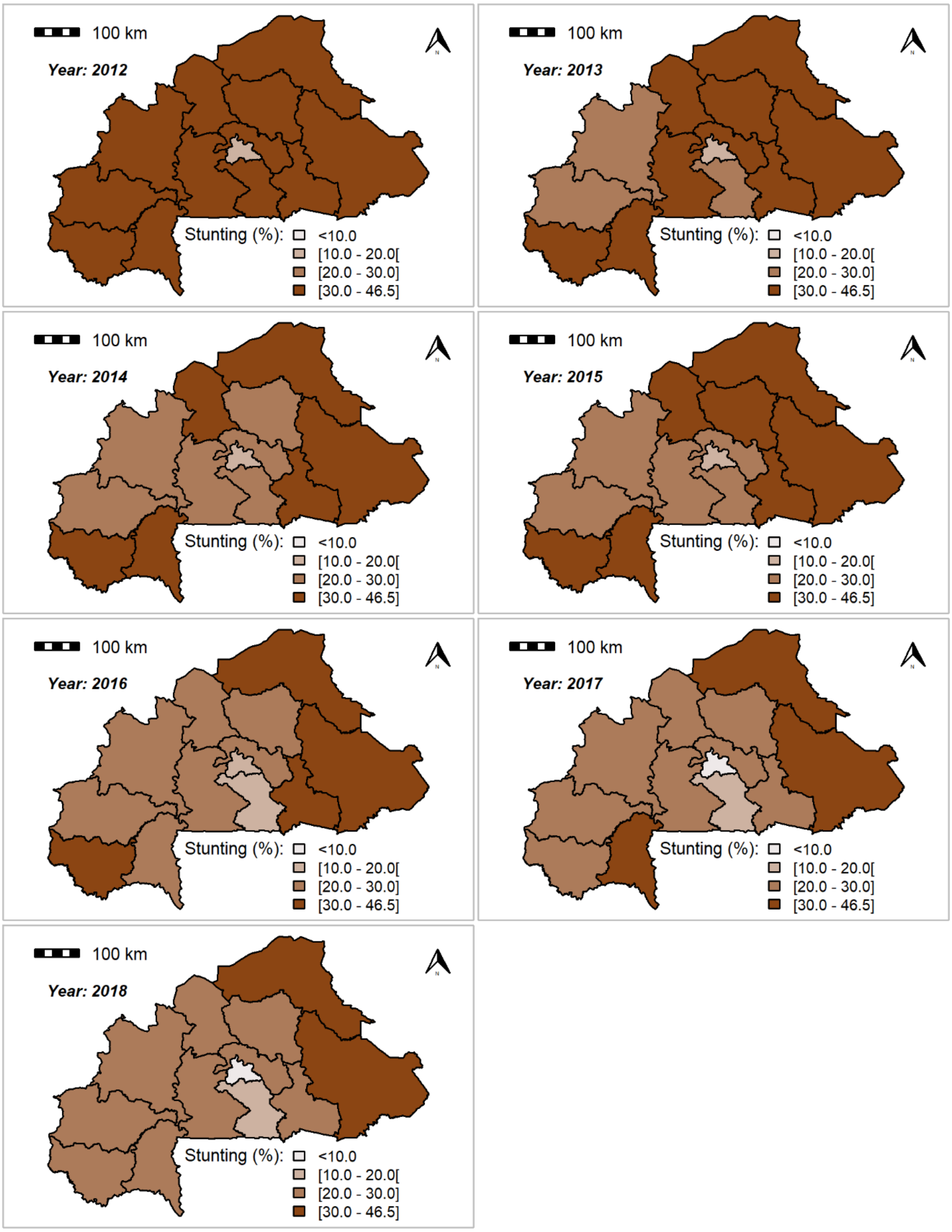

Figure 2. Maps of geographical distribution at regional level of stunting among children aged 0–59 months surveyed in Burkina Faso, 2012 to 2018.
Maps created by Rouamba T. et al., 2021.

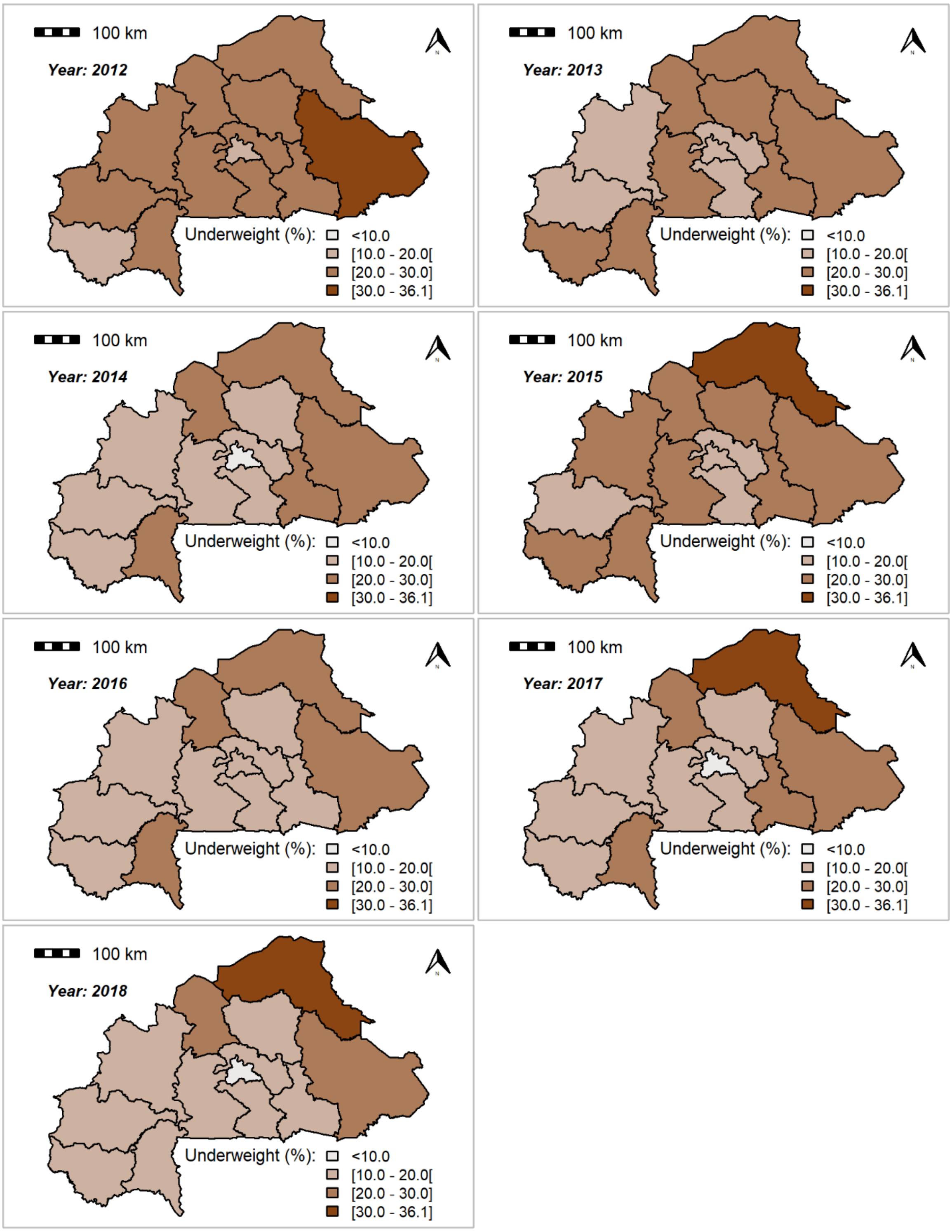

Figure 3. Maps of geographical distribution at regional level of underweight among children aged 0–59 months surveyed in Burkina Faso, 2012 to 2018.
Maps created by Rouamba T. et al., 2021.

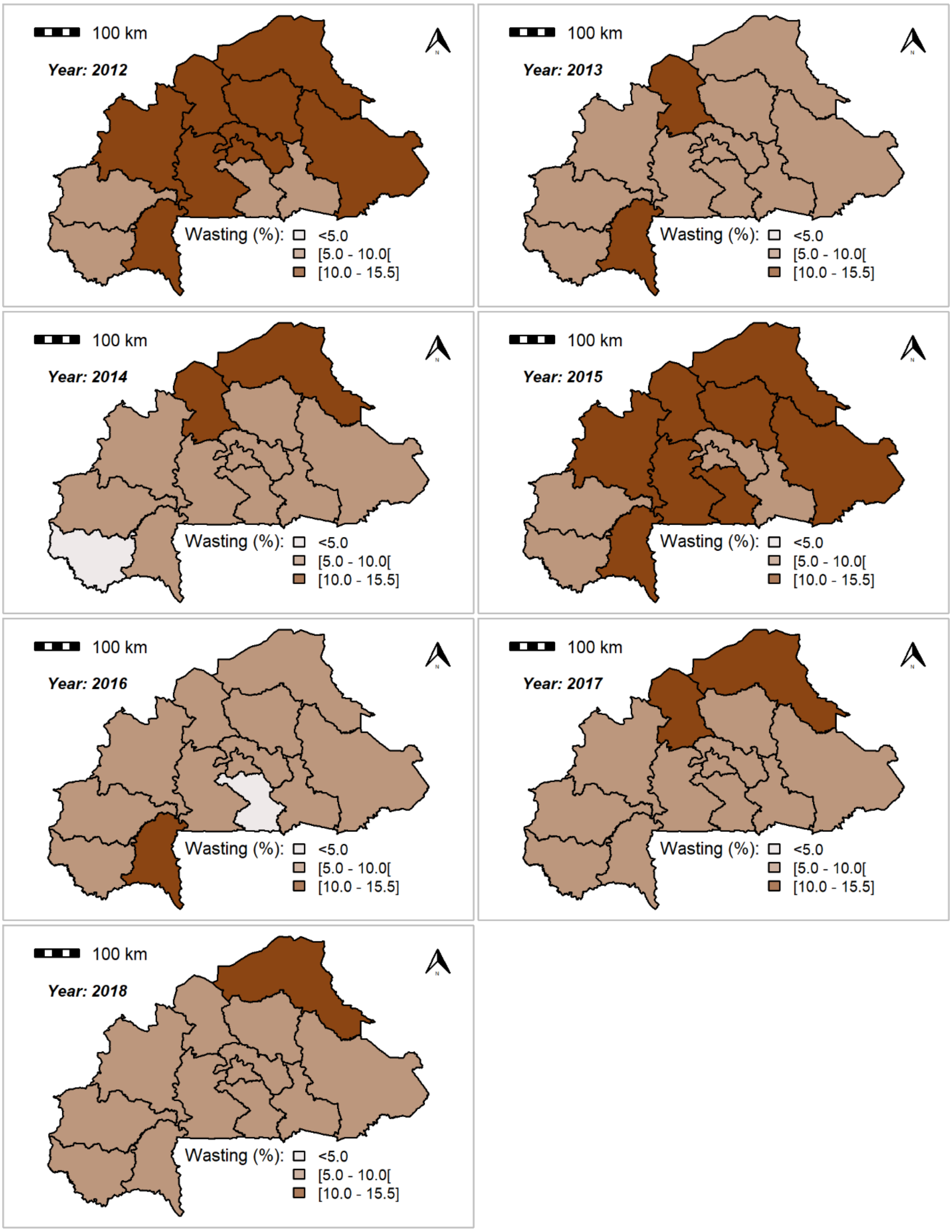

Figure 4. Maps of geographical distribution at regional level of wasting among children aged 0–59 months surveyed in Burkina Faso, 2012 to 2018.
Maps created by Rouamba T. et al., 2021.

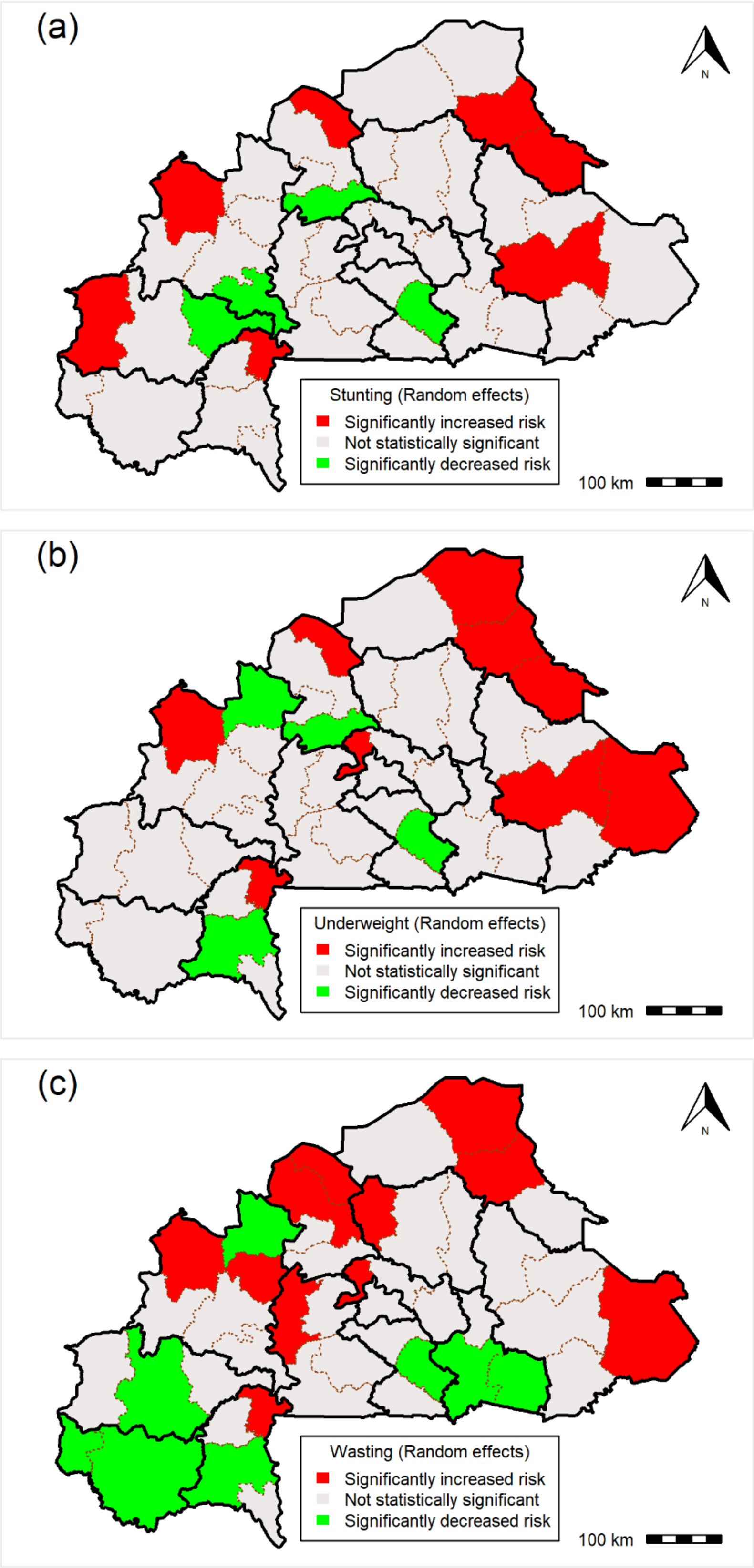

Figure 5. Overall province specific relative risk (province random effect) of stunting (a), underweight (b) and wasting (c) among study population.
Maps created by Rouamba T. et al., 2021.

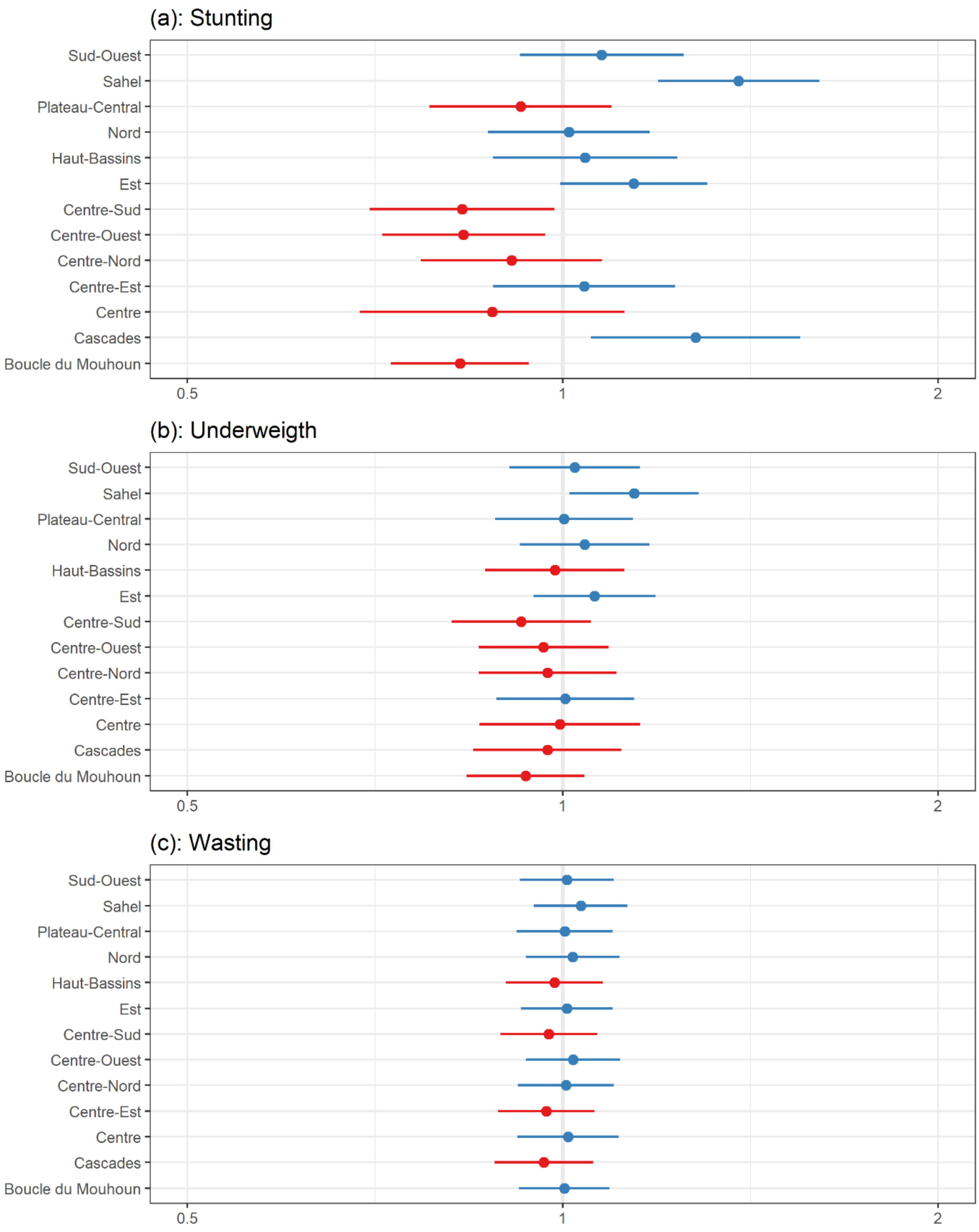

Figure 6. Overall region specific relative risk (region random effect) of stunting (a), underweight (b) and wasting (c) among study population.

Supplementary materials

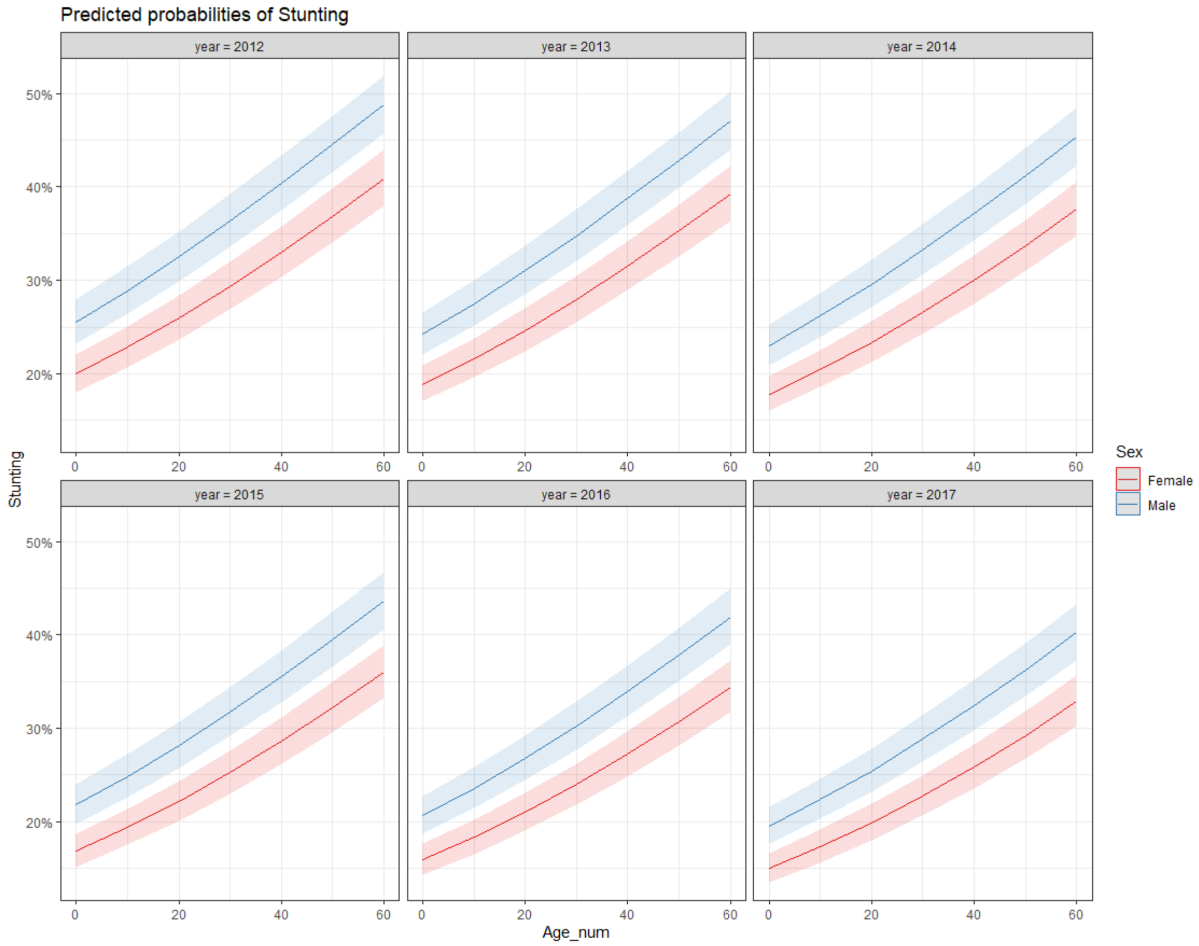

Figure S1. Predicted probabilities of stunting according to the child age and stratified by gender in Burkina Faso, from 2012 to 2017.

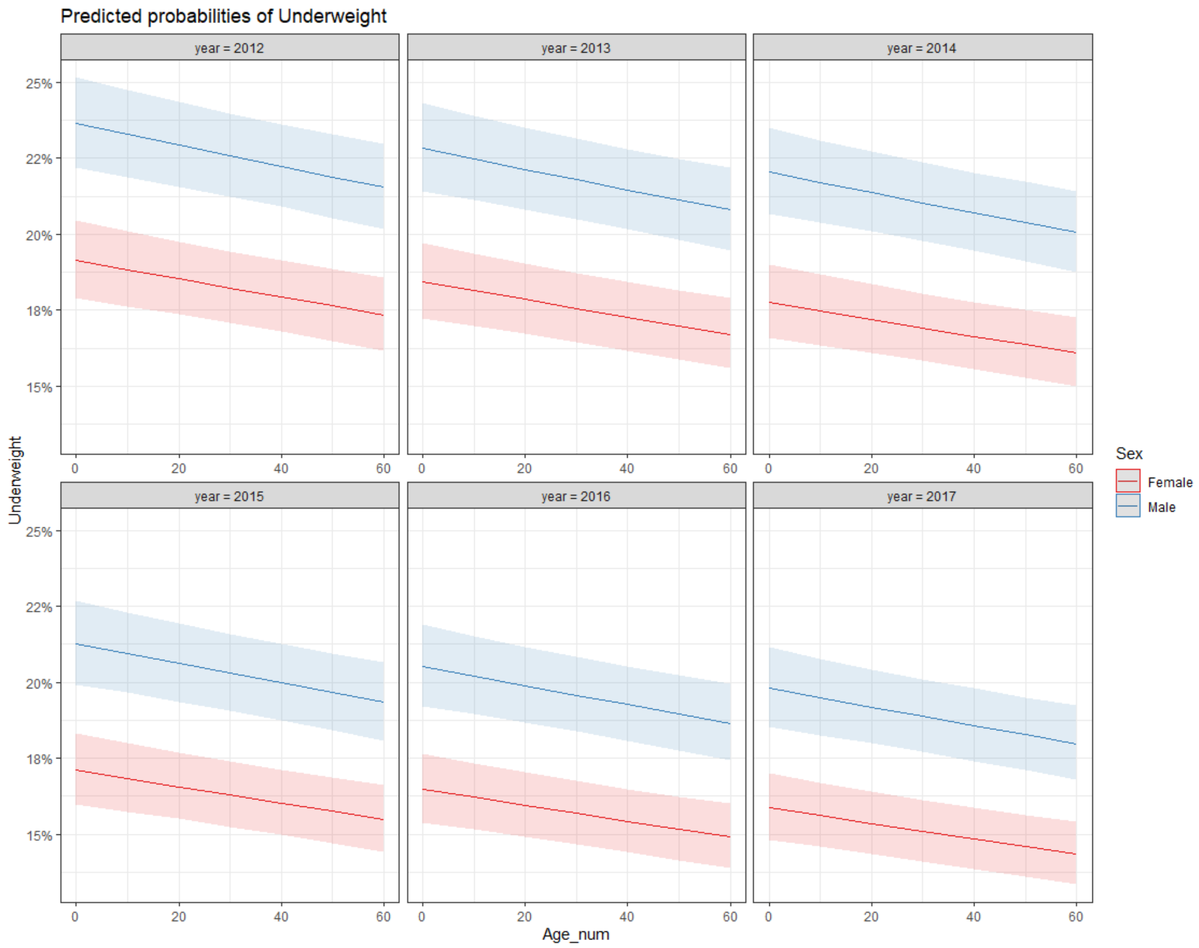

Figure S2. Predicted probabilities of underweight according to the child age and stratified by gender in Burkina Faso, from 2012 to 2017.

For peer review only

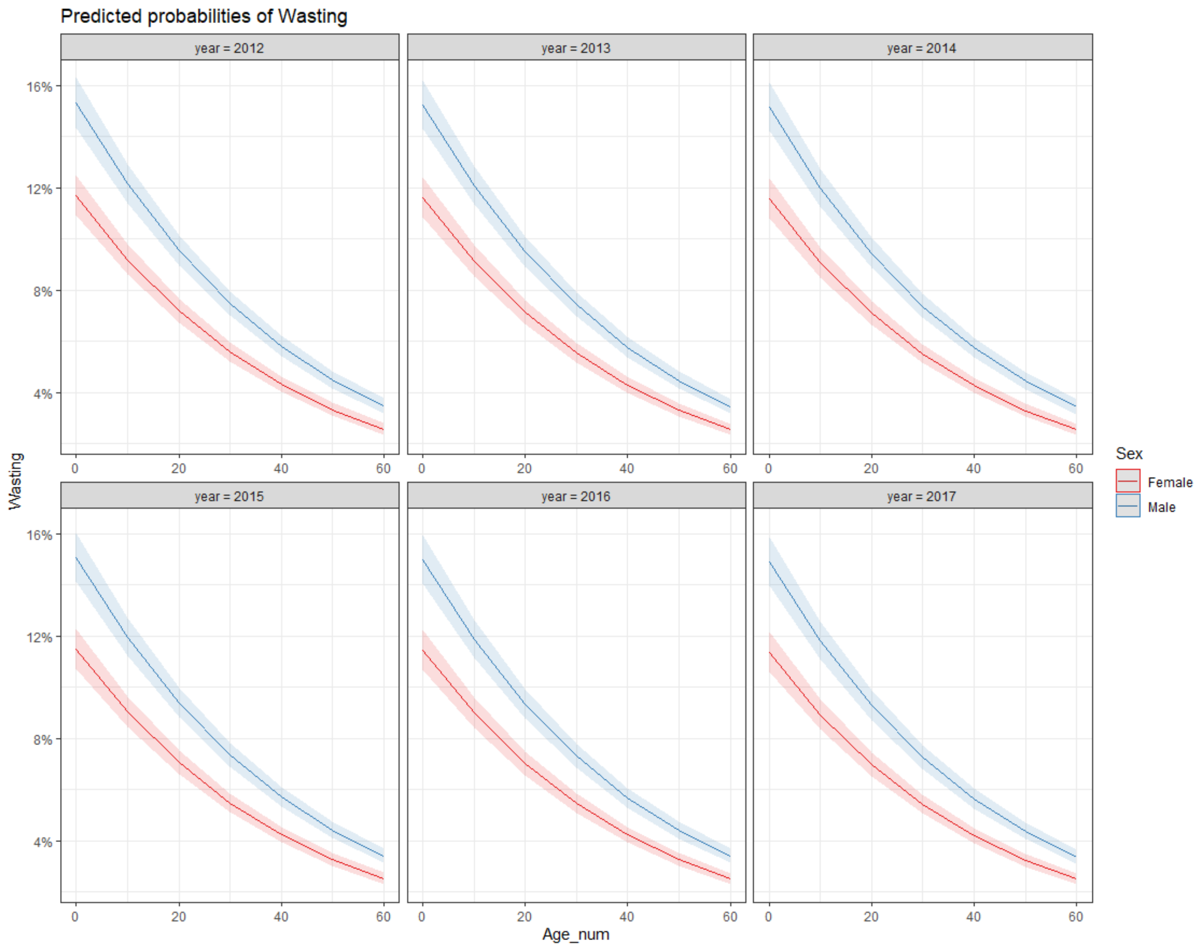

Figure S3. Predicted probabilities of wasting according to the child age and stratified by gender in Burkina Faso, from 2012 to 2017.

view only

BMJ Open

Undernutrition in children aged 0-59 months by region and over time : Secondary analysis of the Burkina Faso 2012-2018 National Nutrition Surveys.

Journal:	*BMJ Open*
Manuscript ID	bmjopen-2022-066509.R1
Article Type:	Original research
Date Submitted by the Author:	17-Apr-2023
Complete List of Authors:	Boua, Palwende; Institut de Recherche en Sciences de la Sante, Clinical Research Unit of Nanoro; University of the Witwatersrand Johannesburg, Sydney Brenner Institute for Molecular Biosciences<br>Rouamba , Toussaint; Institut de Recherche en Sciences de la Santé, Clinical Research Unit of Nanoro<br>Bambara, Estelle; Ministère de la Santé, Direction de la Nutrition<br>Kaboré, Saidou; Ministère de la Santé, Direction de la Nutrition<br>Compaore, Ella W. R.; Université Joseph Ki-Zerbo, Laboratoire de Biochimie, Biotechnologie, Technologie Alimentaire et Nutrition (LABIOTAN)<br>Ouedraogo, Boureima; Institut National de Statistique et de la Démographie<br>Tinto, Halidou; Institut de Recherche en Sciences de la Santé, Clinical Research Unit of Nanoro<br>Newell, Marie-Louise; University of Southampton, MRC Lifecourse Epidemiology Centre<br>Ward, Kate; University of Southampton, MRC Lifecourse Epidemiology Centre; University of Southampton, Global Health Research Institute<br>Sorgho, Hermann; Institut de Recherche en Sciences de la Santé, Clinical Research Unit of Nanoro
**Primary Subject Heading**:	Epidemiology
Secondary Subject Heading:	Nutrition and metabolism, Public health
Keywords:	EPIDEMIOLOGY, NUTRITION & DIETETICS, PUBLIC HEALTH

SCHOLARONE™
Manuscripts

I, the Submitting Author has the right to grant and does grant on behalf of all authors of the Work (as defined in the below author licence), an exclusive licence and/or a non-exclusive licence for contributions from authors who are: i) UK Crown employees; ii) where BMJ has agreed a CC-BY licence shall apply, and/or iii) in accordance with the terms applicable for US Federal Government officers or employees acting as part of their official duties; on a worldwide, perpetual, irrevocable, royalty-free basis to BMJ Publishing Group Ltd ("BMJ") its licensees and where the relevant Journal is co-owned by BMJ to the co-owners of the Journal, to publish the Work in this journal and any other BMJ products and to exploit all rights, as set out in our licence.

The Submitting Author accepts and understands that any supply made under these terms is made by BMJ to the Submitting Author unless you are acting as an employee on behalf of your employer or a postgraduate student of an affiliated institution which is paying any applicable article publishing charge ("APC") for Open Access articles. Where the Submitting Author wishes to make the Work available on an Open Access basis (and intends to pay the relevant APC), the terms of reuse of such Open Access shall be governed by a Creative Commons licence – details of these licences and which Creative Commons licence will apply to this Work are set out in our licence referred to above.

Other than as permitted in any relevant BMJ Author's Self Archiving Policies, I confirm this Work has not been accepted for publication elsewhere, is not being considered for publication elsewhere and does not duplicate material already published. I confirm all authors consent to publication of this Work and authorise the granting of this licence.

For peer review only

Title: Undernutrition in children aged 0-59 months by region and over time : Secondary analysis of the Burkina Faso 2012-2018 National Nutrition Surveys.

Palwende R. Boua[1,2Φ*], Toussaint Rouamba[1*], Estelle Bambara[3], Saidou Kaboré[3], Ella Compaoré[4], Boureima Ouedraogo[5], Halidou Tinto[1], Marie-Louise Newell[6], Kate Ward[6,7,8], Hermann Sorgho[1] on behalf of the INPreP Study Group

[1] Clinical Research Unit of Nanoro, Institut de Recherche en Sciences de la Santé, Burkina Faso

[2] Sydney Brenner Institute for Molecular Biosciences, University of the Witwatersrand, South Africa

[3] Direction de la Nutrition, Ministère de la Santé, Burkina Faso

[4] LABIOTAN, UFR/SVT, Université Joseph Ki-Zerbo, Ouagadougou, Burkina Faso

[5] Institut National de Statistique et de la Démographie, Ouagadougou, Burkina Faso

[6] MRC Lifecourse Epidemiology Centre, University of Southampton, United Kingdom

[7] Global Health Research Institute, School of Human Development and Health, Faculty of Medicine, University of Southampton, United Kingdom

[8] School of Public Health, Faculty of Health Sciences, University of the Witwatersrand, Johannesburg, South Africa

[Φ]Corresponding author: **Palwende Romuald Boua, PhD**
Clinical Research Unit of Nanoro, Institut de Recherche en Sciences de la Santé, Burkina Faso
218 CMS Ouaga 11
romyboua@gmail.com

*Equal contribution

Word count: 3094

For peer review only

Abstract

The global burden of undernutrition remains high, responsible for significant under-five mortality in resource-limited settings. Numerous sustainable development goals (SDGs) are linked to nutrition, and nationally representative nutrition surveillance is a key activity to track progress towards SDGs and guide efficient programs.

Objectives: The aim of this study is to look at spatial and temporal trends in undernutrition in children under five years age in Burkina Faso.

Setting: We used data from annual National Nutrition Surveys using SMART methodology (anthropometry, morbidity) over seven years (2012-2018) in Burkina Faso.

Participants: Children of under-five years from households selected through systemic sampling at countrywide level.

Main outcome measures: Prevalence of stunting (height-for-age, HAZ<-2), underweight (weight-for-age, WAZ<-2) and wasting (WHZ<-2) at regional and national. We used General Linear Mixed models, adjusted by age, survey year, sex, presence of fever and/or diarrhea, and poverty index to quantify risk of undernutrition over time and by region of residence.

Results: Between 2012 and 2018, decreases were observed overall in the prevalence of growth retardation (stunting) decreased from 33.0% (95%CI: 32.3 – 33.8) in 2012 to 26.7% (95%CI: 26.2 – 27.3) in 2018. Underweight reduced from 24.4% (95%CI: 23.7 – 25.1) to 18.7% (95%CI: 18.2 – 19.2) for the same period and wasting decreased from 10.8% (95%CI: 10.3 – 11.3) in 2012 to 8.4% (95%CI: 8.1 – 8.8) in 2018. However, there was substantial variation across the country, with increased risk of undernutrition in the regions of Sahel, East and Cascades mainly. High risk regions were characterized by a lower poverty index and limited access to healthcare services.

Conclusions: Our findings could inform national policymakers in refining and optimizing resource allocation based on identification of high-risk areas.

Key words: undernutrition, spatial, temporal, National Nutrional Suveys, Burkina Faso

For peer review only

Article Summary

Strengths and limitations of this study:

- Children morbidity is associated with all form of undernutrition.
- Poverty index is associated with chronic undernutrition but not with acute undernutrition.
- Under five boys are at higher risk of experiencing undernutrition compared to girls.
- Sahel region children are at higher risk of stunting and underweight.
- Our study did not cover all the National Nutrition Surveys from 2009 to 2021, due to data unavailability, therefore our finding is only relevant for the studied period. The analysis relies on survey data but not a cohort, hence causality cannot be inferred.

For peer review only

Background

Globally, undernutrition (eg wasting as a sign of acute undernutrition, and stunting as a sign of chronic undernutrition) remains an important public health issue, affecting the growth and mortality of children, especially in low- and middle-income countries [1]–[3]. Globally, in 2020, wasting and stunting affected an estimated 45.4 and 149.2 million children under 5 years of age, respectively [4]. [5], [6]. Undernoursihed children have a higher risk of death from common childhood illness such as diarrhoea, pneumonia, and malaria [5], [7], [8], and longer-term are likely to experience poorer physical and cognitive development, increased risk of chronic disease in older age, with undernourished women at increased risk of delivering low birth weight babies [9]–[11].

In Burkina Faso, like most low-income countries, improvements in nutrition though better diet quality and access to food, continues to be a major health, socioeconomic and political challenge, and is one of the main public health priorities towards achieving Sustainable Development Goals (SDGs) 1, 2, and 3 [12]. In Burkina Faso, more than one-third of deaths among children under five years of age are directly or indirectly attributable to undernutrition. Despite the development and implementation by successive governments of numerous policies and programs to deal with undernutrition, the prevalence of undernutrition (stunting, underweight and wasting) remains high. Factors such as recurrent drought, sociocultural practices (poor dietary practices), poverty, diarrheal and febrile diseases and insufficient access to basic health services negatively affect the nutritional status of children [3], [8], [13]–[15]**.** In addition, there is the problem of insecurity that the country has been experiencing since 2015, leading to massive internal displacement of the population, aggravating the already very limited access to basic livelihoods, health care and nutrition services in a context of poverty in these localities [16]–[18] [19], [20]. Food insecurity (lack of consistent access to enough food for every person in a household to live an active, healthy life) has been highly prevalent in the country (bulletin special FSC).

The government, supported by its financial and technical partners, has put in place a system to monitor the nutritional situation through the implementation of yearly national nutritional surveys according to the SMART (Standardized Monitoring and Assessment of Relief and Transitions) methodology. This methodology is widely used by governments and humanitarian partners to conduct timely nutrition surveys in all contexts (emergency, development, displaced populations). SMART surveys are conducted on a regular basis, often in connection with seasonal malnutrition, and can be conducted at the national or regional level, and even on a smaller scale. This paper follows a secondary analysis of the survey databases from 2012 to 2018 in order to have an overview of temporal trends and overall spatial risk of undernutrition and identify the areas to prioritize for resource allocation according to the risk of areas.

Methods

Study setting

Burkina Faso is a landlocked country in West Africa divided into 13 regions, 45 provinces, 351 communes (49 urban and 302 rural) and 8228 villages. According to the latest census, the country has 20.5 million inhabitants, 45.3% of whom are under 15 years old and children aged 0-4 years represent 16.2% of the total population [21]. The country is ranked 182nd out of 189 according to the Human Development Index report with more than 41.4% of its population living below the poverty line [22].

The country's epidemiological profile is marked by the persistence of a high burden of disease due to infectious disease, and by the progressive increase in the burden of non-communicable diseases. Beside the infectious and non-communicable diseases, undernutrition is one of the major diseases of public

For peer review only

health importance [23]. The food security situation is also of concern. It is estimated that approximately 3,28 million people are food insecure and in need of immediate humanitarian assistance, representing 15% of the country's total population [24].

Patient and public involvement

Because we used data from National Nutrition Surveys, it was not appropriate or possible to involve patients or the public in the design, or conduct, or reporting, or dissemination plans of our research.

Data sources

The Directorate of the Nutrition of Burkina Faso provided data (accessible through upon request). The data are from annual cross-sectional National Nutrition Surveys using the SMART methodology. This consists of a rapid assessment survey of the food and nutrition situation among children under 5 years of age, women of childbearing age and households. For this study, we focus on anthropometric measurements collected between 2012 and 2018 (seven surveys) among population aged 0-59 months.

We examined trends in three crude indices of nutritional problems in Burkina Faso over a seven-year period, namely stunting (height/length-for-age, L/HFA), underweight (weight-for-age, WFA) and wasting (weight-for-height/length, WFH/L). Anthropometric measurements were converted into weight-for-age z-score (WAZ), height-for-age z-score (HAZ), and weight-for-height z-score (WHZ), using the 2006 World Health Organization (WHO) child growth standards [25]. Children with oedema, which can be a sign of acute undernutrition) were excluded as recommended for this analysis (**Table 1**).

Table 1. Definition of anthropometric measurements and indices in children under five

Index	Nutritional problem measured	Indicator
Height/Length-for-age (H/LFA)	Severe stunting	HAZ < −3 SD
	Moderate stunting	HAZ < −2 SD and HAZ ≥ −3 SD
	Global stunting	HAZ < −2 SD
Weight-for-age (WFA)	Severe underweight	WAZ < −3 SD
	Moderate underweight	WAZ < −2 SD and WAZ ≥ −3 SD
	Global underweight	WAZ < −2 SD
Weight-for-Height/Length (WFH/L)	Severe wasting	WHZ < −3 SD
	Moderate wasting	WHZ < −2 SD and WHZ ≥ −3 SD
	Global wasting	WHZ < −2 SD

HAZ: Length/Height-for-age z-score; WAZ: Weight-for-age z-score; WHZ: Weight-for-length/height z-score

For 2012 and 2014, the SMART survey was provincially representative for six regions (Cascades, Centre West, East, North, Sahel and South West) and regionally representative for the other seven (Boucle du Mouhoun, Centre-South, Centre, Centre-North, Centre-East, Central Plateau and Hauts-Bassins). For 2013 and 2015, the SMART survey had provincial representativeness for seven regions (Boucle du Mouhoun, Centre-South, Centre, Centre-North, Centre-East, Central Plateau and Hauts-Bassins) and regional representativeness for the other six (Cascades, Centre-West, East, North, Sahel and South-

West). For 2016, the SMART survey had regional representativeness for 13 regions (resulting in smaller sample) and from 2017 onwards, the SMART survey had provincial representativeness for the 45 provinces across the 13 regions.

Each level of representativeness constituted strata.

Outcome variables

Stunting was determined if HAZ < −2 SD, underweight as WAZ < −2 SD and wasting as WHZ < −2 SD (Table 1). We excluded z-scores with extreme values from the observed mean, the following WHO indicators were used in the final analysis: WHZ - 5 to 5; HAZ - 6 to 6; WAZ - 6 to 5.

The independent variables were individual-level characteristics mainly age in months and gender, diarrhoea and fever during the 2 weeks preceding the survey, year of the survey and cluster-level characteristics constituted by the poverty index for each province [26].

Statistical analysis

For descriptive statistics at the individual and strata levels, estimates account for the complex survey design and the sampling weights provided by *survey*. Descriptive analysis of the study population was presented using means and standard error for continuous variables and frequencies and proportions for categorical variables. These descriptive analyses were performed to estimate the crude temporal trend of prevalence nationwide and provide a yearly map for the regional disparities of undernutrition index.

Considering that the data have a hierarchical structure, and we would need to account for the cluster (province and region) variability as well as the individual-to-individual variability, we used General linear mixed models (multilevel models). GLMM (multilevel models) with binomial distribution were used to quantify the association of independent variables with each study outcome variable (stunting, underweight and wasting). We assume that the outcomes variable were drawn from a binomial distribution and the residual are independent and normally distributed. The general linear mixed model contains fixed and random effects and was formulated as follow:

$\boldsymbol{Y_{ij}} \sim Bernoulli\ (\pi_{ij})$

$$logit\ (\pi_{ij}) = log\left[\frac{\pi_{ij}}{1-\pi_{ij}}\right] = \beta_0 + \sum_{p=1}^{P} \beta_p x_{ij} + \sum_{q=1}^{Q} \beta_q z_j + \omega_j + \varepsilon_i$$

ε_i~N(0,1)

The random effects are also, independent and normally distributed.

In this general linear model, $\boldsymbol{Y_{ij}}$ represent stunting, underweight or wasting for a child i in a cluster j. The logit (π_{ij}) was used to model the probability of success (i.e., occurrence of undernutrition) as a linear combination of observed individual characteristics (x_{ij}) and contextual characteristics (z_j) associated with an unobserved specific effect (random effect) of the province and region (ω_j). The model contains a random error for child *i* (ε_i) that does not depend to the province/region nor year. The fixed effect is composed of $\boldsymbol{\beta_0}$ (intercept), $\boldsymbol{\beta_p}$ (vector of regression coefficients associated with the individual-level variables) and $\boldsymbol{\beta_q}$ (vector of regression coefficients associated with the cluster-level variables). The unobserved specific effect or random effect $(\boldsymbol{\omega_j})$ has two components, province specific effect and region specific effect. These cluster specific effects were considered as province and region specific risk.

Since we intended to quantify the association between the outcomes and dependent variables by avoiding that the coefficient be affected by the individual weight, survey weights were not used to adjust the regressions and a complete case analysis was used. The model diagnostics was check by

For peer review only

generating a diagnostic that plots the fitted or predicted values against the residuals. All statistical analyses were performed with R statistical software (R Development Core Team, R Foundation for Statistical Computing, Vienna, Austria). The descriptive statistics that accounted for the complex survey design and sampling weights were performed using "survey" package, regression models were fitted using the "lme" package.

Results

Demographic characteristics and anthropometrical measures of children

The overall number of children under five assessed for their nutritional status using anthropometric measurement and included in the present analysis is showed in the Table 2. Since the survey in 2016 was set up to have regionally representative estimates, 9434 children were surveyed. Gender was equally distributed through the surveys and varied between 51.6% and 51.1% for the proportion of male children. The average age of the children during the period of 2012 to 2018 ranged from 27 to 29 months.

The distribution of anthropometric measures such as weight, height and the z-scores with WHO as reference population (HAZ, WAZ and WHZ) from 2012 to 2018 are presented in the Table 2 below. All z-scores had a mean value below the WHO standard. HAZ values ranged from -1.50 to -1.27; WHZ values ranged from -0.70 to -0.53; WAZ values ranged from -1.33 to -1.11.

Table 2. Characteristic of Children under five Surveyed at household in Burkina Faso, 2012 to 2018

	Weighted Mean (standard error)						
Index	**2012** N= 19049	**2013** N= 19170	**2014** N= 15835	**2015** N= 20408	**2016*** N= 9434	**2017** N= 19409	**2018** N= 23939
Age	27.80 (0.11)	27.15 (0.12)	27.82 (0.13)	27.47 (0.11)	28.59 (0.16)	28.53 (0.11)	29.04 (0.10)
Weight	10.46 (0.02)	10.39 (0.02)	10.72 (0.03)	10.41 (0.02)	10.85 (0.03)	10.8 (0.02)	10.92 (0.02)
Height	82.08 (0.09)	81.54 (0.1)	82.68 (0.11)	81.97 (0.09)	83.38 (0.13)	83.31 (0.09)	83.75 (0.08)
HAZ	-1.49 (0.01)	-1.50 (0.01)	-1.30 (0.01)	-1.47 (0.01)	-1.30 (0.01)	-1.28 (0.01)	-1.27 (0.01)
WHZ	-0.67 (0.01)	-0.61 (0.01)	-0.53 (0.01)	-0.70 (0.01)	-0.55 (0.01)	-0.60 (0.01)	-0.56 (0.01)
WAZ	-1.31 (0.01)	-1.28 (0.01)	-1.11 (0.01)	-1.33 (0.01)	-1.12 (0.01)	-1.15 (0.01)	-1.11 (0.01)

* In 2016 the sampling framework was regional and national (without provincial), hence impacting the sample size. This choice by the Ministry of Health was guided by limited resources to run the national survey.

Prevalence of undernutrition

The annual prevalence of chronic undernutrition (stunting) using HAZ ranged from 33.0% (95%CI: 32.3 – 33.8) in 2012 to 26.7% (95%CI: 26.2 – 27.3) in 2018, with a maximum of 34.2% (95%CI: 33.4 – 34.9) in 2013. In other words, between 2013 and 2018, stunting prevalence declined among children under five from 33.4% to 26.7%, a decline of 19.1% (Supplemental Figure 1 (a)).

The annual prevalence of underweight (WAZ) ranged from 24.4% (95%CI: 23.7 – 25.1) in 2012 to 18.7% (95%CI: 18.2 – 19.2) in 2018, with a maximum of 25.3% (95%CI: 24.6 - 25.9) in 2015 (Supplemental Figure 1 (b)). This meant that nationally, underweight decreased by 23.3% between 2012 and 2018.

The annual prevalence of wasting (WHZ) (Supplemental Figure 1 (c)) ranged from 10.8% (95%CI: 10.3 – 11.3) in 2012 to 8.4% (95%CI: 8.1 – 8.8) in 2018, with a maximum of 11.7% (95%CI: 10.7 – 11.6) in 2015. In other words, between 2013 and 2018, global wasting prevalence declined among children under five from 33.4% to 26.7%, corresponding to a decline of 22.2%.

Overall, there was a reduction in prevalence of undernutrition over the years, but this was not seen in all areas. In other words, there was a spatial heterogeneity of undernutrition index through the country.

For peer review only - http://bmjopen.bmj.com/site/about/guidelines.xhtml

During the seven years, the analysis showed a permanent high prevalence (>30%) of stunting in Sahel, and East regions (Supplemental **Figure 2**), whereas the stable high prevalence of underweight (>30%) was mainly noted in the Sahel region (Supplemental **Figure 3**). The high prevalence of wasting was mainly noted in the Sahel and North region (Supplemental **Figure 4**).

The predicted probabilities of stunting, underweight, wasting by child's age at survey, and stratified by gender for the year 2012 to 2017 are shown in Supplemental Figures 5, 6 and 7. The probability of being stunted increased with age, and was higher for male than female children, but decreased over time, i.e. between 2012 and 2018 (Supplemental **Figure 5**). Similarly, predicted prevalence of underweight decreased with children age, was greater among male children and declined over the study period i.e. from 2012 to 2018 (Supplemental **Figure 6**). For wasting, the probability decreased with child's age and was more marked among males, but the trend over time was stable (Supplemental **Figure 7**).

Potential risk factors associated with Stunting, Underweight, and Wasting from 2012 to 2017 in Burkina Faso

In multivariable analysis, allowing for adjustment (Table 3) males had a significantly increased risk of stunting (aOR=1.47, 95%CI: 1.43 – 1.52), underweight (aOR=1.39, 95%CI: 1.34 – 1.43) and wasting (aOR=1.38, 95%CI: 1.31 – 1.44) than females. Compared with children aged less than 5 months, older children had higher risks of stunting and underweight, but the odds of wasting decreased significantly over the age of 24 months. Compared with the year 2012, the analysis showed that the risk of stunting, underweight and wasting in the subsequent years, i.e. 2013 to 2018, decreased significantly overall, except for the year 2015 for wasting. Furthermore, diarrhoea was associated with high odds of stunting (aOR=1.08, 95%CI: 1.05 – 1.12), underweight (aOR=1.32, 95%CI: 1.27 – 1.37) and wasting (aOR=1.46, 95%CI: 1.39 – 1.54). This association was similar with the fever. There was no significant association between wasting and poverty index (aOR=1.03, 95%CI: 0.96 – 1.10), whereas stunting and underweight were significantly associated with the poverty index. The model diagnostics plots were presented in supplementary materials (Text and Supplemental Figure 8, 9 and 10).

Table 3. Potential risk factors associated with Stunting, Underweight, and Wasting from 2012 to 2017 in Burkina Faso

	Stunting		Underweight		Wasting	
Predictors	aOR (95%CI)	p	aOR (95%CI)	p	aOR (95%CI)	p
Gender						
Female	1		1		1	
Male	1.39 (1.35 – 1.43)	<0.001	1.31 (.27 – 1.35)	<0.001	1.37 (1.31 – 1.43)	<0.001
Age in Month						
00 - 05	1		1		1	
06 - 11	1.51 (1.39 – 1.63)	<0.001	1.96 (1.82 – 2.11)	<0.001	1.67 (1.53 – 1.82)	<0.001
12 - 23	3.74 (3.49 – 4.00)	<0.001	2.56 (2.39 – 2.73)	<0.001	1.53 (1.41 – 1.65)	<0.001
24 - 35	4.94 (4.62 – 5.28)	<0.001	2.28 (.13 – 2.44)	<0.001	0.83 (0.76 – 0.90)	<0.001
36 - 47	4.57 (4.27 – 4.89)	<0.001	1.67 (1.56 – 1.79)	<0.001	0.46 (0.41 – 0.50)	<0.001
48 - 59	3.44 (3.20 – 3.68)	<0.001	1.58 (1.47 – 1.69)	<0.001	0.49 (0.44 – 0.54)	<0.001
Year of survey						
2012	1		1		1	
2013	0.89 (0.85 – 0.94)	<0.001	0.79 (0.75 – 0.84)	<0.001	0.72 (0.66 – 0.78)	<0.001
2014	0.78 (0.75 – 0.82)	<0.001	0.72 (0.69 – 0.76)	<0.001	0.73 (0.68 – 0.79)	<0.001
2015	0.87 (0.82 – 0.91)	<0.001	0.95 (0.90 – 1.00)	0.063	1.00 (0.92 – 1.08)	0.902
2016	0.73 (0.69 – 0.78)	<0.001	0.74 (0.69 – 0.79)	<0.001	0.70 (0.63 – 0.77)	<0.001
2017	0.67 (0.64 – 0.71)	<0.001	0.71 (0.68 – 0.75)	<0.001	0.86 (0.80 – 0.93)	<0.001
Diarrhea						
No	1		1		1	
Yes	1.19 (1.14 – 1.23)	<0.001	1.36 (1.31 – 1.42)	<0.001	1.39 (1.32 – 1.47)	<0.001
Fever						

For peer review only

No	1		1		1	
Yes	1.08 (1.05 – 1.12)	<0.001	1.31 (1.27 – 1.35)	<0.001	1.48 (1.41 – 1.55)	<0.001
Poverty Index in %	1.17 (1.09 – 1.26)	<0.001	1.15 (1.08 – 1.22)	<0.001	1.03 (0.96 – 1.10)	0.456
**Random Effects**						
σ2	3.29		3.29		3.29	
τ00						
Provinces	0.03		0.03		0.04	
Region	0.03		0.01		0.00	
ICC	0.02		0.01		0.01	
N Region	13		13		13	
N Provinces	45		45		45	
Observations	83161		83161		83161	

Estimation of province and region-specific risk

The province spatial risk of stunting, underweight and wasting was shown to be heterogeneous throughout the country. Figure 5 displays the specific risks (or odds) of each province compared with the national mean odds ratio (aOR = 1).

For stunting, the provinces with high odds were located in eight provinces namely Gourma, Ioba, Kenedougou, Kossi, Lorum, Seno and Yagha provinces, whereas the provinces with lower odds were located in four provinces namely, Bales, Passore, Tuy, and Zounweogo provinces (**Figure 1 (a)**).

Regarding underweight, the provinces with high odds were located in nine provinces namely Gourma, Ioba, Kossi, Kourweogo, Lorum, Oudalan, Seno, Tapoa, Yagha provinces, whereas the provinces with lower odds were located in four provinces namely, Passore, Poni, Sourou and Zounweogo provinces (**Figure 1 (b)**).

Regarding wasting, the provinces with high odds were located in 11 provinces namely, Bam, Ioba, Kossi, Kourweogo, Lorum, Nayala, Oudalan, Sanguie, Seno, Tapoa and Yatenga provinces whereas, the provinces with lower odds were located in eight provinces namely, Boulgou, Comoe, Houet, Koulpelgo, Leraba, Poni, Sourou, and Zounweogo province (**Figure 1 (c)**).

Further, the analysis also indicated some regions where the odds was statistically significant higher (Sahel and Cascades) or lower (Centre-sud, centre-Ouest and Boucle de Bouhoun) for stunting (**Figure 2 (a)**). For underweight, only the region of the Sahel exhibited higher odds (**Figure 2 (b)**). Finally, regarding wasting, no region exhibited a higher or lower risk (**Figure 2 (c)**).

Discussion

This study undertook a secondary analysis using data from annual cross-sectional National Nutrition Survey (NNS) using SMART methodology to generate estimates that could help national policymakers in refining and optimizing resource allocation based on identification of high-risk areas. The analysis found that undernutrition remains very high in certain part of the country, although there was an overall decrease in prevalence and risk over the years in the country; the geospatial risk to experience an undernutrition event is not uniform throughout the country. This decrease of all undernutrition prevalence has been observed in other sub-Saharan Africa countries despite predicted stagnation in the region for stunting until 2020 [27]–[29].

In previous analysis, De Onis and collaborators predicted that in Africa, the prevalence of stunting was going to stagnate in next coming decade from 2010, and specifically in Western Africa [29]. Our results showed a decrease of stunting prevalence of 6.7 between 2012 and 2018. De Onis prediction were based on poor data from some countries with lag time of about ten years between them (namely Demographic

For peer review only

and Health Survey), and the landscape of interventions in nutrition was poor. From 2009, a nutrition surveillance system has been implemented with yearly national surveys, additionally more effective nutrition programs to improve nutritional status across the whole population have been implemented.

Sex of child (male), two weeks morbidity (fever, diarrhea) were factors associated with the three forms of undernutrition; whereas poverty measured through wealth index was associated with stunting and underweight but not wasting. In their analysis of Nigeria Demographic and Health Survey (NDHS), Akombi et al. found the same factors associated to stunting[30]. Biological and social mechanism or both has been proposed to explain the differences between boys and girls [31]. Even though two weeks morbidity might not be causal to chronic undernutrition, it translates the effect of morbidity and or repeated morbidity on growth retardation and underweight.

Living in certain provinces and/or regions of the country is conferring higher risk of suffering from undernutrition. Similar findings were reported in Côte d'Ivoire, Uganda and Nigeria [28], [30], [32]. In Burkina Faso, these areas were the one with the highest proportion of the population living more than 10 Km away from the primary health center (first level of referral) [23]. This means that access to health care could be associated with nutritional status. The persistent prevalence of undernutrition translate a need, not just for the community management of wasting programs which have been shown to be effective, but for effective prevention programs as well as structural changes allowing reduction of inequalities [33]–[36].

Sahel region had the highest risk of stunting and underweight. Prior to the recent threats from terrorism, Burkina Faso had already endured decade of a fragile food security situation due to droughts and floods, affecting food security and nutrition, especially in the north part of the country. Restrictions on the movement of people and goods, as well as limited access to natural resources and regional or international markets, had placed a heavy burden on local food systems and people's livelihoods. The food insecurity situation in Burkina Faso is currently aggravated by a high level of insecurity. The populations of the Sahel zone are the most affected. Undernutrition is chronic and its causes are structural and complex. They are the result of a combination of several factors, including a short agricultural production season lasting four months of the year, climatic vulnerability, the depletion of natural resources, a chronic shortage of agricultural production, and very little access to basic social services due to the weak state presence.

Insecurity marked by attacks from terrorist groups and the increase in inter-community conflicts exacerbate food and nutrition insecurity in the Sahel and North Central regions by reducing the already fragile livelihoods of populations that depend mainly on agriculture and pastoralism. Insecurity limits access to fields and agricultural production. The resulting road closures affect the supply of agricultural products to markets, reduce the availability of agricultural products, limit access due to price fluctuations, and hinder transhumance and access of animals to pasture within Burkina and to Mali and Niger. The looting of food reserves and livestock reduces the resilience of communities affected by this insecurity. In addition, many health facilities are closed or operating at minimal capacity in areas affected by insecurity, reducing access to health services, including those for severely malnourished children. This has forced people to move to other parts of the country (almost one million internally displaced persons (IDPs) according to the United Nations Office for the Coordination of Humanitarian Affairs), resulting in overcrowding in host towns. While some IDPs reside in camps, more than 80% of IDPs are staying with host families whose food supplies are rapidly running out.

In our study, we found that poverty index is not associated with wasting, that mean acute undernutrition is due to an underlying disease causing abnormal nutrient loss, increased energy expenditure, or decreased food intake. Based on its etiology, undernutrition is either illness related (one or more diseases or injuries directly result in nutrient imbalance) or caused by environmental/behavioral factors associated with decreased nutrient intake and/or delivery [11], [37],

For peer review only - http://bmjopen.bmj.com/site/about/guidelines.xhtml

[38]. Therefore, primary acute undernutrition is mostly social rather than biomedical in origin, but it is also multifactorial. For example, poor water quality, sanitation and hygiene practices are increasingly believed to be the cause of the condition called "environmental enteropathy" that contributes to acute undernutrition in childhood [38].

Although this study provided insight on the temporal trends and overall spatial risk in undernutrition among children under five between 2012 and 2018 in Burkina Faso, this study is influenced by several limitations. Our study did not cover all the National Nutrition Surveys from 2009 to 2021, due to data unavailability, therefore our finding is only relevant for the studied period. The analysis relies on survey data but not a cohort, hence causality cannot be inferred.

Conclusion

The study found that undernutrition prevalence remains high and marked spatial disparities. Overall, there is a decreased of prevalence over the year. It is important to note that we found low risk area where the control effort need to maintained and reinforced. The high-risk area, need to be allocated more resources and reinforce the control effort. Morbidity (fever and diarrhea) constitutes important associated factors and need prompt and effective management with particular focus on children over 6 months. Further research should include birth cohort to allow causality inference for undernutrition in Burkina Faso. Additionally, surveillance system should be implemented towards IDPs. Our study showed that secondary analysis of national surveillance data has the potential to inform public health action and refinement of intervention.

For peer review only

Acknowledgements

The authors would like to thank the Ministry of Health and Public Hygiene for allowing access to the data.

Funding

This research was funded by the National Institute for Health Research (NIHR) (17/63/154) using UK aid from the UK Government to support global health research. The views expressed in this publication are those of the author(s) and not necessarily those of the NIHR or the UK Department of Health and Social Care.

Conflict of interest

The authors declare that they have no competing interests.

Authorship

H.S., M.L.N., P.R.B., H.T. and K.W. designed the WP5 study. H.S., P.R.B., H.T. and K.W. supervised the implementation of the study and guided the manuscript conception. P.R.B. and TR analysed the data, carried out the literature review and drafted the paper. E.B, S.K, E.C, B.O reviewed and approved the final manuscript.

Ethics

This study is part of the of the INPreP3 Study and has been approved by the National Health Ethics Committee in Burkina Faso, Approval number 2018-12-156.

Data sharing

Access to data is available upon request to the Ministry of Health and Public Hygiene.

References

[1] FAO, IFAD, UNICEF, and WFP, "The State of Food Security and Nutrition in the World 2021. Transforming food systems for food security, improved nutrition and affordable healthy diets for all," Rome, 2021.

[2] Development Initiatives, "The burden of malnutrition - Global Nutrition Report," Bristol, 2018.

[3] M. Myatt *et al.*, "Children who are both wasted and stunted are also underweight and have a high risk of death: A descriptive epidemiology of multiple anthropometric deficits using data from 51 countries," *Arch. Public Heal.*, vol. 76, no. 1, Jul. 2018.

[4] UNICEF / WHO / World Bank Group, "UNICEF-WHO-The World Bank: Joint child malnutrition estimates – levels and trends – 2021 edition - UNICEF DATA," 2021.

[5] "Children: improving survival and well-being." .

[6] D. L. Pelletier, E. A. Frongillo, and J. P. Habicht, "Epidemiologic evidence for a potentiating effect of malnutrition on child mortality," *Am. J. Public Health*, vol. 83, no. 8, pp. 1130–1133, 1993.

[7] R. E. Black *et al.*, "Maternal and child undernutrition: global and regional exposures and health consequences," *Lancet*, vol. 371, no. 9608, pp. 243–260, 2008.

[8] R. E. Black *et al.*, "Maternal and child undernutrition and overweight in low-income and middle-income countries," *Lancet*, vol. 382, no. 9890, pp. 427–451, 2013.

For peer review only

[9] J. McFie and H. F. Welbourn, "Effect of Malnutrition in Infancy on the Development of Bone, Muscle and Fat," *J. Nutr.*, vol. 76, no. 2, pp. 97–105, Feb. 1962.
[10] H. Darsene, A. Geleto, A. Gebeyehu, and S. Meseret, "Magnitude and predictors of undernutrition among children aged six to fifty nine months in Ethiopia: A cross sectional study," *Arch. Public Heal.*, vol. 75, no. 1, Jul. 2017.
[11] L. Shahrin, M. J. Chisti, and T. Ahmed, "3.1 Primary and secondary malnutrition," *World Rev. Nutr. Diet.*, vol. 113, pp. 139–146, 2015.
[12] "United Nations Sustainable Development Goals (SDGs)." .
[13] C. M. McDonald *et al.*, "The effect of multiple anthropometric deficits on child mortality: Meta-analysis of individual data in 10 prospective studies from developing countries," *Am. J. Clin. Nutr.*, vol. 97, no. 4, pp. 896–901, Apr. 2013.
[14] T. Khara, M. Mwangome, M. Ngari, and C. Dolan, "Children concurrently wasted and stunted: A meta-analysis of prevalence data of children 6–59 months from 84 countries," *Matern. Child Nutr.*, vol. 14, no. 2, Apr. 2018.
[15] M. Garenne *et al.*, "Incidence and duration of severe wasting in two African populations," *Public Health Nutr.*, vol. 12, no. 11, pp. 1974–1982, Feb. 2009.
[16] Davycas, UNICEF, WFP, and Ministry of Health, "Nutrition survey in communities and sites with internally displaced persons (IDPs) in Burkina Faso," Ouagadougou, 2020.
[17] V. Ridde, L. Lechat, and I. B. Meda, "Terrorist attack of 15 January 2016 in Ouagadougou: how resilient was Burkina Faso's health system?," *BMJ Glob. Heal.*, vol. 1, no. 1, p. e000056, 2016.
[18] International Crisis Group, "The Social Roots of Jihadist Violence in Burkina Faso's North | Crisis Group," *Africa Report N°254*, 2017. .
[19] Save the Children, DS/DRS de Kaya, and Lionella Fieschi, "Prise en charge Communautaire de la Malnutrition Aiguë (PCMA): Évaluation Semi-Quantitative de l'Accessibilité et de la Couverture (SQUEAC)," Kaya, Burkina Faso, 2010.
[20] Ministry of Health, "Guide pour le suivi et la gestion de la prise en charge de la malnutrition aiguë," Ouagadougou, Burkina Faso, 2009.
[21] Institut National de la statitistique et de la démographie, "Cinquième Recensement Général de la Population et de l'Habitation du Burkina Faso," no. août, p. 400, 2022.
[22] The World Factbook, "The World Factbook — Central Intelligence Agency. Burkina Faso population projection." .
[23] Ministère de la Santé Burkina Faso (DGESS), "Annuaire statistique 2020," Ouagadougou, Apr. 2020.
[24] Ministère de la Santé, "Enquête Nutritionnelle Nationale (SMART-2020)," Ouagadougou, Burkina Faso, 2020.
[25] World Health Organization, "Length/height-for-age, weight-for-age, weight-for-length, weight-for-height and body mass index-for-age Methods and development," 2006.
[26] Y. Zida and S. H. Kambou, "Cartographie de la pauvreté et des inégalités au Burkina Faso," 2014.
[27] P. K. Masibo and D. Makoka, "Trends and determinants of undernutrition among young Kenyan children: Kenya Demographic and Health Survey; 1993, 1998, 2003 and 2008-2009.," *Public Health Nutr.*, vol. 15, no. 9, pp. 1715–1727, 2012.
[28] E. Barankanira, N. Molinari, P. Msellati, C. Laurent, and K. A. Bork, "Stunting among children under 3 years of age in Côte d'Ivoire: Spatial and temporal variations between 1994 and 2011," *Public Health Nutr.*, vol. 20, no. 9, pp. 1627–1639, 2017.
[29] M. De Onis, M. Blössner, and E. Borghi, "Prevalence and trends of stunting among pre-school children, 1990-2020," *Public Health Nutr.*, vol. 15, no. 1, pp. 142–148, 2012.
[30] B. J. Akombi, K. E. Agho, J. J. Hall, D. Merom, T. Astell-Burt, and A. M. N. Renzaho, "Stunting and severe stunting among children under-5 years in Nigeria: A multilevel analysis," *BMC Pediatr.*, vol. 17, no. 1, pp. 1–16, 2017.
[31] S. Thurstans *et al.*, "Boys are more likely to be undernourished than girls: A systematic review

For peer review only

and meta-analysis of sex differences in undernutrition," *BMJ Glob. Heal.*, vol. 5, no. 12, 2020.
[32] Y. Y. Yang *et al.*, "Trends and determinants of stunting among under-5s: Evidence from the 1995, 2001, 2006 and 2011 Uganda Demographic and Health Surveys," *Public Health Nutr.*, vol. 21, no. 16, pp. 2915–2928, 2018.
[33] M. T. Ruel *et al.*, "Age-based preventive targeting of food assistance and behaviour change and communication for reduction of childhood undernutrition in Haiti: a cluster randomised trial," *Lancet (London, England)*, vol. 371, no. 9612, pp. 588–595, 2008.
[34] C. Desmond *et al.*, "Investing in human development and building state resilience in fragile contexts : A case study of early nutrition investments in Burkina Faso," *PLOS Glob. PUBLIC Heal.*, vol. 3, no. 3, pp. 1–15, 2023.
[35] Y. Sanoussi, B. O. Ahinkorah, A. Banke-Thomas, and S. Yaya, "Assessing and decomposing inequality of opportunity in access to child health and nutrition in sub-Saharan Africa: Evidence from three countries with low human development index," *Int. J. Equity Health*, vol. 19, no. 1, pp. 1–16, 2020.
[36] C. J. A. Bradshaw *et al.*, "Testing the socioeconomic and environmental determinants of better child-health outcomes in Africa: A cross-sectional study among nations," *BMJ Open*, vol. 9, no. 9, 2019.
[37] V. Dipasquale, U. Cucinotta, and C. Romano, "Acute Malnutrition in Children: Pathophysiology, Clinical Effects and Treatment," *Nutrients*, vol. 12, no. 8, pp. 1–9, Aug. 2020.
[38] A. Gelli *et al.*, "Improving diets and nutrition through an integrated poultry value chain and nutrition intervention (SELEVER) in Burkina Faso: Study protocol for a randomized trial," *Trials*, vol. 18, no. 1, Sep. 2017.

For peer review only

List of figures

Figure 1. Overall province-specific odd ratios (province random effect) of stunting (a), underweight (b) and wasting (c) among study population.
Maps created by Rouamba T. et al., 2021.

Figure 2. Overall region-specific odd ratios (region random effect) of stunting (a), underweight (b) and wasting (c) among study population.

For peer review only

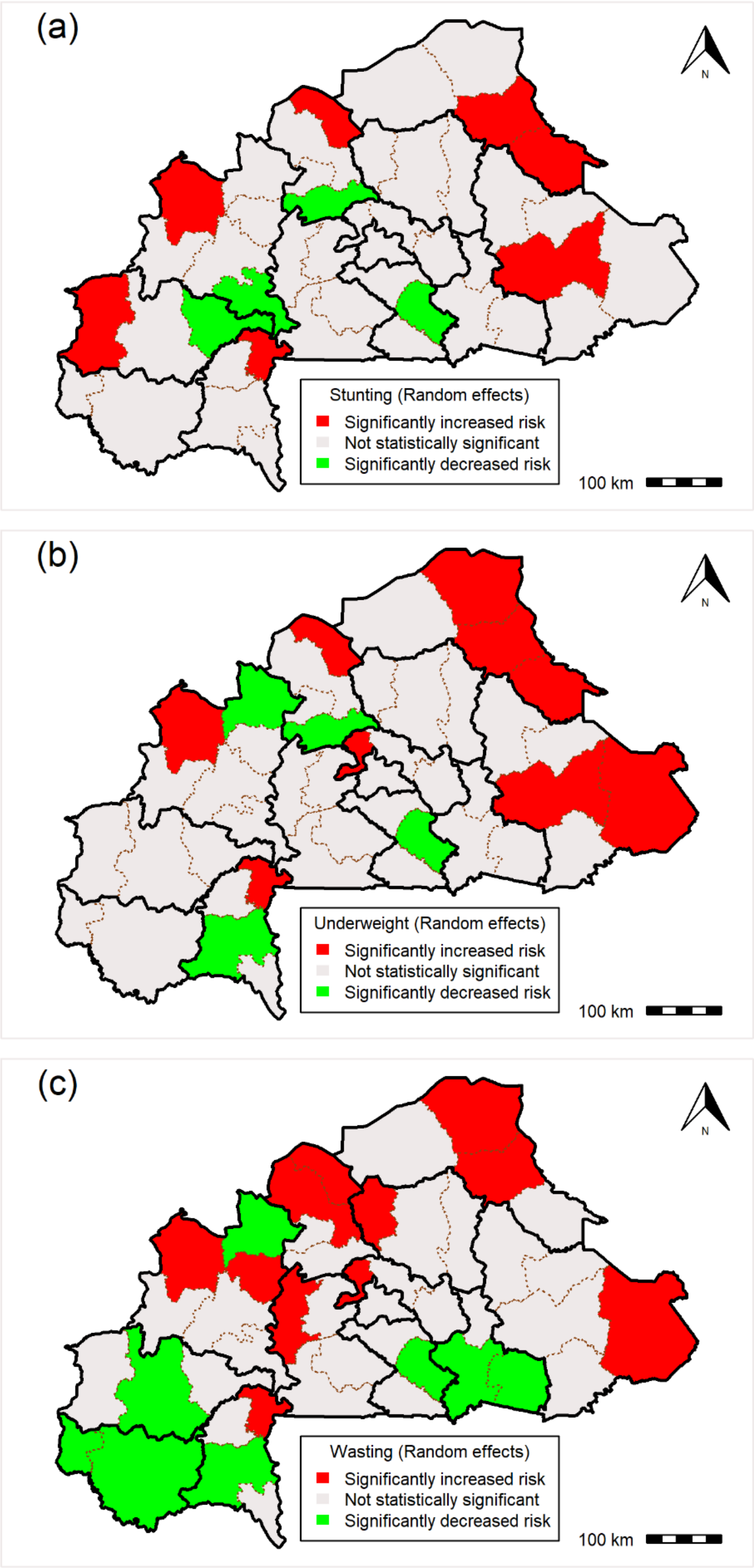

Figure 1. Overall province-specific odd ratios (province random effect) of stunting (a), underweight (b) and wasting (c) among study population.
Maps created by Rouamba T. et al., 2021.

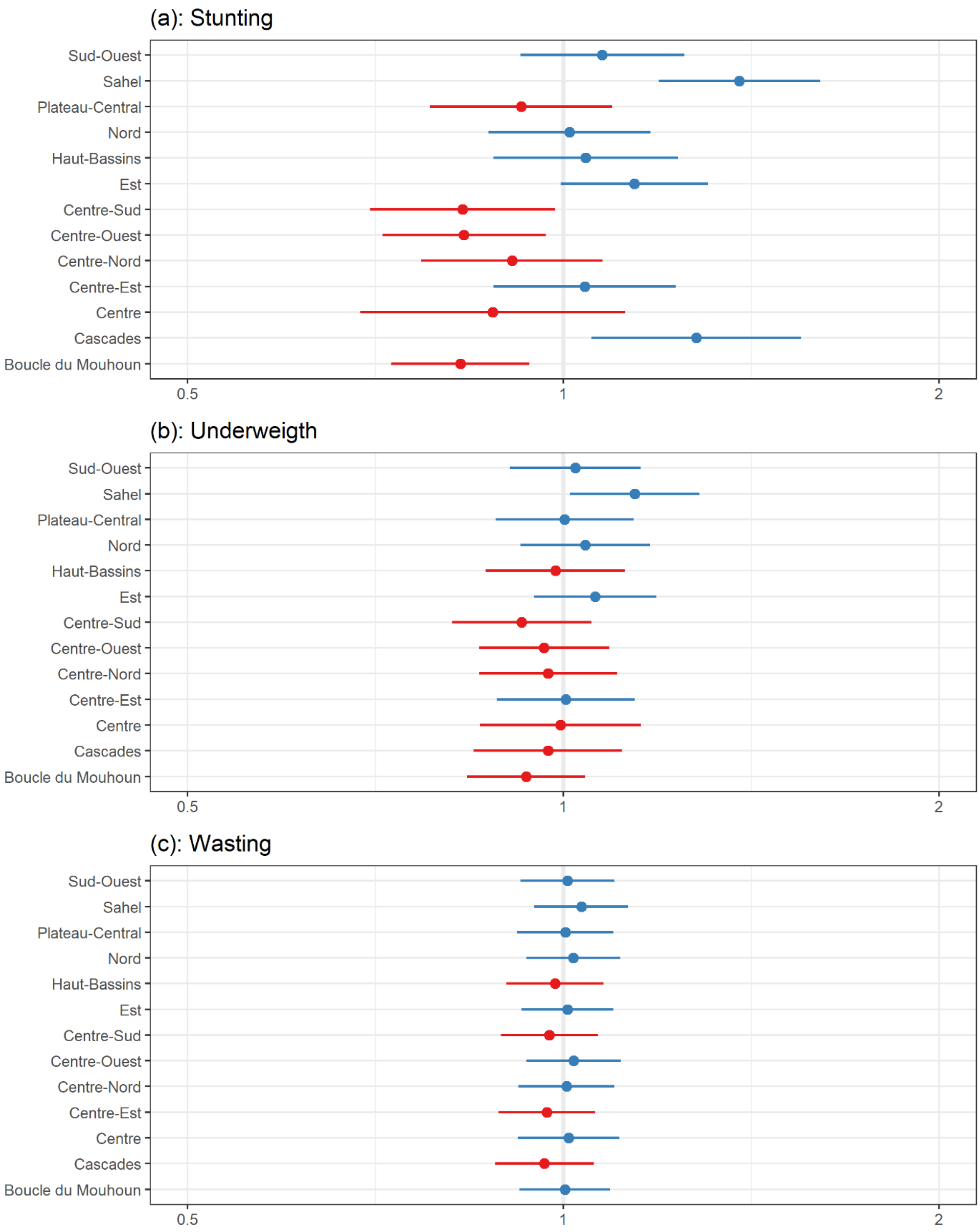

Figure 2. Overall region-specific odd ratios (region random effect) of stunting (a), underweight (b) and wasting (c) among study population.

Supplementary materials

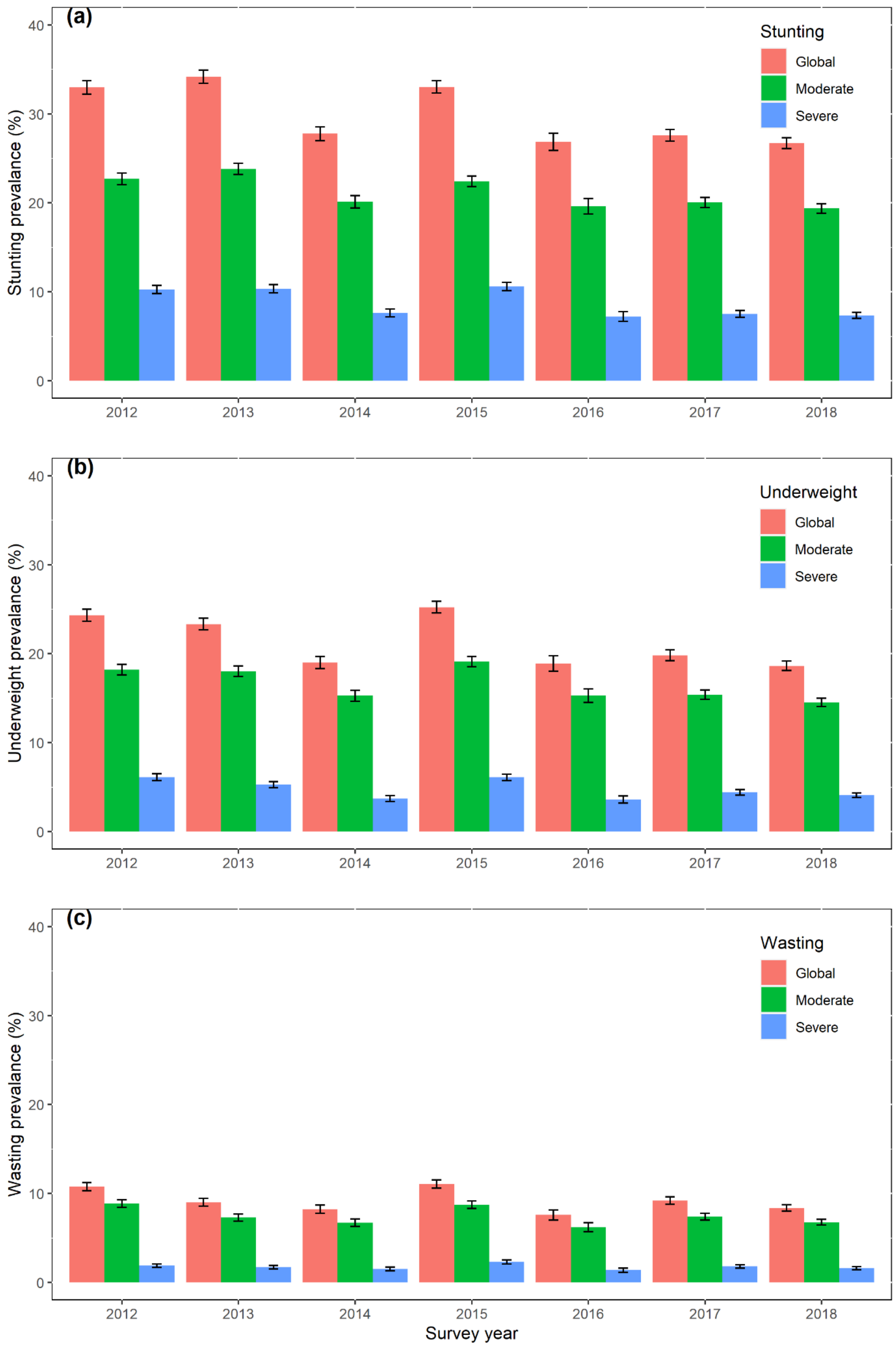

Supplemental Figure 1. Prevalence of stunting (a), underweight (b) and wasting (c). The plots demonstrate changes from 2012 to 2018. The error bars mark the 95% confidence intervals in panels a, b and c.

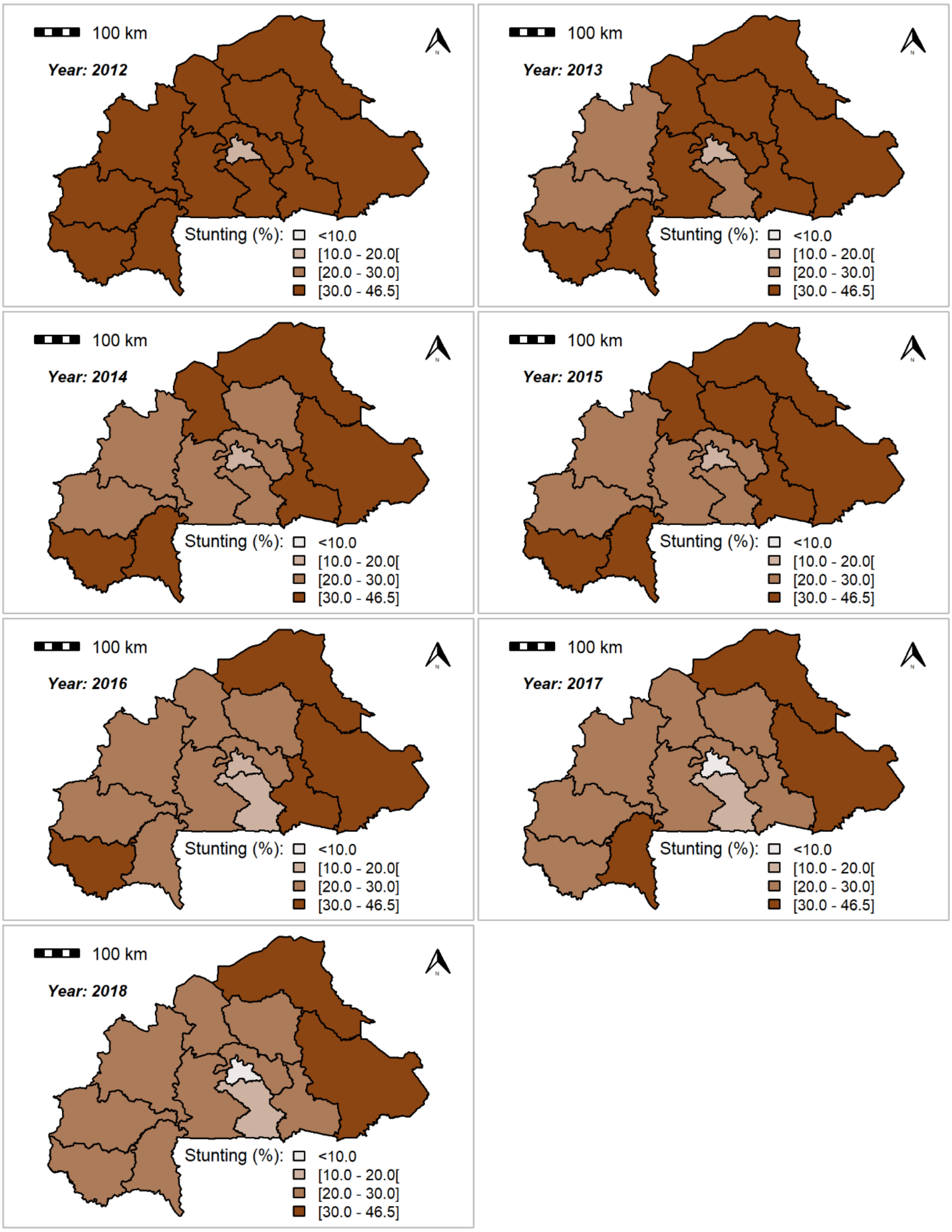

Supplemental Figure 2. Maps of geographical distribution at regional level of stunting among children aged 0–59 months surveyed in Burkina Faso, 2012 to 2018.
Maps created by Rouamba T. et al., 2021.

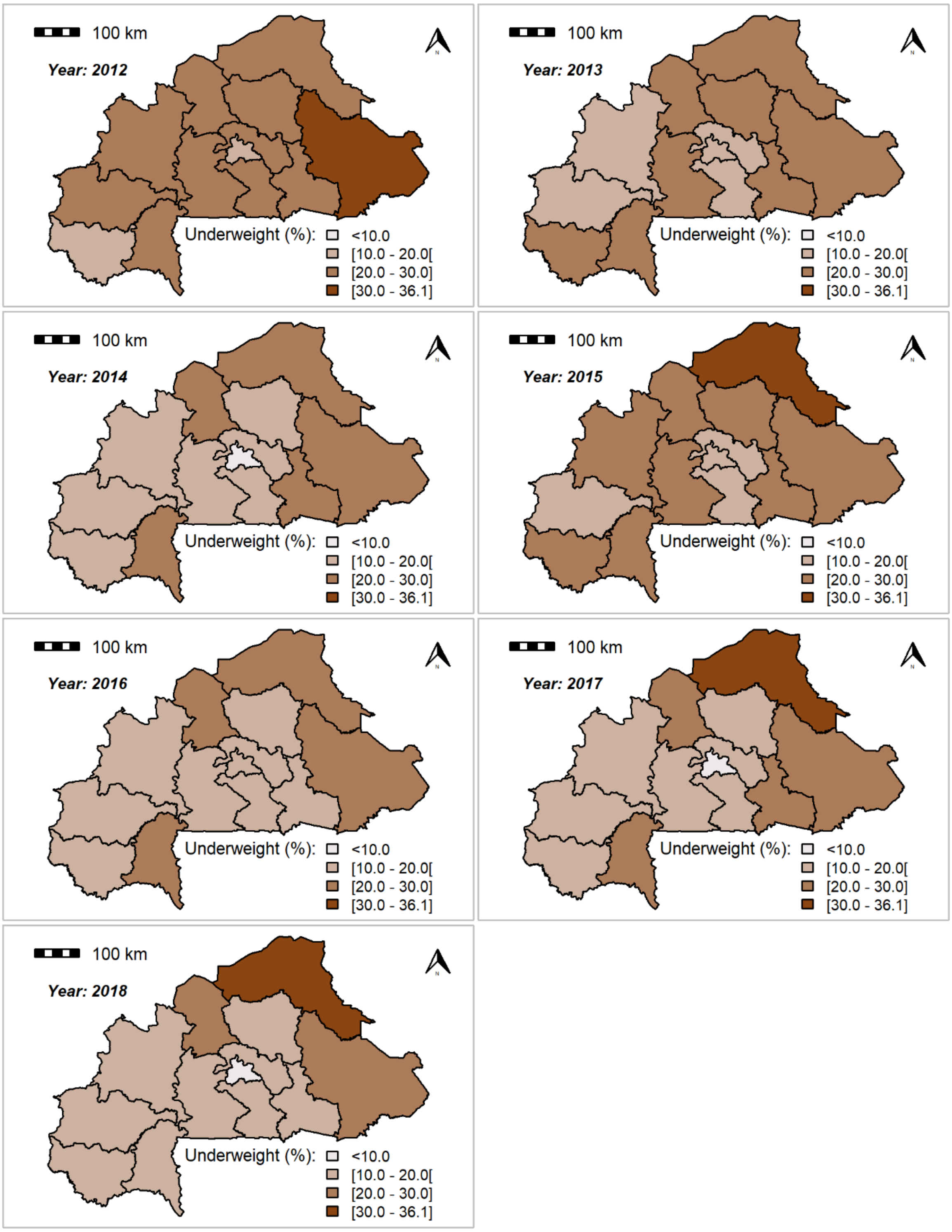

Supplemental Figure 3. Maps of geographical distribution at regional level of underweight among children aged 0–59 months surveyed in Burkina Faso, 2012 to 2018.
Maps created by Rouamba T. et al., 2021.

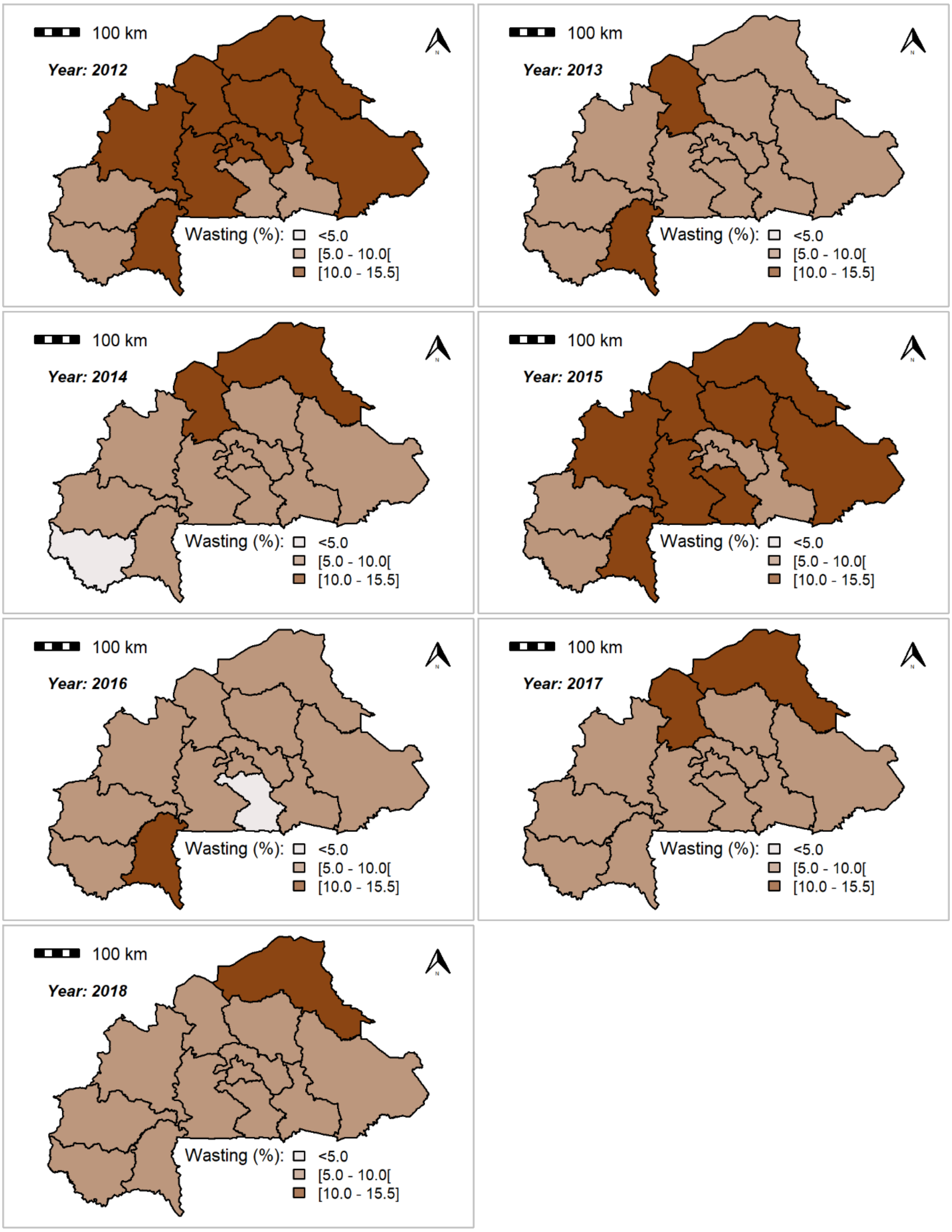

Supplemental Figure 4. Maps of geographical distribution at regional level of wasting among children aged 0–59 months surveyed in Burkina Faso, 2012 to 2018.
Maps created by Rouamba T. et al., 2021.

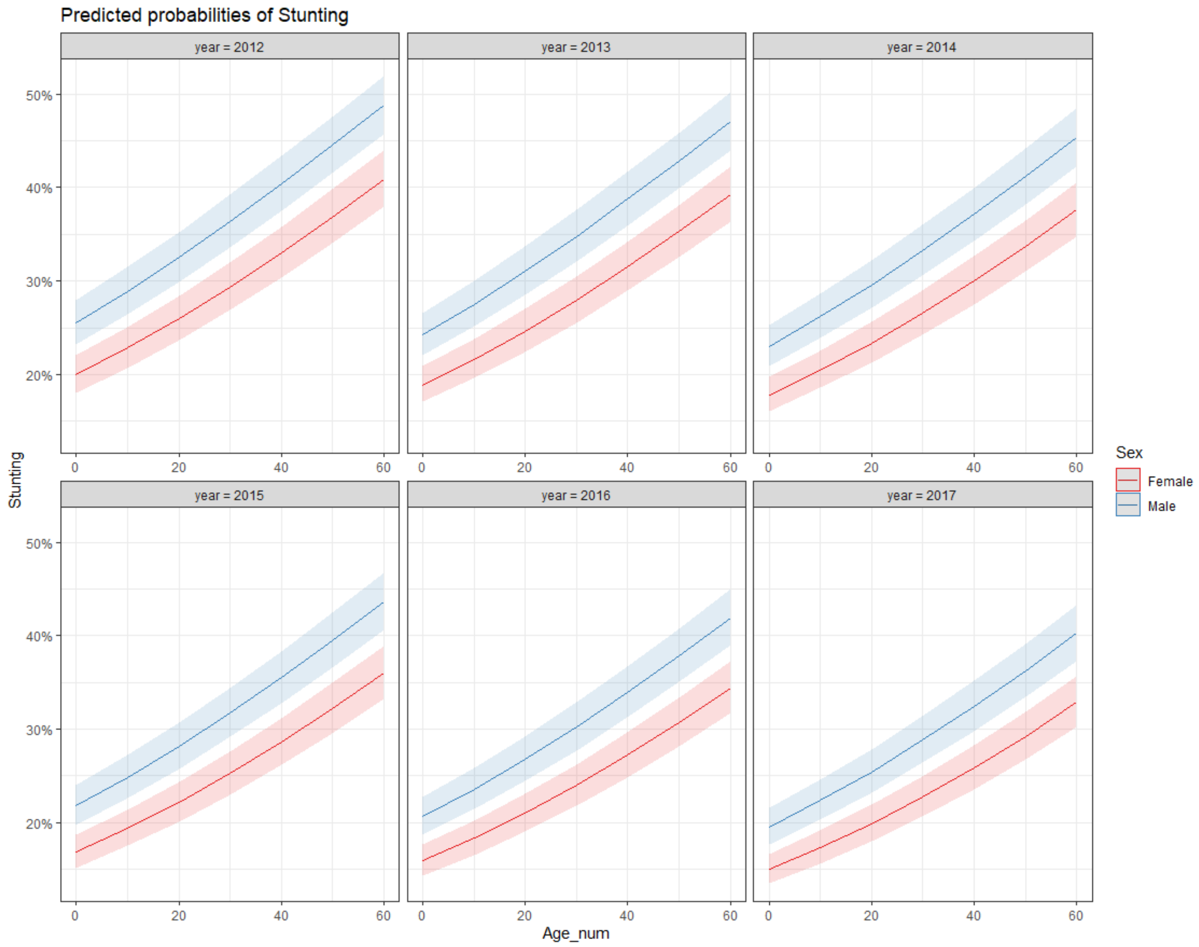

Supplemental Figure 5. Predicted probabilities of stunting according to the child age and stratified by gender in Burkina Faso, from 2012 to 2017.

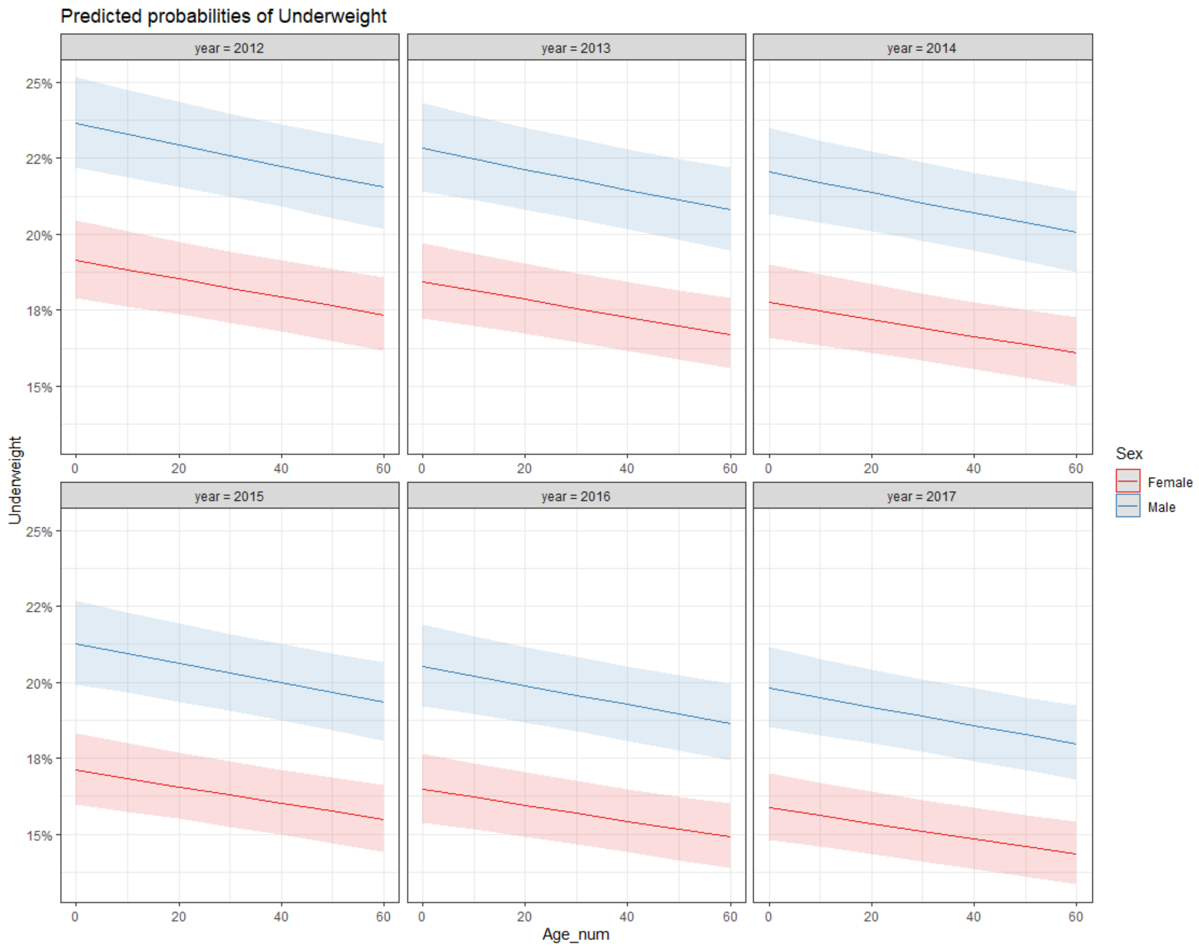

Supplemental Figure 6. Predicted probabilities of underweight according to the child age and stratified by gender in Burkina Faso, from 2012 to 2017.

view only

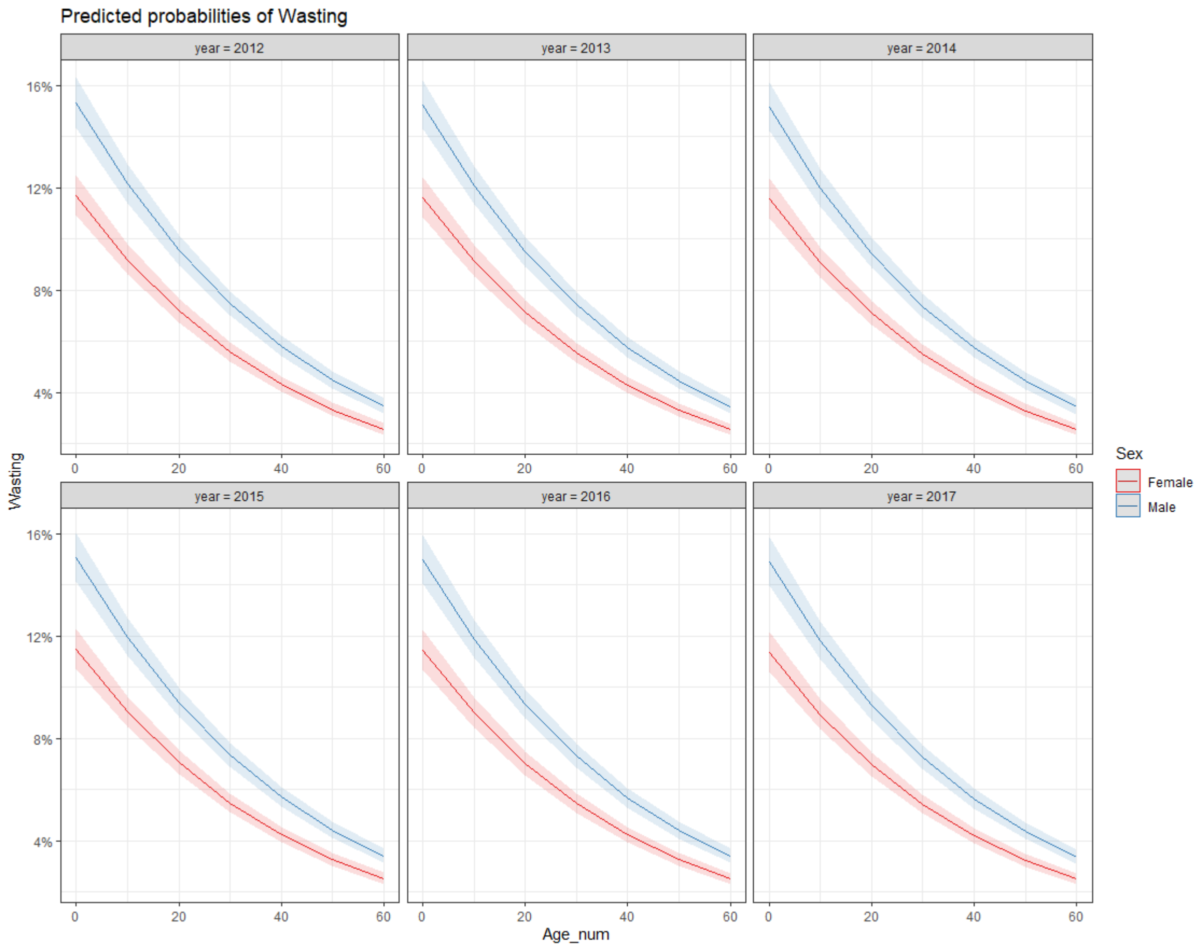

Supplemental Figure 7. Predicted probabilities of wasting according to the child age and stratified by gender in Burkina Faso, from 2012 to 2017.

view only

General linear mixed models (multilevel models) with binomial distribution were used to quantify the association of independent variables with each study outcome variable (stunting, underweight and wasting). The general linear mixed model contains fixed and random effects and was formulated as follow:

$$\boldsymbol{Y_{ij}} \sim Bernoulli\ (\pi_{ij})$$

$$logit\ (\pi_{ij}) = log\left[\frac{\pi_{ij}}{1-\pi_{ij}}\right] = \beta_0 + \sum_{p=1}^{P} \beta_p x_{ij} + \sum_{q=1}^{Q} \beta_q z_j + \omega_j + \varepsilon_i$$

In this general linear model, $\boldsymbol{Y_{ij}}$ represent stunting, underweight or wasting for a child i in a cluster j. The logit (π_{ij}) was used to model the probability of success (i.e., occurrence of undernutrition) as a linear combination of observed individual characteristics (x_{ij}) and contextual characteristics (z_j) associated with an unobserved specific effect (random effect) of the province and region (ω_j). The model contains a random error for child *i* (ε_i) that does not depend to the province/region nor year. The fixed effect is composed of $\boldsymbol{\beta_0}$ (intercept), $\boldsymbol{\beta_p}$ (vector of regression coefficients associated with the individual-level variables) and $\boldsymbol{\beta_q}$ (vector of regression coefficients associated with the cluster-level variables). The unobserved specific effect or random effect $(\boldsymbol{\omega_j})$ has two components, province specific effect and region specific effect. These cluster specific effects were considered as province and region specific risk.

All statistical analyses were performed with R statistical software (R Development Core Team, R Foundation for Statistical Computing, Vienna, Austria). The descriptive statistics that accounted for the complex survey design and sampling weights were performed using "survey" package, regression models were fitted using the "lme" package.

Model Diagnostics

We assume the response was drawn from a binomial distribution the residual are independent and normally distributed. The random effects are also, independent and normally distributed. The model diagnostics was check by generating a diagnostic that plots the fitted or predicted values against the residuals.

For peer review only

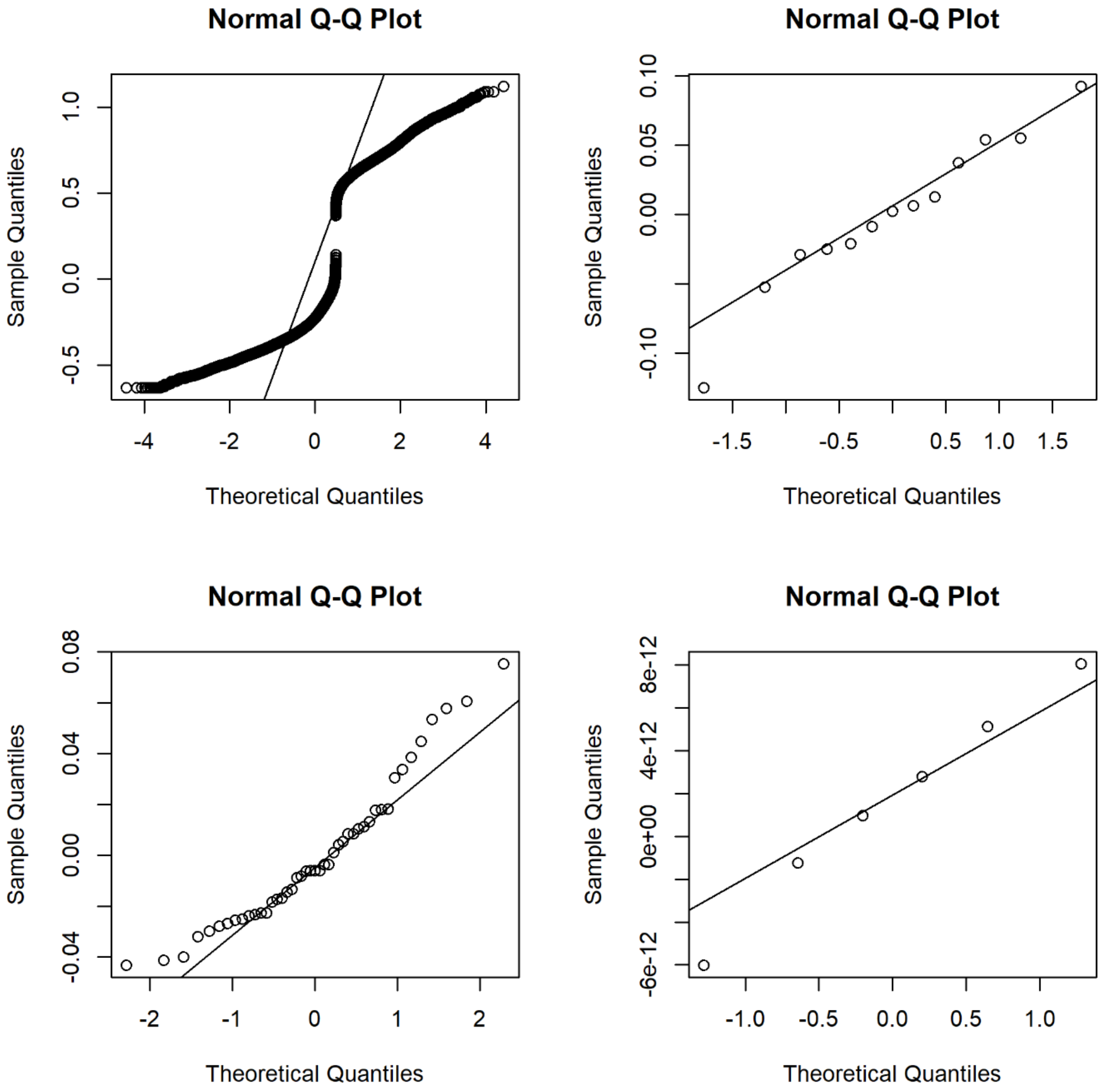

Supplemental Figure 8. Diagnostic plots for stunting multilevel model. Upper left: QQ plot of the residuals; upper right: QQ plot of the random effects (region); lower left: QQ plot of the random effects (province); lower right: QQ plot of the random effects (year)

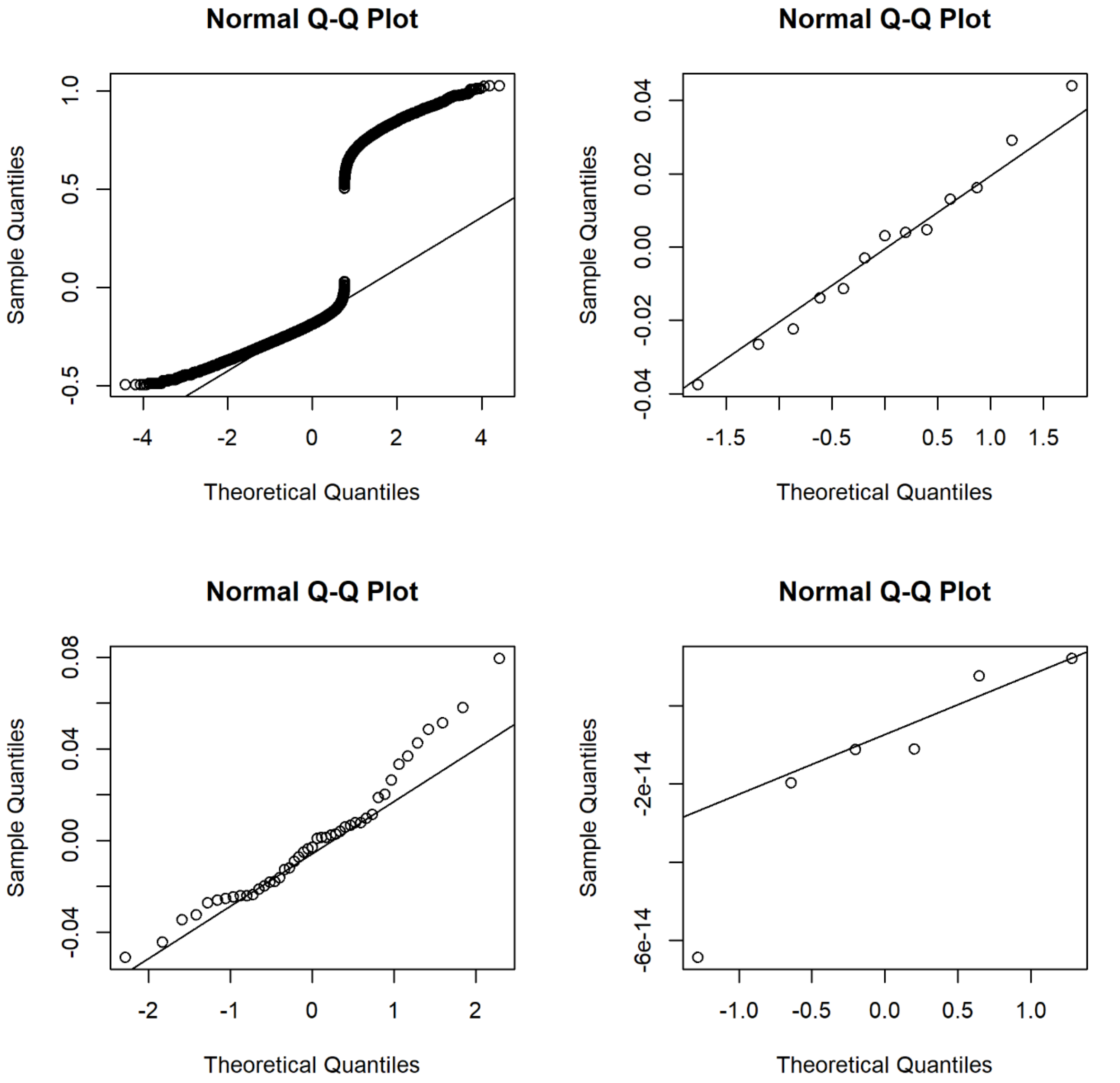

Supplemental Figure 9. Diagnostic plots for underweight multilevel model. Upper left: QQ plot of the residuals; upper right: QQ plot of the random effects (region); lower left: QQ plot of the random effects (province); lower right: QQ plot of the random effects (year)

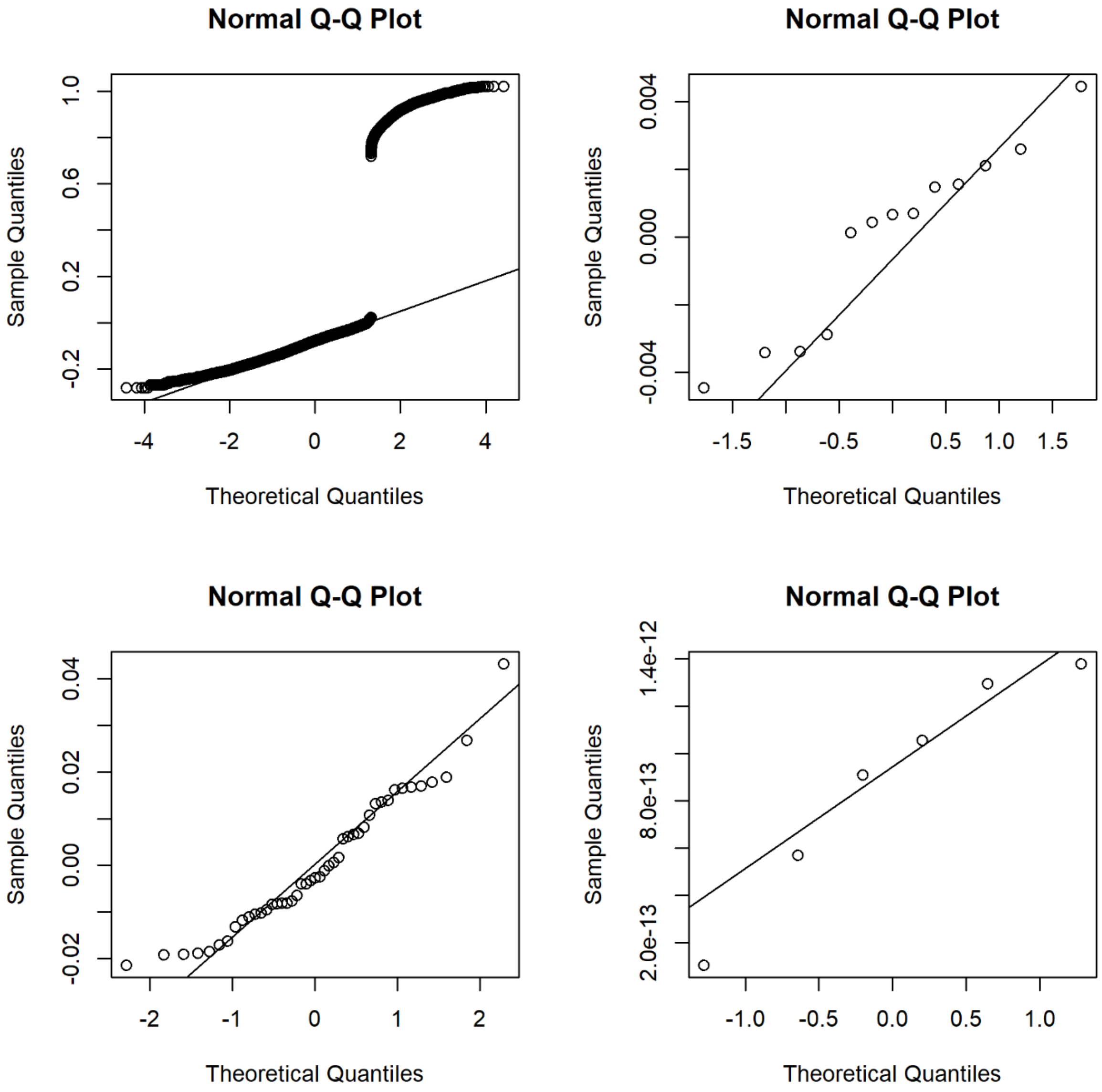

Supplemental Figure 10. Diagnostic plots for wasting multilevel model. Upper left: QQ plot of the residuals; upper right: QQ plot of the random effects (region); lower left: QQ plot of the random effects (province); lower right: QQ plot of the random effects (year)

BMJ Open

Undernutrition in children aged 0-59 months by region and over time : Secondary analysis of the Burkina Faso 2012-2018 National Nutrition Surveys.

Journal:	*BMJ Open*
Manuscript ID	bmjopen-2022-066509.R2
Article Type:	Original research
Date Submitted by the Author:	01-Aug-2023
Complete List of Authors:	Boua, Palwende; Institut de Recherche en Sciences de la Sante, Clinical Research Unit of Nanoro; University of the Witwatersrand Johannesburg, Sydney Brenner Institute for Molecular Biosciences<br>Rouamba , Toussaint; Institut de Recherche en Sciences de la Santé, Clinical Research Unit of Nanoro<br>Bambara, Estelle; Ministère de la Santé, Direction de la Nutrition<br>Kaboré, Saidou; Ministère de la Santé, Direction de la Nutrition<br>Compaore, Ella W. R.; Université Joseph Ki-Zerbo, Laboratoire de Biochimie, Biotechnologie, Technologie Alimentaire et Nutrition (LABIOTAN)<br>Ouedraogo, Boureima; Institut National de Statistique et de la Démographie<br>Tinto, Halidou; Institut de Recherche en Sciences de la Santé, Clinical Research Unit of Nanoro<br>Newell, Marie-Louise; University of Southampton, MRC Lifecourse Epidemiology Centre<br>Ward, Kate; University of Southampton, MRC Lifecourse Epidemiology Centre; University of Southampton, Global Health Research Institute<br>Sorgho, Hermann; Institut de Recherche en Sciences de la Santé, Clinical Research Unit of Nanoro
**Primary Subject Heading**:	Epidemiology
Secondary Subject Heading:	Nutrition and metabolism, Public health
Keywords:	EPIDEMIOLOGY, NUTRITION & DIETETICS, PUBLIC HEALTH

SCHOLARONE™
Manuscripts

I, the Submitting Author has the right to grant and does grant on behalf of all authors of the Work (as defined in the below author licence), an exclusive licence and/or a non-exclusive licence for contributions from authors who are: i) UK Crown employees; ii) where BMJ has agreed a CC-BY licence shall apply, and/or iii) in accordance with the terms applicable for US Federal Government officers or employees acting as part of their official duties; on a worldwide, perpetual, irrevocable, royalty-free basis to BMJ Publishing Group Ltd ("BMJ") its licensees and where the relevant Journal is co-owned by BMJ to the co-owners of the Journal, to publish the Work in this journal and any other BMJ products and to exploit all rights, as set out in our licence.

The Submitting Author accepts and understands that any supply made under these terms is made by BMJ to the Submitting Author unless you are acting as an employee on behalf of your employer or a postgraduate student of an affiliated institution which is paying any applicable article publishing charge ("APC") for Open Access articles. Where the Submitting Author wishes to make the Work available on an Open Access basis (and intends to pay the relevant APC), the terms of reuse of such Open Access shall be governed by a Creative Commons licence – details of these licences and which Creative Commons licence will apply to this Work are set out in our licence referred to above.

Other than as permitted in any relevant BMJ Author's Self Archiving Policies, I confirm this Work has not been accepted for publication elsewhere, is not being considered for publication elsewhere and does not duplicate material already published. I confirm all authors consent to publication of this Work and authorise the granting of this licence.

For peer review only

Title: Undernutrition in children aged 0-59 months by region and over time : Secondary analysis of the Burkina Faso 2012-2018 National Nutrition Surveys.

Palwende R. Boua[1,2Φ*], Toussaint Rouamba[1*], Estelle Bambara[3], Saidou Kaboré[3], Ella Compaoré[4], Boureima Ouedraogo[5], Halidou Tinto[1], Marie-Louise Newell[6], Kate Ward[6,7,8], Hermann Sorgho[1] on behalf of the INPreP Study Group

[1] Clinical Research Unit of Nanoro, Institut de Recherche en Sciences de la Santé, Burkina Faso

[2] Sydney Brenner Institute for Molecular Biosciences, University of the Witwatersrand, South Africa

[3] Direction de la Nutrition, Ministère de la Santé, Burkina Faso

[4] LABIOTAN, UFR/SVT, Université Joseph Ki-Zerbo, Ouagadougou, Burkina Faso

[5] Institut National de Statistique et de la Démographie, Ouagadougou, Burkina Faso

[6] MRC Lifecourse Epidemiology Centre, University of Southampton, United Kingdom

[7] Global Health Research Institute, School of Human Development and Health, Faculty of Medicine, University of Southampton, United Kingdom

[8] School of Public Health, Faculty of Health Sciences, University of the Witwatersrand, Johannesburg, South Africa

[Φ]Corresponding author: **Palwende Romuald Boua, PhD**
Clinical Research Unit of Nanoro, Institut de Recherche en Sciences de la Santé, Burkina Faso
218 CMS Ouaga 11
romyboua@gmail.com

*Equal contribution

Word count: 3094

For peer review only

Abstract

The global burden of undernutrition remains high, responsible for significant under-five mortality in resource-limited settings. Numerous sustainable development goals (SDGs) are linked to nutrition, and nationally representative nutrition surveillance is a key activity to track progress towards SDGs and guide efficient programs.

Objectives: The aim of this study is to look at spatial and temporal trends in undernutrition in children under five years age in Burkina Faso.

Setting: We used data from annual National Nutrition Surveys using SMART methodology (anthropometry, morbidity) over seven years (2012-2018) in Burkina Faso.

Participants: Children of under-five years from households selected through systemic sampling at countrywide level.

Main outcome measures: Prevalence of stunting (height-for-age, HAZ<-2), underweight (weight-for-age, WAZ<-2) and wasting (WHZ<-2) at regional and national. We used General Linear Mixed models, adjusted by age, survey year, sex, presence of fever and/or diarrhea, and poverty index to quantify risk of undernutrition over time and by region of residence.

Results: Between 2012 and 2018, decreases were observed overall in the prevalence of growth retardation (stunting) decreased from 33.0% (95%CI: 32.3 – 33.8) in 2012 to 26.7% (95%CI: 26.2 – 27.3) in 2018. Underweight reduced from 24.4% (95%CI: 23.7 – 25.1) to 18.7% (95%CI: 18.2 – 19.2) for the same period and wasting decreased from 10.8% (95%CI: 10.3 – 11.3) in 2012 to 8.4% (95%CI: 8.1 – 8.8) in 2018. However, there was substantial variation across the country, with increased risk of undernutrition in the regions of Sahel, East and Cascades mainly. High risk regions were characterized by a lower poverty index and limited access to healthcare services.

Conclusions: Our findings could inform national policymakers in refining and optimizing resource allocation based on identification of high-risk areas.

Key words: undernutrition, spatial, temporal, National Nutrional Suveys, Burkina Faso

For peer review only

Article Summary

Strengths and limitations of this study:

- Children morbidity is associated with all form of undernutrition.
- Poverty index is associated with chronic undernutrition but not with acute undernutrition.
- Under five boys are at higher risk of experiencing undernutrition compared to girls.
- Sahel region children are at higher risk of stunting and underweight.
- Our study did not cover all the National Nutrition Surveys from 2009 to 2021, due to data unavailability, therefore our finding is only relevant for the studied period. The analysis relies on survey data but not a cohort, hence causality cannot be inferred.

For peer review only

Background

Globally, undernutrition (eg wasting as a sign of acute undernutrition, and stunting as a sign of chronic undernutrition) remains an important public health issue, affecting the growth and mortality of children, especially in low- and middle-income countries [1]–[3]. Globally, in 2020, wasting and stunting affected an estimated 45.4 and 149.2 million children under 5 years of age, respectively [4]. [5], [6]. Undernourished children have a higher risk of death from common childhood illness such as diarrhoea, pneumonia, and malaria [5], [7], [8], and longer-term are likely to experience poorer physical and cognitive development, increased risk of chronic disease in older age, with undernourished women at increased risk of delivering low birth weight babies [9]–[11].

In Burkina Faso, like most low-income countries, improvements in nutrition though better diet quality and access to food, continues to be a major health, socioeconomic and political challenge, and is one of the main public health priorities towards achieving Sustainable Development Goals (SDGs) 1, 2, and 3 [12]. In Burkina Faso, more than one-third of deaths among children under five years of age are directly or indirectly attributable to undernutrition. Despite the development and implementation by successive governments of numerous policies and programs to deal with undernutrition, the prevalence of undernutrition (stunting, underweight and wasting) remains high. Factors such as recurrent drought, sociocultural practices (poor dietary practices), poverty, diarrheal and febrile diseases and insufficient access to basic health services negatively affect the nutritional status of children [3], [8], [13]–[15]**.** In addition, there is the problem of insecurity that the country has been experiencing since 2015, leading to massive internal displacement of the population, aggravating the already very limited access to basic livelihoods, health care and nutrition services in a context of poverty in these localities [16]–[18] [19], [20]. Food insecurity (lack of consistent access to enough food for every person in a household to live an active, healthy life) has been highly prevalent in the country (bulletin special FSC).

The government, supported by its financial and technical partners, has put in place a system to monitor the nutritional situation through the implementation of yearly national nutritional surveys according to the SMART (Standardized Monitoring and Assessment of Relief and Transitions) methodology. This methodology is widely used by governments and humanitarian partners to conduct timely nutrition surveys in all contexts (emergency, development, displaced populations). SMART surveys are conducted on a regular basis, often in connection with seasonal malnutrition, and can be conducted at the national or regional level, and even on a smaller scale. This paper follows a secondary analysis of the survey databases from 2012 to 2018 to have an overview of temporal trends and overall spatial risk of undernutrition and identify the areas to prioritize for resource allocation according to the risk of areas.

Methods

Study setting

Burkina Faso is a landlocked country in West Africa divided into 13 regions, 45 provinces, 351 communes (49 urban and 302 rural) and 8228 villages. According to the latest census, the country has 20.5 million inhabitants, 45.3% of whom are under 15 years old and children aged 0-4 years represent 16.2% of the total population [21]. The country is ranked 182nd out of 189 according to the Human Development Index report with more than 41.4% of its population living below the poverty line [22].

The country's epidemiological profile is marked by the persistence of a high burden of disease due to infectious disease, and by the progressive increase in the burden of non-communicable diseases. Beside the infectious and non-communicable diseases, undernutrition is one of the major diseases of public

For peer review only

health importance [23]. The food security situation is also of concern. It is estimated that approximately 3,28 million people are food insecure and in need of immediate humanitarian assistance, representing 15% of the country's total population [24].

Patient and public involvement

Because we used data from National Nutrition Surveys, it was not appropriate or possible to involve patients or the public in the design, or conduct, or reporting, or dissemination plans of our research.

Data sources

The Directorate of the Nutrition of Burkina Faso provided data (accessible through upon request). The data are from annual cross-sectional National Nutrition Surveys using the SMART methodology. This consists of a rapid assessment survey of the food and nutrition situation among children under 5 years of age, women of childbearing age and households. For this study, we focus on anthropometric measurements collected between 2012 and 2018 (seven surveys) among population aged 0-59 months.

We examined trends in three crude indices of nutritional problems in Burkina Faso over a seven-year period, namely stunting (height/length-for-age, L/HFA), underweight (weight-for-age, WFA) and wasting (weight-for-height/length, WFH/L). Anthropometric measurements were converted into weight-for-age z-score (WAZ), height-for-age z-score (HAZ), and weight-for-height z-score (WHZ), using the 2006 World Health Organization (WHO) child growth standards [25]. Children with oedema, which can be a sign of acute undernutrition) were excluded as recommended for this analysis (**Table 1**).

Table 1. Definition of anthropometric measurements and indices in children under five

Index	Nutritional problem measured	Indicator
Height/Length-for-age (H/LFA)	Severe stunting	HAZ < −3 SD
	Moderate stunting	HAZ < −2 SD and HAZ ≥ −3 SD
	Global stunting	HAZ < −2 SD
Weight-for-age (WFA)	Severe underweight	WAZ < −3 SD
	Moderate underweight	WAZ < −2 SD and WAZ ≥ −3 SD
	Global underweight	WAZ < −2 SD
Weight-for-Height/Length (WFH/L)	Severe wasting	WHZ < −3 SD
	Moderate wasting	WHZ < −2 SD and WHZ ≥ −3 SD
	Global wasting	WHZ < −2 SD

HAZ: Length/Height-for-age z-score; WAZ: Weight-for-age z-score; WHZ: Weight-for-length/height z-score

For 2012 and 2014, the SMART survey was provincially representative for six regions (Cascades, Centre West, East, North, Sahel and South West) and regionally representative for the other seven (Boucle du Mouhoun, Centre-South, Centre, Centre-North, Centre-East, Central Plateau and Hauts-Bassins). For 2013 and 2015, the SMART survey had provincial representativeness for seven regions (Boucle du Mouhoun, Centre-South, Centre, Centre-North, Centre-East, Central Plateau and Hauts-Bassins) and regional representativeness for the other six (Cascades, Centre-West, East, North, Sahel and South-

For peer review only - http://bmjopen.bmj.com/site/about/guidelines.xhtml

West). For 2016, the SMART survey had regional representativeness for 13 regions (resulting in smaller sample) and from 2017 onwards, the SMART survey had provincial representativeness for the 45 provinces across the 13 regions.

Each level of representativeness constituted strata.

Outcome variables

Stunting was determined if HAZ < −2 SD, underweight as WAZ < −2 SD and wasting as WHZ < −2 SD (Table 1). We excluded z-scores with extreme values from the observed mean, the following WHO indicators were used in the final analysis: WHZ - 5 to 5; HAZ - 6 to 6; WAZ - 6 to 5.

The independent variables were individual-level characteristics mainly age in months and gender, diarrhea and fever during the 2 weeks preceding the survey, year of the survey and cluster-level characteristics constituted by the poverty index for each province [26].

Statistical analysis

For descriptive statistics at the individual and strata levels, estimates account for the complex survey design and the sampling weights provided by *survey*. Descriptive analysis of the study population was presented using means and standard error for continuous variables and frequencies and proportions for categorical variables. These descriptive analyses were performed to estimate the crude temporal trend of prevalence nationwide and provide a yearly map for the regional disparities of undernutrition index.

Considering that the data have a hierarchical structure, and we would need to account for the cluster (province and region) variability as well as the individual-to-individual variability, we used General linear mixed models (multilevel models). GLMM (multilevel models) with binomial distribution were used to quantify the association of independent variables with each study outcome variable (stunting, underweight and wasting). We assume that the outcomes variables were drawn from a binomial distribution and the residual are independent and normally distributed. The general linear mixed model contains fixed and random effects and was formulated as follow:

$\boldsymbol{Y_{ij}} \sim Bernoulli\ (\pi_{ij})$

$$logit\ (\pi_{ij}) = log\left[\frac{\pi_{ij}}{1-\pi_{ij}}\right] = \beta_0 + \sum_{p=1}^{P} \beta_p x_{ij} + \sum_{q=1}^{Q} \beta_q z_j + \omega_j + \varepsilon_i$$

ε_i~N(0,1)

The random effects are also, independent and normally distributed.

In this general linear model, $\boldsymbol{Y_{ij}}$ represent stunting, underweight or wasting for a child i in a cluster j. The logit (π_{ij}) was used to model the probability of success (i.e., occurrence of undernutrition) as a linear combination of observed individual characteristics (x_{ij}) and contextual characteristics (z_j) associated with an unobserved specific effect (random effect) of the province and region (ω_j). The model contains a random error for child *i* (ε_i) that does not depend to the province/region nor year. The fixed effect is composed of $\boldsymbol{\beta_0}$ (intercept), $\boldsymbol{\beta_p}$ (vector of regression coefficients associated with the individual-level variables) and $\boldsymbol{\beta_q}$ (vector of regression coefficients associated with the cluster-level variables). The unobserved specific effect or random effect $(\boldsymbol{\omega_j})$ has two components, province specific effect and region-specific effect. These cluster specific effects were considered as province and region-specific risk.

Since we intended to quantify the association between the outcomes and dependent variables by avoiding that the coefficient be affected by the individual weight, survey weights were not used to adjust the regressions and a complete case analysis was used. The model diagnostics was check by

For peer review only

generating a diagnostic that plots the fitted or predicted values against the residuals. All statistical analyses were performed with R statistical software (R Development Core Team, R Foundation for Statistical Computing, Vienna, Austria). The descriptive statistics that accounted for the complex survey design and sampling weights were performed using "survey" package, regression models were fitted using the "lme" package.

Results

Demographic characteristics and anthropometrical measures of children

The overall number of children under five assessed for their nutritional status using anthropometric measurement and included in the present analysis is showed in the Table 2. Since the survey in 2016 was set up to have regionally representative estimates, 9434 children were surveyed. Gender was equally distributed through the surveys and varied between 51.6% and 51.1% for the proportion of male children. The average age of the children during the period of 2012 to 2018 ranged from 27 to 29 months.

The distribution of anthropometric measures such as weight, height and the z-scores with WHO as reference population (HAZ, WAZ and WHZ) from 2012 to 2018 are presented in the Table 2 below. All z-scores had a mean value below the WHO standard. HAZ values ranged from -1.50 to -1.27; WHZ values ranged from -0.70 to -0.53; WAZ values ranged from -1.33 to -1.11.

Table 2. Characteristic of Children under five Surveyed at household in Burkina Faso, 2012 to 2018

	Weighted Mean (standard error)						
Index	**2012** N= 19049	**2013** N= 19170	**2014** N= 15835	**2015** N= 20408	**2016*** N= 9434	**2017** N= 19409	**2018** N= 23939
Age	27.80 (0.11)	27.15 (0.12)	27.82 (0.13)	27.47 (0.11)	28.59 (0.16)	28.53 (0.11)	29.04 (0.10)
Weight	10.46 (0.02)	10.39 (0.02)	10.72 (0.03)	10.41 (0.02)	10.85 (0.03)	10.8 (0.02)	10.92 (0.02)
Height	82.08 (0.09)	81.54 (0.1)	82.68 (0.11)	81.97 (0.09)	83.38 (0.13)	83.31 (0.09)	83.75 (0.08)
HAZ	-1.49 (0.01)	-1.50 (0.01)	-1.30 (0.01)	-1.47 (0.01)	-1.30 (0.01)	-1.28 (0.01)	-1.27 (0.01)
WHZ	-0.67 (0.01)	-0.61 (0.01)	-0.53 (0.01)	-0.70 (0.01)	-0.55 (0.01)	-0.60 (0.01)	-0.56 (0.01)
WAZ	-1.31 (0.01)	-1.28 (0.01)	-1.11 (0.01)	-1.33 (0.01)	-1.12 (0.01)	-1.15 (0.01)	-1.11 (0.01)

* In 2016 the sampling framework was regional and national (without provincial), hence impacting the sample size. This choice by the Ministry of Health was guided by limited resources to run the national survey.

Prevalence of undernutrition

The annual prevalence of chronic undernutrition (stunting) using HAZ ranged from 33.0% (95%CI: 32.3 – 33.8) in 2012 to 26.7% (95%CI: 26.2 – 27.3) in 2018, with a maximum of 34.2% (95%CI: 33.4 – 34.9) in 2013. In other words, between 2013 and 2018, stunting prevalence declined among children under five from 33.4% to 26.7%, a decline of 19.1% (Supplemental Figure 1 (a)).

The annual prevalence of underweight (WAZ) ranged from 24.4% (95%CI: 23.7 – 25.1) in 2012 to 18.7% (95%CI: 18.2 – 19.2) in 2018, with a maximum of 25.3% (95%CI: 24.6 - 25.9) in 2015 (Supplemental Figure 1 (b)). This meant that nationally, underweight decreased by 23.3% between 2012 and 2018.

The annual prevalence of wasting (WHZ) (Supplemental Figure 1 (c)) ranged from 10.8% (95%CI: 10.3 – 11.3) in 2012 to 8.4% (95%CI: 8.1 – 8.8) in 2018, with a maximum of 11.7% (95%CI: 10.7 – 11.6) in 2015. In other words, between 2013 and 2018, global wasting prevalence declined among children under five from 33.4% to 26.7%, corresponding to a decline of 22.2%.

Overall, there was a reduction in prevalence of undernutrition over the years, but this was not seen in all areas. In other words, there was a spatial heterogeneity of undernutrition index through the country.

During the seven years, the analysis showed a permanent high prevalence (>30%) of stunting in Sahel, and East regions (Supplemental **Figure 2**), whereas the stable high prevalence of underweight (>30%) was mainly noted in the Sahel region (Supplemental **Figure 3**). The high prevalence of wasting was mainly noted in the Sahel and North region (Supplemental **Figure 4**).

The predicted probabilities of stunting, underweight, wasting by child's age at survey, and stratified by gender for the year 2012 to 2017 are shown in Supplemental Figures 5, 6 and 7. The probability of being stunted increased with age, and was higher for male than female children, but decreased over time, i.e. between 2012 and 2018 (Supplemental **Figure 5**). Similarly, predicted prevalence of underweight decreased with children age, was greater among male children and declined over the study period i.e. from 2012 to 2018 (Supplemental **Figure 6**). For wasting, the probability decreased with child's age and was more marked among males, but the trend over time was stable (Supplemental **Figure 7**).

Potential risk factors associated with Stunting, Underweight, and Wasting from 2012 to 2017 in Burkina Faso

In multivariable analysis, allowing for adjustment (Table 3) males had a significantly increased risk of stunting (aOR=1.47, 95%CI: 1.43 – 1.52), underweight (aOR=1.39, 95%CI: 1.34 – 1.43) and wasting (aOR=1.38, 95%CI: 1.31 – 1.44) than females. Compared with children aged less than 5 months, older children had higher risks of stunting and underweight, but the odds of wasting decreased significantly over the age of 24 months. Compared with the year 2012, the analysis showed that the risk of stunting, underweight and wasting in the subsequent years, i.e. 2013 to 2018, decreased significantly overall, except for the year 2015 for wasting. Furthermore, diarrhea was associated with high odds of stunting (aOR=1.08, 95%CI: 1.05 – 1.12), underweight (aOR=1.32, 95%CI: 1.27 – 1.37) and wasting (aOR=1.46, 95%CI: 1.39 – 1.54). This association was similar with the fever. There was no significant association between wasting and poverty index (aOR=1.03, 95%CI: 0.96 – 1.10), whereas stunting and underweight were significantly associated with the poverty index. The model diagnostics plots were presented in supplementary materials (Text and Supplemental Figure 8, 9 and 10).

Table 3. Potential risk factors associated with Stunting, Underweight, and Wasting from 2012 to 2017 in Burkina Faso

	Stunting		Underweight		Wasting	
Predictors	aOR (95%CI)	p	aOR (95%CI)	p	aOR (95%CI)	p
Gender						
Female	1		1		1	
Male	1.39 (1.35 – 1.43)	<0.001	1.31 (.27 – 1.35)	<0.001	1.37 (1.31 – 1.43)	<0.001
Age in Month						
00 - 05	1		1		1	
06 - 11	1.51 (1.39 – 1.63)	<0.001	1.96 (1.82 – 2.11)	<0.001	1.67 (1.53 – 1.82)	<0.001
12 - 23	3.74 (3.49 – 4.00)	<0.001	2.56 (2.39 – 2.73)	<0.001	1.53 (1.41 – 1.65)	<0.001
24 - 35	4.94 (4.62 – 5.28)	<0.001	2.28 (.13 – 2.44)	<0.001	0.83 (0.76 – 0.90)	<0.001
36 - 47	4.57 (4.27 – 4.89)	<0.001	1.67 (1.56 – 1.79)	<0.001	0.46 (0.41 – 0.50)	<0.001
48 - 59	3.44 (3.20 – 3.68)	<0.001	1.58 (1.47 – 1.69)	<0.001	0.49 (0.44 – 0.54)	<0.001
Year of survey						
2012	1		1		1	
2013	0.89 (0.85 – 0.94)	<0.001	0.79 (0.75 – 0.84)	<0.001	0.72 (0.66 – 0.78)	<0.001
2014	0.78 (0.75 – 0.82)	<0.001	0.72 (0.69 – 0.76)	<0.001	0.73 (0.68 – 0.79)	<0.001
2015	0.87 (0.82 – 0.91)	<0.001	0.95 (0.90 – 1.00)	0.063	1.00 (0.92 – 1.08)	0.902
2016	0.73 (0.69 – 0.78)	<0.001	0.74 (0.69 – 0.79)	<0.001	0.70 (0.63 – 0.77)	<0.001
2017	0.67 (0.64 – 0.71)	<0.001	0.71 (0.68 – 0.75)	<0.001	0.86 (0.80 – 0.93)	<0.001
Diarrhea						
No	1		1		1	
Yes	1.19 (1.14 – 1.23)	<0.001	1.36 (1.31 – 1.42)	<0.001	1.39 (1.32 – 1.47)	<0.001
Fever						

For peer review only

No	1		1		1	
Yes	1.08 (1.05 – 1.12)	<0.001	1.31 (1.27 – 1.35)	<0.001	1.48 (1.41 – 1.55)	<0.001
Poverty Index in %	1.17 (1.09 – 1.26)	<0.001	1.15 (1.08 – 1.22)	<0.001	1.03 (0.96 – 1.10)	0.456
**Random Effects**						
σ2	3.29		3.29		3.29	
τ00						
Provinces	0.03		0.03		0.04	
Region	0.03		0.01		0.00	
ICC	0.02		0.01		0.01	
N Region	13		13		13	
N Provinces	45		45		45	
Observations	83161		83161		83161	

Estimation of province and region-specific risk

The province spatial risk of stunting, underweight and wasting was shown to be heterogeneous throughout the country. Figure 5 displays the specific risks (or odds) of each province compared with the national mean odds ratio (aOR = 1).

For stunting, the provinces with high odds were located in eight provinces namely Gourma, Ioba, Kenedougou, Kossi, Lorum, Seno and Yagha provinces, whereas the provinces with lower odds were located in four provinces namely, Bales, Passore, Tuy, and Zounweogo provinces (**Figure 1 (a)**).

Regarding underweight, the provinces with high odds were located in nine provinces namely Gourma, Ioba, Kossi, Kourweogo, Lorum, Oudalan, Seno, Tapoa, Yagha provinces, whereas the provinces with lower odds were located in four provinces namely, Passore, Poni, Sourou and Zounweogo provinces (**Figure 1 (b)**).

Regarding wasting, the provinces with high odds were located in 11 provinces namely, Bam, Ioba, Kossi, Kourweogo, Lorum, Nayala, Oudalan, Sanguie, Seno, Tapoa and Yatenga provinces whereas, the provinces with lower odds were located in eight provinces namely, Boulgou, Comoe, Houet, Koulpelgo, Leraba, Poni, Sourou, and Zounweogo province (**Figure 1 (c)**).

Further, the analysis also indicated some regions where the odds was statistically significant higher (Sahel and Cascades) or lower (Centre-sud, centre-Ouest and Boucle de Bouhoun) for stunting (**Figure 2 (a)**). For underweight, only the region of the Sahel exhibited higher odds (**Figure 2 (b)**). Finally, regarding wasting, no region exhibited a higher or lower risk (**Figure 2 (c)**).

Discussion

This study undertook a secondary analysis using data from annual cross-sectional National Nutrition Survey (NNS) using SMART methodology to generate estimates that could help national policymakers in refining and optimizing resource allocation based on identification of high-risk areas. The analysis found that undernutrition remains very high in certain part of the country, although there was an overall decrease in prevalence and risk over the years in the country; the geospatial risk to experience an undernutrition event is not uniform throughout the country. This decrease of all undernutrition prevalence has been observed in other sub-Saharan Africa countries despite predicted stagnation in the region for stunting until 2020 [27]–[29].

In previous analysis, De Onis and collaborators predicted that in Africa, the prevalence of stunting was going to stagnate in next coming decade from 2010, and specifically in Western Africa [29]. Our results showed a decrease of stunting prevalence of 6.7 between 2012 and 2018. De Onis prediction were based on poor data from some countries with lag time of about ten years between them (namely Demographic

For peer review only

and Health Survey), and the landscape of interventions in nutrition was poor. From 2009, a nutrition surveillance system has been implemented with yearly national surveys, additionally more effective nutrition programs to improve nutritional status across the whole population have been implemented.

Sex of child (male), two weeks morbidity (fever, diarrhea) were factors associated with the three forms of undernutrition; whereas poverty measured through wealth index was associated with stunting and underweight but not wasting. In their analysis of Nigeria Demographic and Health Survey (NDHS), Akombi et al. found the same factors associated to stunting[30]. Biological and social mechanism or both has been proposed to explain the differences between boys and girls [31]. Even though two weeks morbidity might not be causal to chronic undernutrition, it translates the effect of morbidity and or repeated morbidity on growth retardation and underweight.

Living in certain provinces and/or regions of the country is conferring higher risk of suffering from undernutrition. Similar findings were reported in Côte d'Ivoire, Uganda and Nigeria [28], [30], [32]. In Burkina Faso, these areas were the one with the highest proportion of the population living more than 10 Km away from the primary health center (first level of referral) [23]. This means that access to health care could be associated with nutritional status. The persistent prevalence of undernutrition translate a need, not just for the community management of wasting programs which have been shown to be effective, but for effective prevention programs as well as structural changes allowing reduction of inequalities [33]–[36].

Sahel region had the highest risk of stunting and underweight. Prior to the recent threats from terrorism, Burkina Faso had already endured decade of a fragile food security situation due to droughts and floods, affecting food security and nutrition, especially in the north part of the country. Restrictions on the movement of people and goods, as well as limited access to natural resources and regional or international markets, had placed a heavy burden on local food systems and people's livelihoods. The food insecurity situation in Burkina Faso is currently aggravated by a high level of insecurity. The populations of the Sahel zone are the most affected. Undernutrition is chronic and its causes are structural and complex. They are the result of a combination of several factors, including a short agricultural production season lasting four months of the year, climatic vulnerability, the depletion of natural resources, a chronic shortage of agricultural production, and very little access to basic social services due to the weak state presence.

Insecurity marked by attacks from terrorist groups and the increase in inter-community conflicts exacerbate food and nutrition insecurity in the Sahel and North Central regions by reducing the already fragile livelihoods of populations that depend mainly on agriculture and pastoralism. Insecurity limits access to fields and agricultural production. The resulting road closures affect the supply of agricultural products to markets, reduce the availability of agricultural products, limit access due to price fluctuations, and hinder transhumance and access of animals to pasture within Burkina and to Mali and Niger. The looting of food reserves and livestock reduces the resilience of communities affected by this insecurity. In addition, many health facilities are closed or operating at minimal capacity in areas affected by insecurity, reducing access to health services, including those for severely malnourished children. This has forced people to move to other parts of the country (almost one million internally displaced persons (IDPs) according to the United Nations Office for the Coordination of Humanitarian Affairs), resulting in overcrowding in host towns. While some IDPs reside in camps, more than 80% of IDPs are staying with host families whose food supplies are rapidly running out.

In our study, we found that poverty index is not associated with wasting, that mean acute undernutrition is due to an underlying disease causing abnormal nutrient loss, increased energy expenditure, or decreased food intake. Based on its etiology, undernutrition is either illness related (one or more diseases or injuries directly result in nutrient imbalance) or caused by environmental/behavioral factors associated with decreased nutrient intake and/or delivery [11], [37],

For peer review only

[38]. Therefore, primary acute undernutrition is mostly social rather than biomedical in origin, but it is also multifactorial. For example, poor water quality, sanitation and hygiene practices are increasingly believed to be the cause of the condition called “environmental enteropathy” that contributes to acute undernutrition in childhood [38].

Although this study provided insight on the temporal trends and overall spatial risk in undernutrition among children under five between 2012 and 2018 in Burkina Faso, this study is influenced by several limitations. Our study did not cover all the National Nutrition Surveys from 2009 to 2021, due to data unavailability, therefore our finding is only relevant for the studied period. The analysis relies on survey data but not a cohort, hence causality cannot be inferred.

Conclusion

The study found that undernutrition prevalence remains high and marked spatial disparities. Overall, there is a decreased of prevalence over the year. It is important to note that we found low risk area where the control effort need to maintained and reinforced. The high-risk area, need to be allocated more resources and reinforce the control effort. Morbidity (fever and diarrhea) constitutes important associated factors and need prompt and effective management with particular focus on children over 6 months. Further research should include birth cohort to allow causality inference for undernutrition in Burkina Faso. Additionally, surveillance system should be implemented towards IDPs. Our study showed that secondary analysis of national surveillance data has the potential to inform public health action and refinement of intervention.

For peer review only

Acknowledgements

The authors would like to thank the Ministry of Health and Public Hygiene for allowing access to the data.

Funding

This research was funded by the National Institute for Health Research (NIHR) (17/63/154) using UK aid from the UK Government to support global health research. The views expressed in this publication are those of the author(s) and not necessarily those of the NIHR or the UK Department of Health and Social Care.

Conflict of interest

The authors declare that they have no competing interests.

Authorship

H.S., M.L.N., P.R.B., H.T. and K.W. designed the WP5 study. H.S., P.R.B., H.T. and K.W. supervised the implementation of the study and guided the manuscript conception. P.R.B. and TR analysed the data, carried out the literature review and drafted the paper. E.B, S.K, E.C, B.O reviewed and approved the final manuscript.

Ethics

This study is part of the of the INPreP3 Study and has been approved by the National Health Ethics Committee in Burkina Faso, Approval number 2018-12-156.

Data sharing

Access to data is available upon request to the Ministry of Health and Public Hygiene.

References

[1] FAO, IFAD, UNICEF, and WFP, "The State of Food Security and Nutrition in the World 2021. Transforming food systems for food security, improved nutrition and affordable healthy diets for all," Rome, 2021.

[2] Development Initiatives, "The burden of malnutrition - Global Nutrition Report," Bristol, 2018.

[3] M. Myatt *et al.*, "Children who are both wasted and stunted are also underweight and have a high risk of death: A descriptive epidemiology of multiple anthropometric deficits using data from 51 countries," *Arch. Public Heal.*, vol. 76, no. 1, Jul. 2018.

[4] UNICEF / WHO / World Bank Group, "UNICEF-WHO-The World Bank: Joint child malnutrition estimates – levels and trends – 2021 edition - UNICEF DATA," 2021.

[5] "Children: improving survival and well-being." .

[6] D. L. Pelletier, E. A. Frongillo, and J. P. Habicht, "Epidemiologic evidence for a potentiating effect of malnutrition on child mortality," *Am. J. Public Health*, vol. 83, no. 8, pp. 1130–1133, 1993.

[7] R. E. Black *et al.*, "Maternal and child undernutrition: global and regional exposures and health consequences," *Lancet*, vol. 371, no. 9608, pp. 243–260, 2008.

[8] R. E. Black *et al.*, "Maternal and child undernutrition and overweight in low-income and middle-income countries," *Lancet*, vol. 382, no. 9890, pp. 427–451, 2013.

For peer review only

[9] J. McFie and H. F. Welbourn, “Effect of Malnutrition in Infancy on the Development of Bone, Muscle and Fat,” *J. Nutr.*, vol. 76, no. 2, pp. 97–105, Feb. 1962.

[10] H. Darsene, A. Geleto, A. Gebeyehu, and S. Meseret, “Magnitude and predictors of undernutrition among children aged six to fifty nine months in Ethiopia: A cross sectional study,” *Arch. Public Heal.*, vol. 75, no. 1, Jul. 2017.

[11] L. Shahrin, M. J. Chisti, and T. Ahmed, “3.1 Primary and secondary malnutrition,” *World Rev. Nutr. Diet.*, vol. 113, pp. 139–146, 2015.

[12] “United Nations Sustainable Development Goals (SDGs).” .

[13] C. M. McDonald *et al.*, “The effect of multiple anthropometric deficits on child mortality: Meta-analysis of individual data in 10 prospective studies from developing countries,” *Am. J. Clin. Nutr.*, vol. 97, no. 4, pp. 896–901, Apr. 2013.

[14] T. Khara, M. Mwangome, M. Ngari, and C. Dolan, “Children concurrently wasted and stunted: A meta-analysis of prevalence data of children 6–59 months from 84 countries,” *Matern. Child Nutr.*, vol. 14, no. 2, Apr. 2018.

[15] M. Garenne *et al.*, “Incidence and duration of severe wasting in two African populations,” *Public Health Nutr.*, vol. 12, no. 11, pp. 1974–1982, Feb. 2009.

[16] Davycas, UNICEF, WFP, and Ministry of Health, “Nutrition survey in communities and sites with internally displaced persons (IDPs) in Burkina Faso,” Ouagadougou, 2020.

[17] V. Ridde, L. Lechat, and I. B. Meda, “Terrorist attack of 15 January 2016 in Ouagadougou: how resilient was Burkina Faso’s health system?,” *BMJ Glob. Heal.*, vol. 1, no. 1, p. e000056, 2016.

[18] International Crisis Group, “The Social Roots of Jihadist Violence in Burkina Faso’s North | Crisis Group,” *Africa Report N°254*, 2017. .

[19] Save the Children, DS/DRS de Kaya, and Lionella Fieschi, “Prise en charge Communautaire de la Malnutrition Aiguë (PCMA): Évaluation Semi-Quantitative de l’Accessibilité et de la Couverture (SQUEAC),” Kaya, Burkina Faso, 2010.

[20] Ministry of Health, “Guide pour le suivi et la gestion de la prise en charge de la malnutrition aiguë,” Ouagadougou, Burkina Faso, 2009.

[21] Institut National de la statitistique et de la démographie, “Cinquième Recensement Général de la Population et de l’Habitation du Burkina Faso,” no. août, p. 400, 2022.

[22] The World Factbook, “The World Factbook — Central Intelligence Agency. Burkina Faso population projection.” .

[23] Ministère de la Santé Burkina Faso (DGESS), “Annuaire statistique 2020,” Ouagadougou, Apr. 2020.

[24] Ministère de la Santé, “Enquête Nutritionnelle Nationale (SMART-2020),” Ouagadougou, Burkina Faso, 2020.

[25] World Health Organization, “Length/height-for-age, weight-for-age, weight-for-length, weight-for-height and body mass index-for-age Methods and development,” 2006.

[26] Y. Zida and S. H. Kambou, “Cartographie de la pauvreté et des inégalités au Burkina Faso,” 2014.

[27] P. K. Masibo and D. Makoka, “Trends and determinants of undernutrition among young Kenyan children: Kenya Demographic and Health Survey; 1993, 1998, 2003 and 2008-2009.,” *Public Health Nutr.*, vol. 15, no. 9, pp. 1715–1727, 2012.

[28] E. Barankanira, N. Molinari, P. Msellati, C. Laurent, and K. A. Bork, “Stunting among children under 3 years of age in Côte d’Ivoire: Spatial and temporal variations between 1994 and 2011,” *Public Health Nutr.*, vol. 20, no. 9, pp. 1627–1639, 2017.

[29] M. De Onis, M. Blössner, and E. Borghi, “Prevalence and trends of stunting among pre-school children, 1990-2020,” *Public Health Nutr.*, vol. 15, no. 1, pp. 142–148, 2012.

[30] B. J. Akombi, K. E. Agho, J. J. Hall, D. Merom, T. Astell-Burt, and A. M. N. Renzaho, “Stunting and severe stunting among children under-5 years in Nigeria: A multilevel analysis,” *BMC Pediatr.*, vol. 17, no. 1, pp. 1–16, 2017.

[31] S. Thurstans *et al.*, “Boys are more likely to be undernourished than girls: A systematic review

For peer review only

and meta-analysis of sex differences in undernutrition," *BMJ Glob. Heal.*, vol. 5, no. 12, 2020.
[32] Y. Y. Yang *et al.*, "Trends and determinants of stunting among under-5s: Evidence from the 1995, 2001, 2006 and 2011 Uganda Demographic and Health Surveys," *Public Health Nutr.*, vol. 21, no. 16, pp. 2915–2928, 2018.
[33] M. T. Ruel *et al.*, "Age-based preventive targeting of food assistance and behaviour change and communication for reduction of childhood undernutrition in Haiti: a cluster randomised trial," *Lancet (London, England)*, vol. 371, no. 9612, pp. 588–595, 2008.
[34] C. Desmond *et al.*, "Investing in human development and building state resilience in fragile contexts : A case study of early nutrition investments in Burkina Faso," *PLOS Glob. PUBLIC Heal.*, vol. 3, no. 3, pp. 1–15, 2023.
[35] Y. Sanoussi, B. O. Ahinkorah, A. Banke-Thomas, and S. Yaya, "Assessing and decomposing inequality of opportunity in access to child health and nutrition in sub-Saharan Africa: Evidence from three countries with low human development index," *Int. J. Equity Health*, vol. 19, no. 1, pp. 1–16, 2020.
[36] C. J. A. Bradshaw *et al.*, "Testing the socioeconomic and environmental determinants of better child-health outcomes in Africa: A cross-sectional study among nations," *BMJ Open*, vol. 9, no. 9, 2019.
[37] V. Dipasquale, U. Cucinotta, and C. Romano, "Acute Malnutrition in Children: Pathophysiology, Clinical Effects and Treatment," *Nutrients*, vol. 12, no. 8, pp. 1–9, Aug. 2020.
[38] A. Gelli *et al.*, "Improving diets and nutrition through an integrated poultry value chain and nutrition intervention (SELEVER) in Burkina Faso: Study protocol for a randomized trial," *Trials*, vol. 18, no. 1, Sep. 2017.

For peer review only

List of figures

Figure 1. Overall province-specific odd ratios (province random effect) of stunting (a), underweight (b) and wasting (c) among study population.
Maps created by Rouamba T. et al., 2021.

Figure 2. Overall region-specific odd ratios (region random effect) of stunting (a), underweight (b) and wasting (c) among study population.

For peer review only

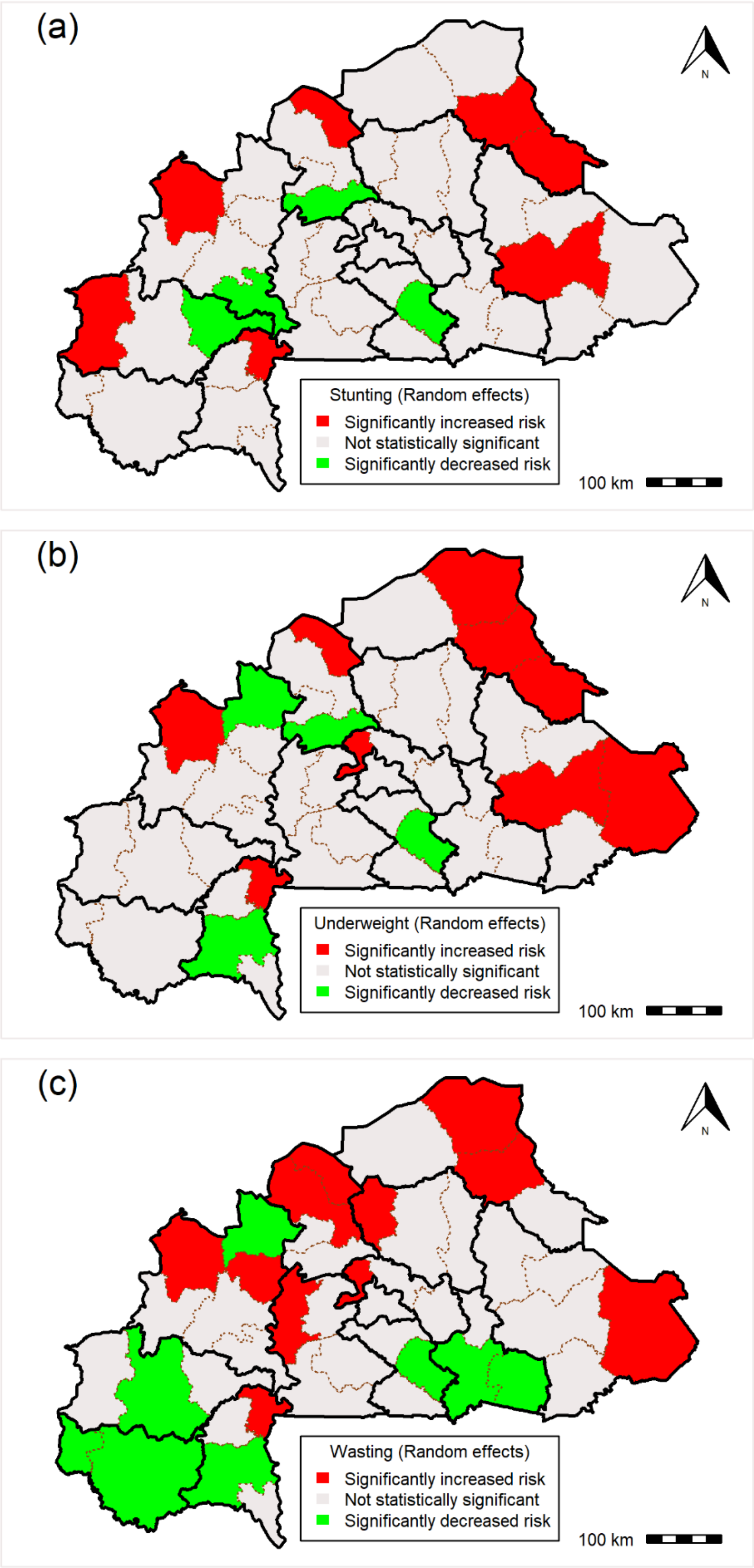

Figure 1. Overall province-specific odd ratios (province random effect) of stunting (a), underweight (b) and wasting (c) among study population.
Maps created by Rouamba T. et al., 2021.

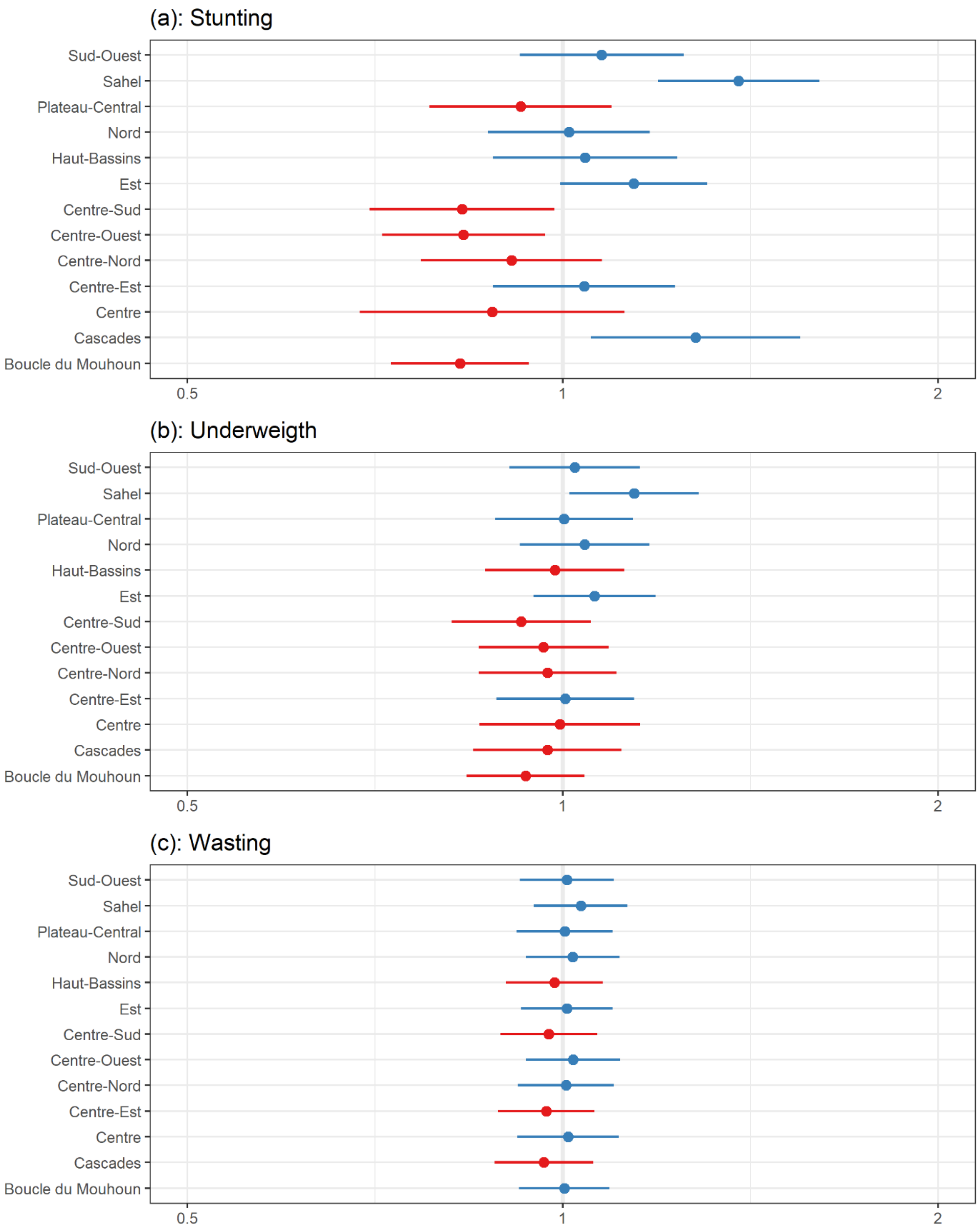

Figure 2. Overall region-specific odd ratios (region random effect) of stunting (a), underweight (b) and wasting (c) among study population.

General linear mixed models (multilevel models) with binomial distribution were used to quantify the association of independent variables with each study outcome variable (stunting, underweight and wasting). The general linear mixed model contains fixed and random effects and was formulated as follow:

$$\boldsymbol{Y_{ij}} \sim Bernoulli\ (\pi_{ij})$$

$$logit\ (\pi_{ij}) = log\left[\frac{\pi_{ij}}{1-\pi_{ij}}\right] = \beta_0 + \sum_{p=1}^{P} \beta_p x_{ij} + \sum_{q=1}^{Q} \beta_q z_j + \omega_j + \varepsilon_i$$

In this general linear model, $\boldsymbol{Y_{ij}}$ represent stunting, underweight or wasting for a child i in a cluster j. The logit (π_{ij}) was used to model the probability of success (i.e., occurrence of undernutrition) as a linear combination of observed individual characteristics (x_{ij}) and contextual characteristics (z_j) associated with an unobserved specific effect (random effect) of the province and region (ω_j). The model contains a random error for child *i* (ε_i) that does not depend to the province/region nor year. The fixed effect is composed of $\boldsymbol{\beta_0}$ (intercept), $\boldsymbol{\beta_p}$ (vector of regression coefficients associated with the individual-level variables) and $\boldsymbol{\beta_q}$ (vector of regression coefficients associated with the cluster-level variables). The unobserved specific effect or random effect $(\boldsymbol{\omega_j})$ has two components, province specific effect and region specific effect. These cluster specific effects were considered as province and region specific risk.

All statistical analyses were performed with R statistical software (R Development Core Team, R Foundation for Statistical Computing, Vienna, Austria). The descriptive statistics that accounted for the complex survey design and sampling weights were performed using “survey” package, regression models were fitted using the “lme” package.

Model Diagnostics

We assume the response was drawn from a binomial distribution the residual are independent and normally distributed. The random effects are also, independent and normally distributed. The model diagnostics was check by generating a diagnostic that plots the fitted or predicted values against the residuals.

For peer review only

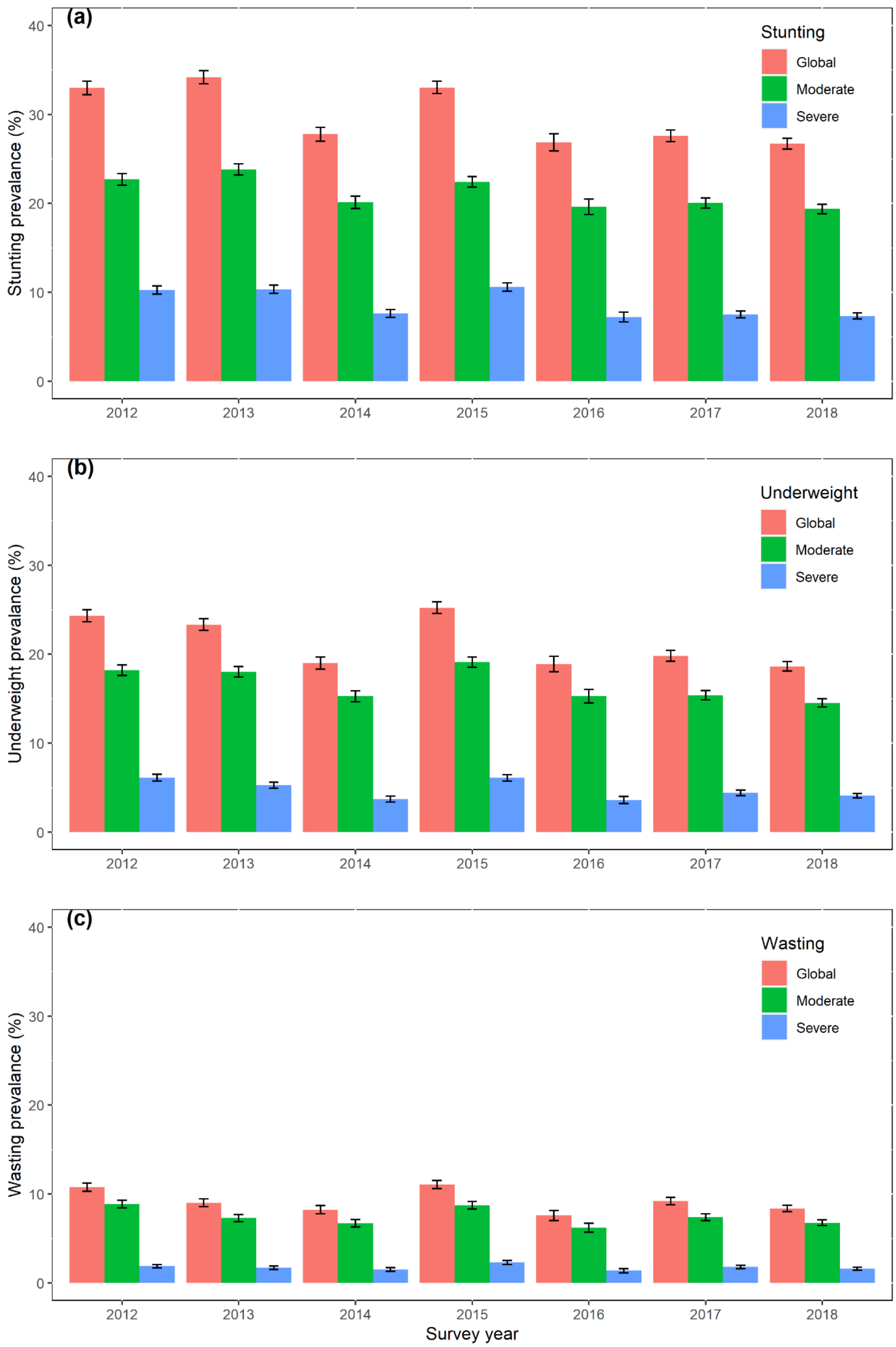

Supplemental Figure 1. Prevalence of stunting (a), underweight (b) and wasting (c). The plots demonstrate changes from 2012 to 2018. The error bars mark the 95% confidence intervals in panels a, b and c.

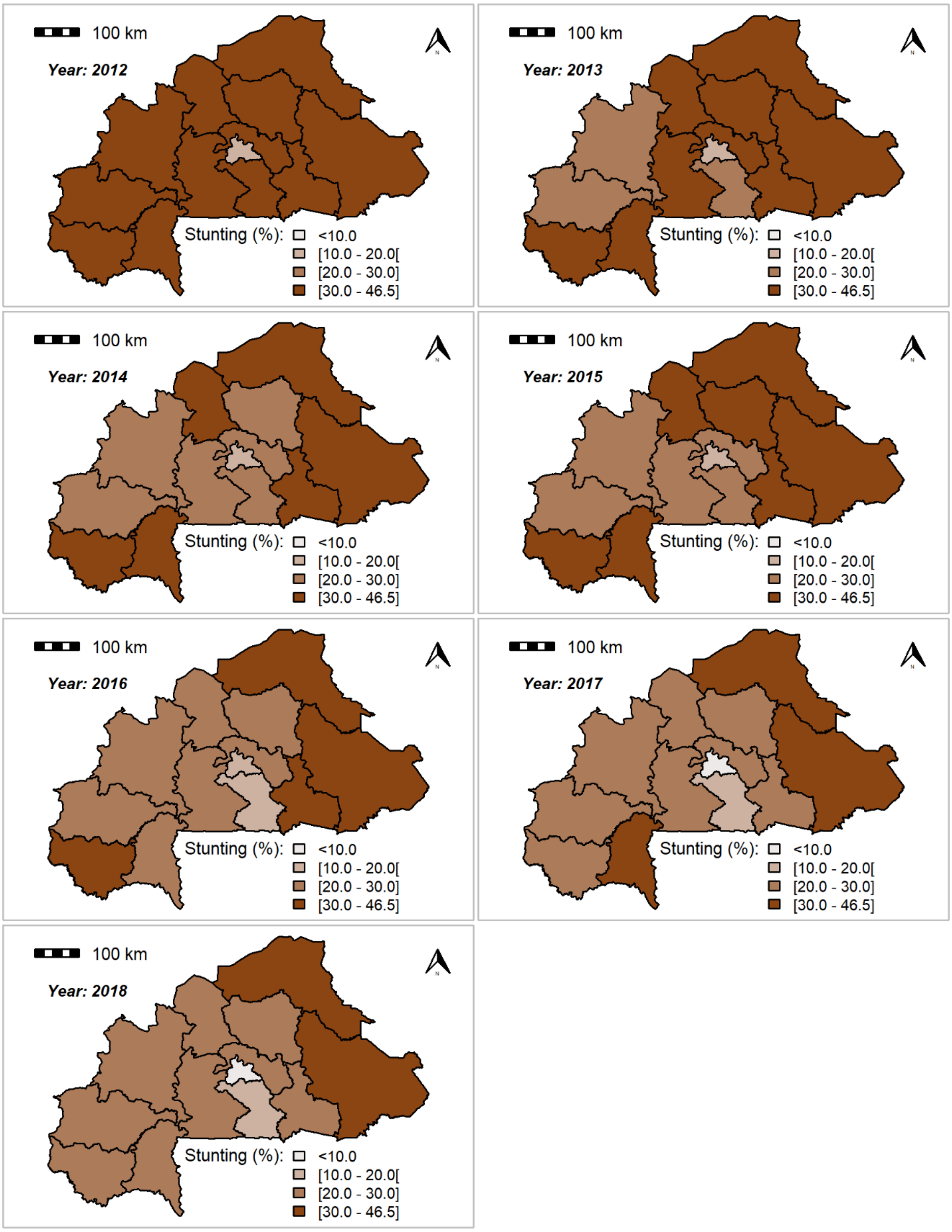

Supplemental Figure 2. Maps of geographical distribution at regional level of stunting among children aged 0–59 months surveyed in Burkina Faso, 2012 to 2018.
Maps created by Rouamba T. et al., 2021.

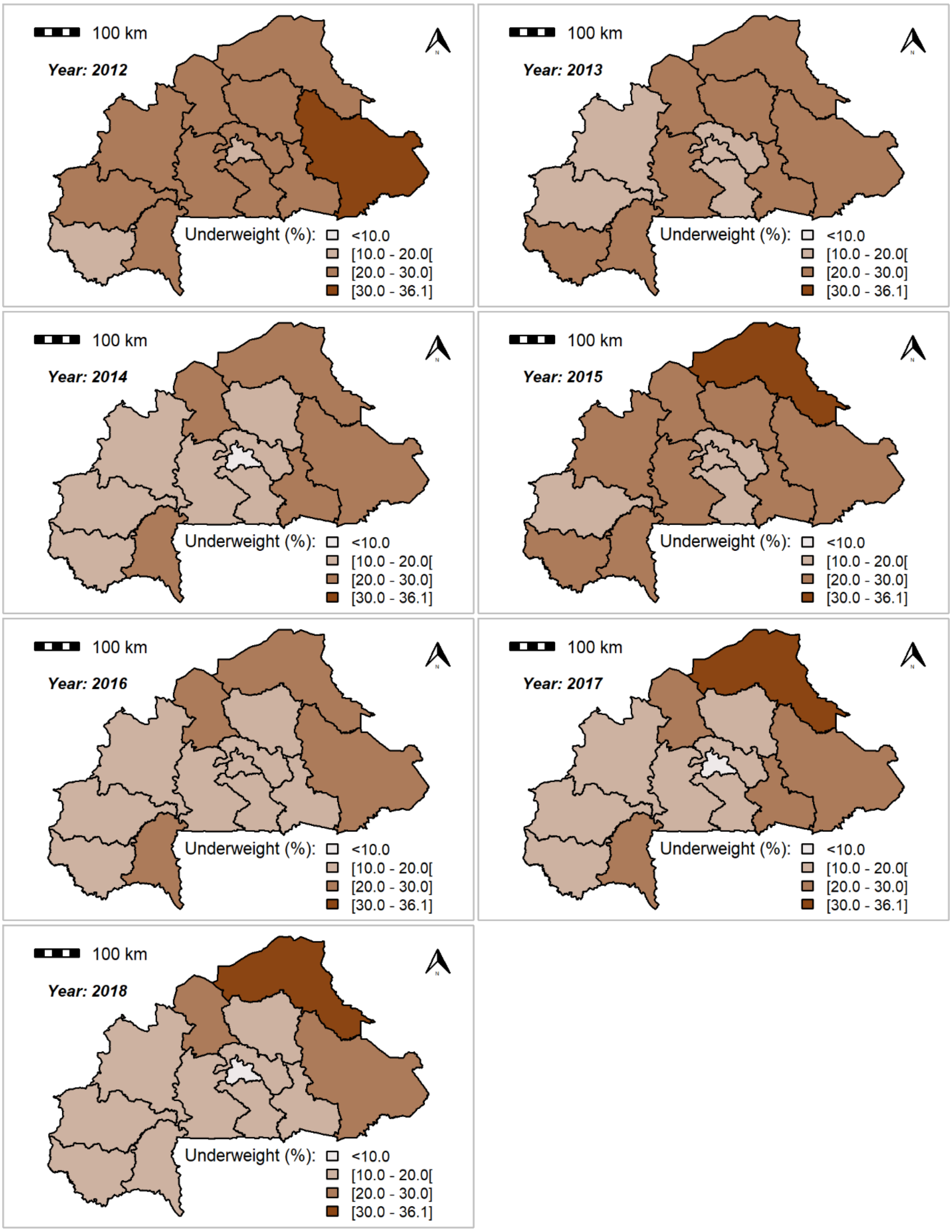

Supplemental Figure 3. Maps of geographical distribution at regional level of underweight among children aged 0–59 months surveyed in Burkina Faso, 2012 to 2018.
Maps created by Rouamba T. et al., 2021.

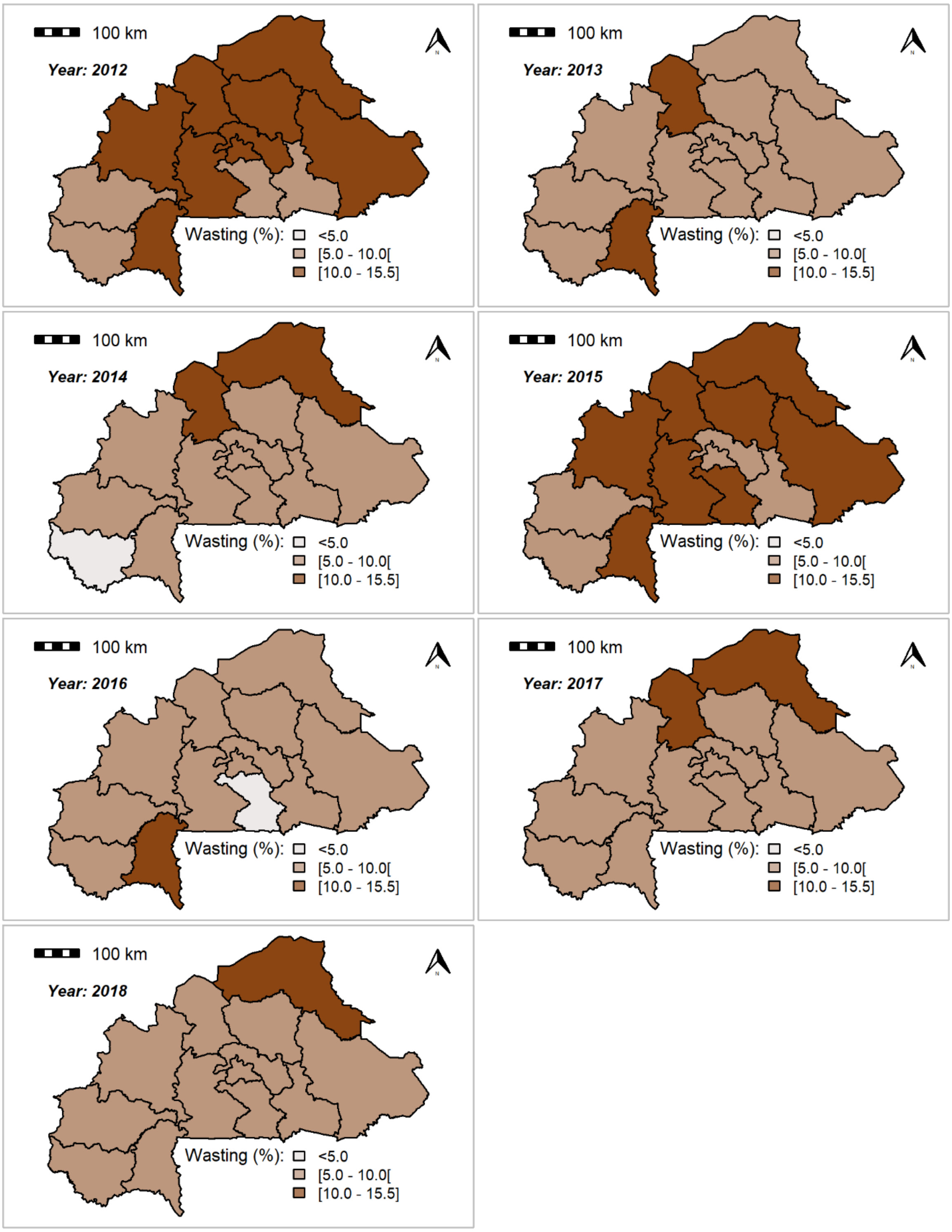

Supplemental Figure 4. Maps of geographical distribution at regional level of wasting among children aged 0–59 months surveyed in Burkina Faso, 2012 to 2018.
Maps created by Rouamba T. et al., 2021.

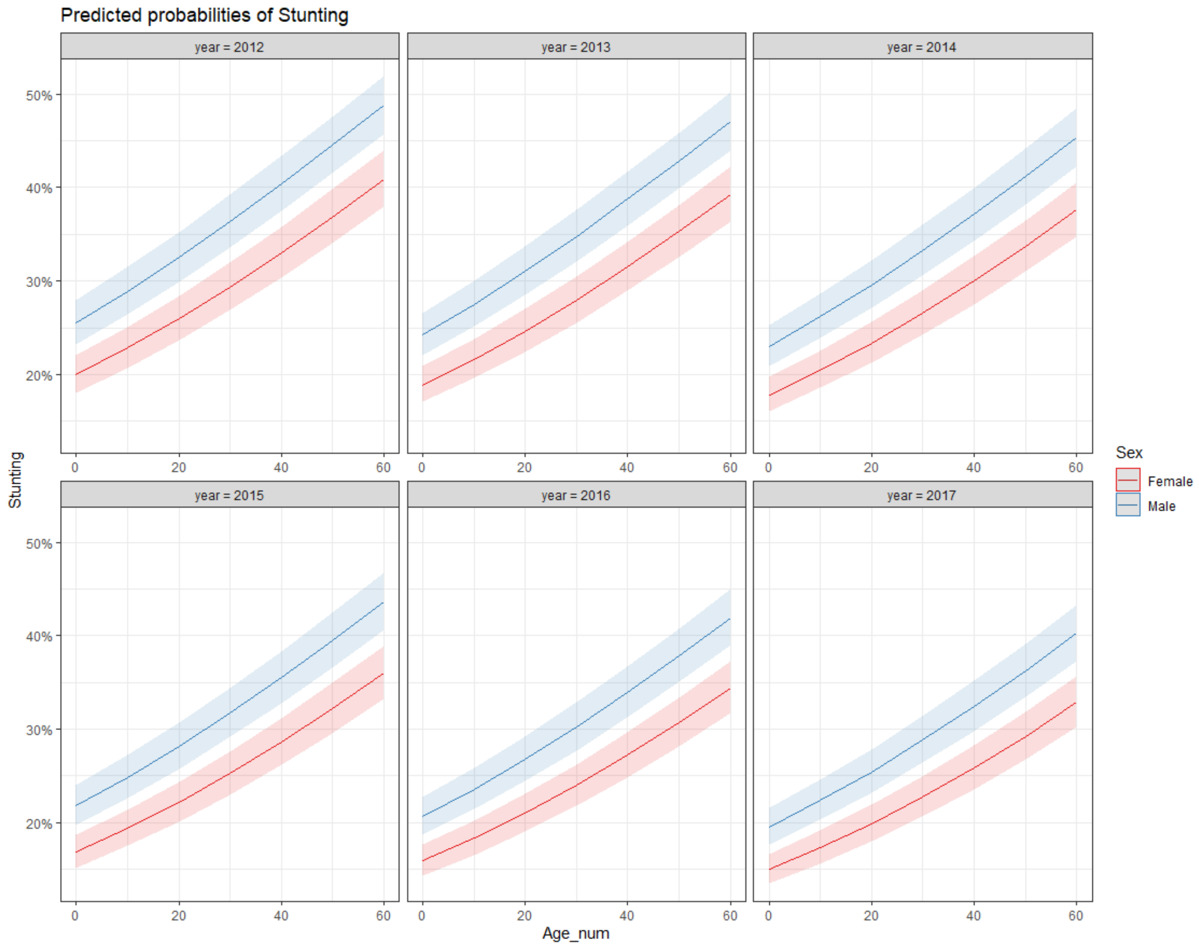

Supplemental Figure 5. Predicted probabilities of stunting according to the child age and stratified by gender in Burkina Faso, from 2012 to 2017.

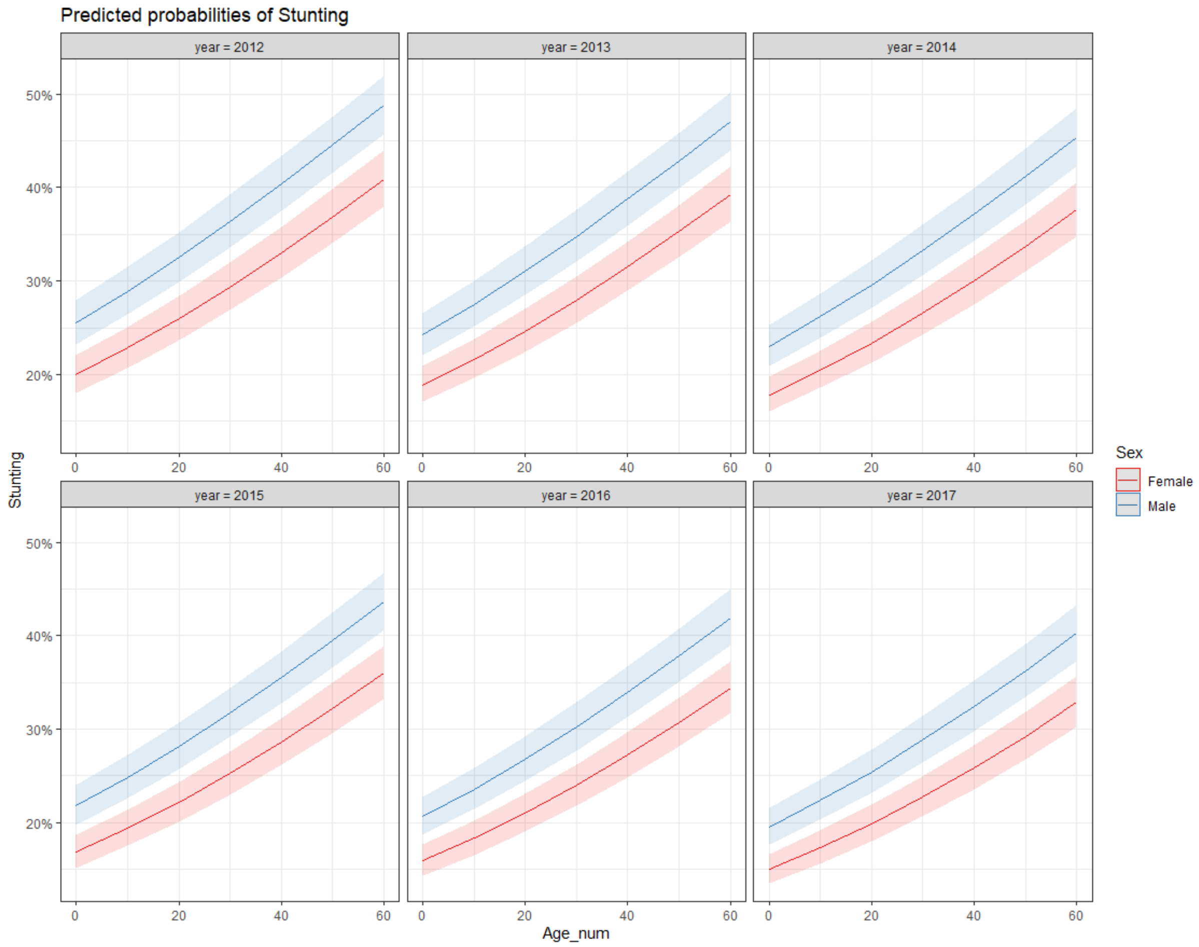

Supplemental Figure 5. Predicted probabilities of stunting according to the child age and stratified by gender in Burkina Faso, from 2012 to 2017.

For peer review only - http://bmjopen.bmj.com/site/about/guidelines.xhtml

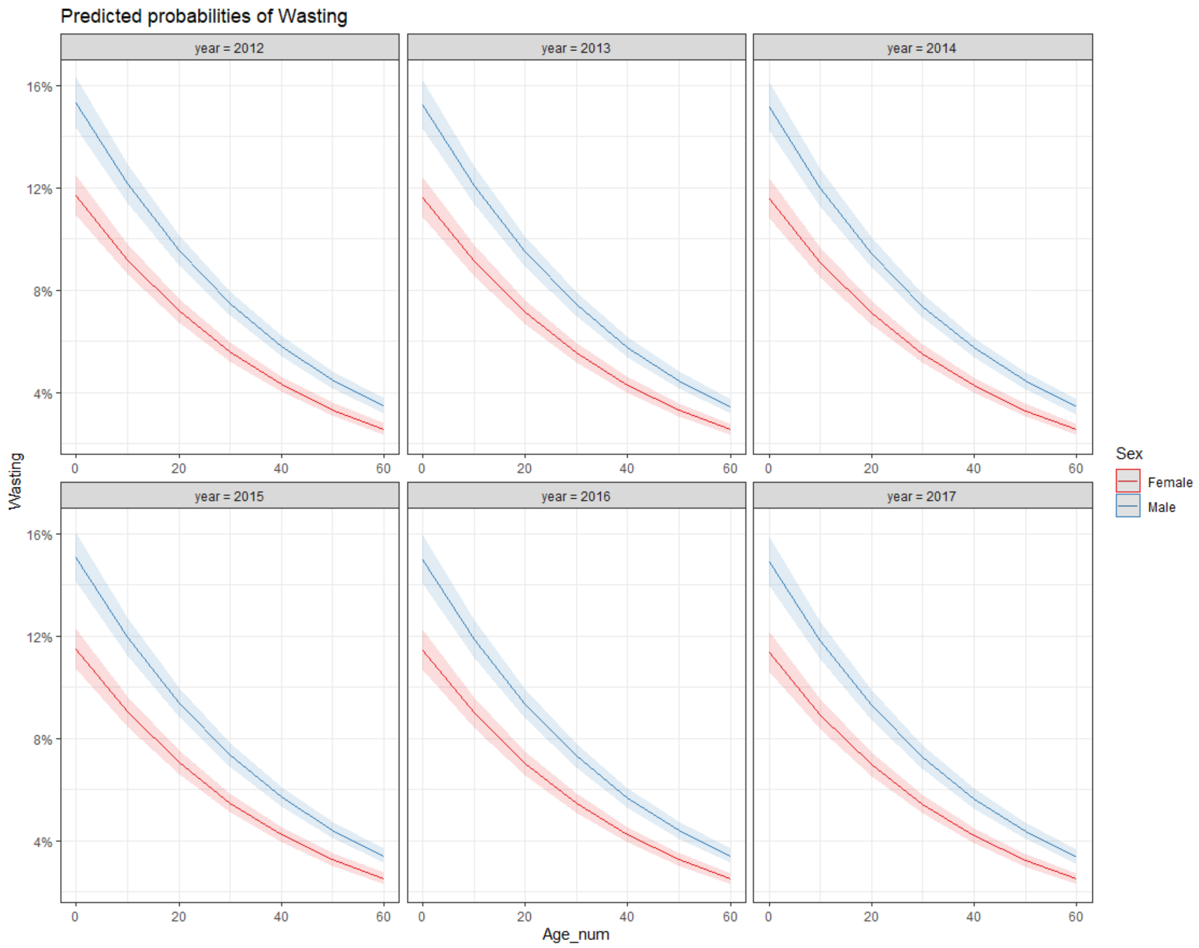

Supplemental Figure 7. Predicted probabilities of wasting according to the child age and stratified by gender in Burkina Faso, from 2012 to 2017.

view only

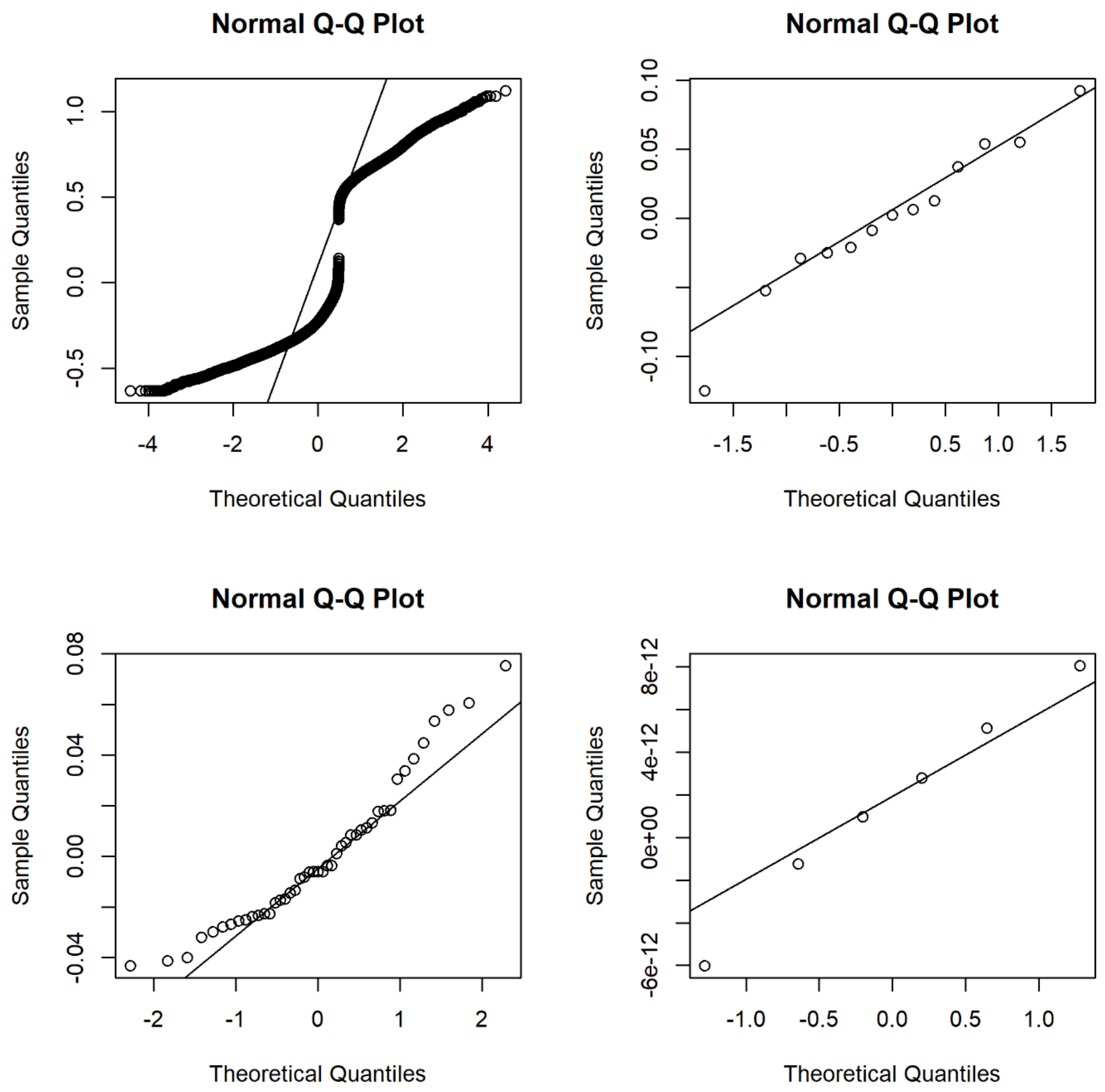

Supplemental Figure 8. Diagnostic plots for stunting multilevel model. Upper left: QQ plot of the residuals; upper right: QQ plot of the random effects (region); lower left: QQ plot of the random effects (province); lower right: QQ plot of the random effects (year)

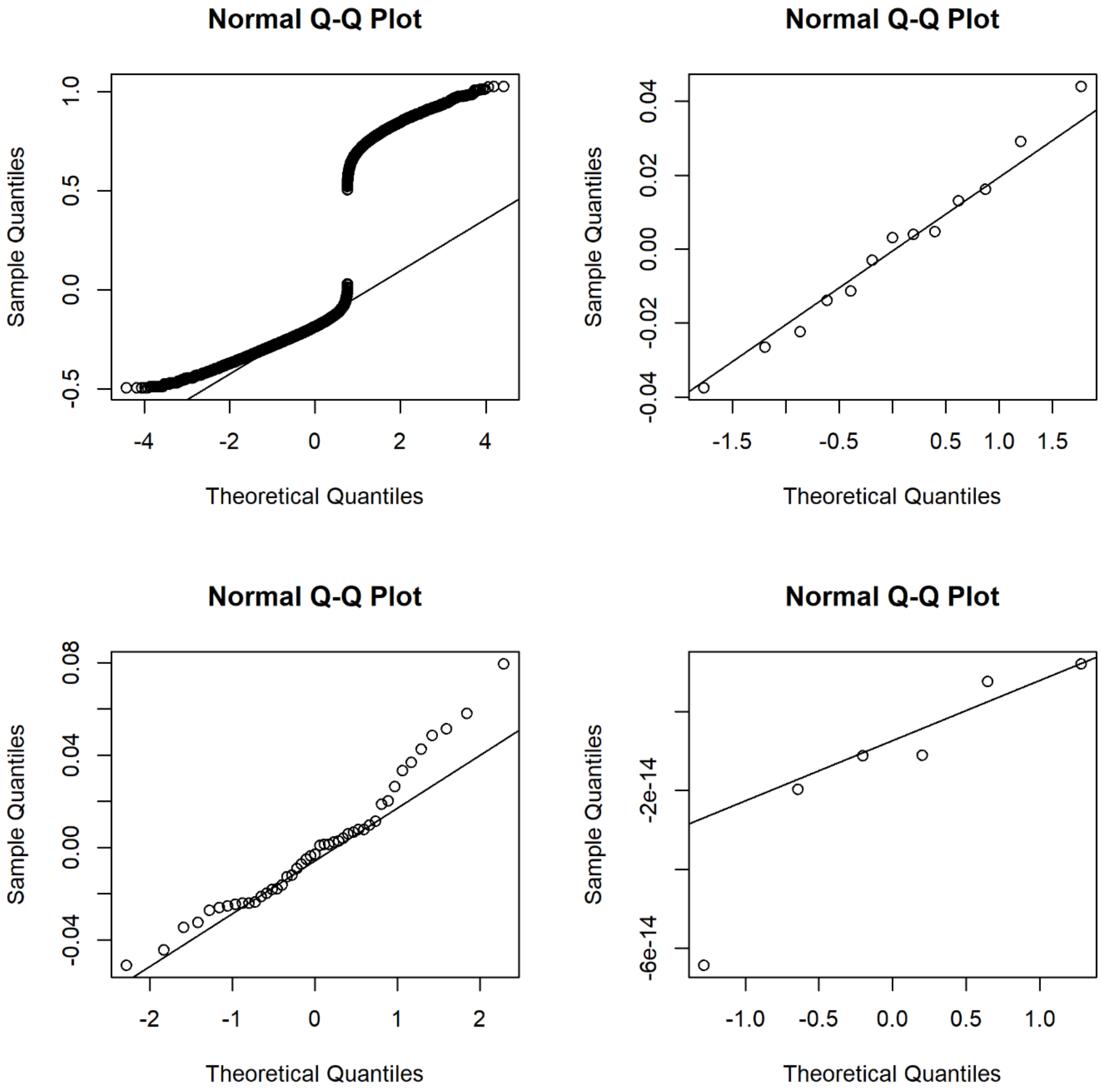

Supplemental Figure 9. Diagnostic plots for underweight multilevel model. Upper left: QQ plot of the residuals; upper right: QQ plot of the random effects (region); lower left: QQ plot of the random effects (province); lower right: QQ plot of the random effects (year)

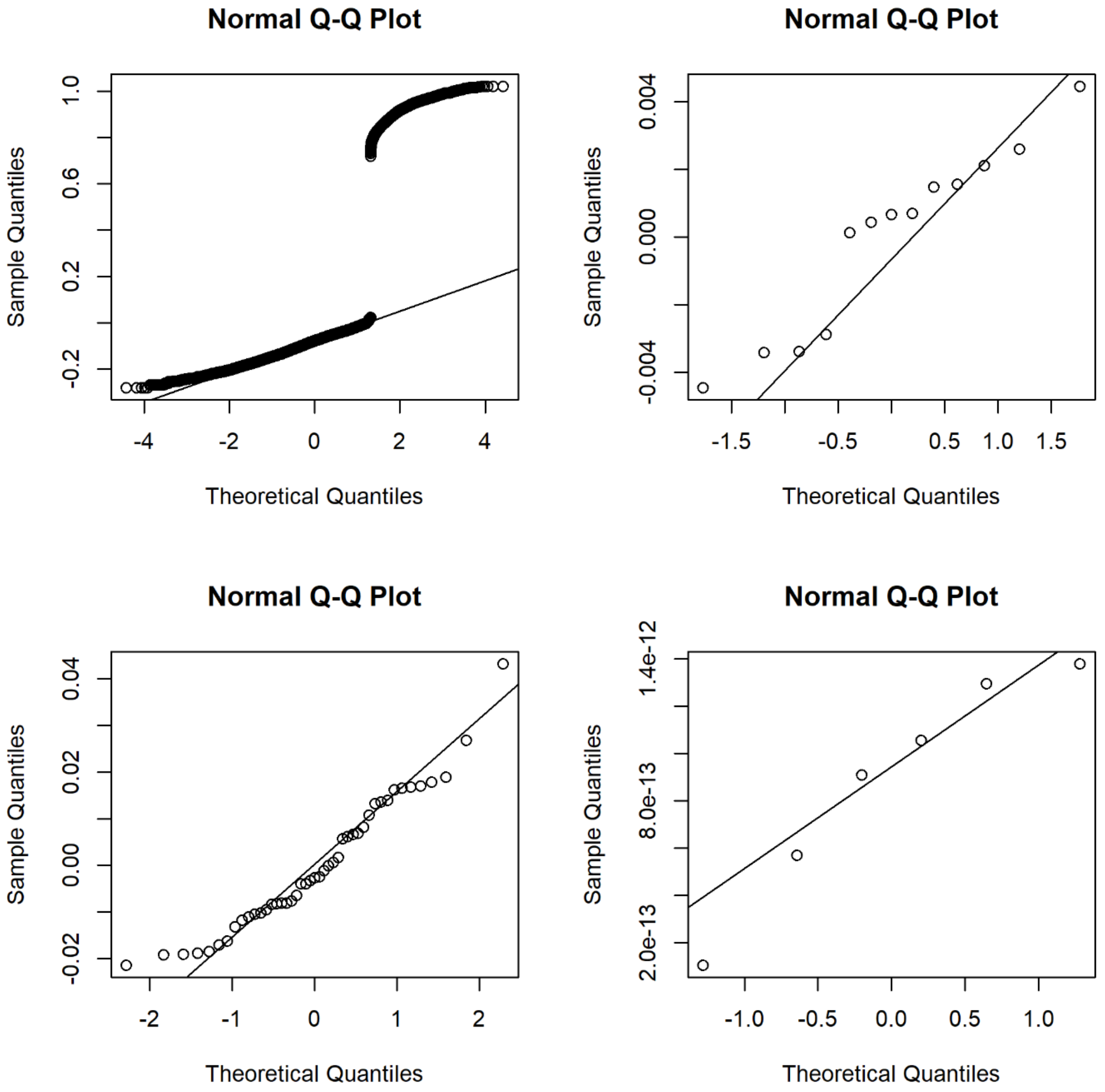

Supplemental Figure 10. Diagnostic plots for wasting multilevel model. Upper left: QQ plot of the residuals; upper right: QQ plot of the random effects (region); lower left: QQ plot of the random effects (province); lower right: QQ plot of the random effects (year)
